# Revision of the Structure and Total Synthesis of Topsentin C

**DOI:** 10.1055/s-0036-1588731

**Published:** 2017-02-23

**Authors:** Nikita E. Golantsov, Alexey A. Festa, Alexey V. Varlamov, Leonid G. Voskressensky

**Affiliations:** Peoples’ Friendship University of Russia (RUDN University)6 Miklukho-Maklaya St., Moscow 117198Russian Federationgolantsov_ne@pfur.ru

**Keywords:** bisindole alkaloid, topsentin, hamacanthin, spongotine, diamine, hydroxylamine

## Abstract

An efficient synthetic approach to access (indol-3-yl)ethane-1,2-diamines with a protecting group at the indole N atom from readily available 3-(2-nitrovinyl)indoles is reported. This approach includes solvent-free conjugate addition of *O* -pivaloylhydroxylamines to 1-Boc-3-(2-nitrovinyl)indoles followed by mild reduction of the adducts. The obtained (indol-3-yl)ethane-1,2-diamines are convenient synthetic precursors for several classes of marine alkaloids. The first total synthesis of racemic topsentin C, a secondary metabolite from *Hexadella* sp., based on this approach is reported. The initially proposed structure for topsentin C has been revised.

Secondary metabolites from marine invertebrates continue to be an attractive research topic because new structures and compounds with useful biological activity can be discovered. 1 A whole series of alkaloids containing the (indol-3-yl)ethane-1,2-diamine moiety in their structures and their aromatized derivatives were isolated from deep-water sponges in the last 30 years. 2 In particular, spongotines ( **1** ) and topsentins ( **2** ) contain two indoles connected through imidazoline or imidazole linker. Two indole substituents in the structures of hamacanthins ( **3** ) and dragmacidins ( **4** ) are bonded to dihydropyrazinone and piperazine rings, respectively (Figure 1 ). The (indol-3-yl)ethane-1,2-diamine moiety in several alkaloids of the examined group contains one or two methyl groups; for example, dragmacidins A and B ( **4** ) and topsentin C. The latter compound was isolated from *Hexadella* sp., and its structure was assigned to imidazoline derivative **5a** (Figure 2 ). 3 Furthermore, the alkaloids could contain one or more Br atoms, which in general is characteristic of marine secondary metabolites. 4 Notably, the 1,2-diaminoethyl group in the indole 3-position, in contrast to 2-aminoethyl, is uncharacteristic for terrestrial indole alkaloids.

**Figure 1:**
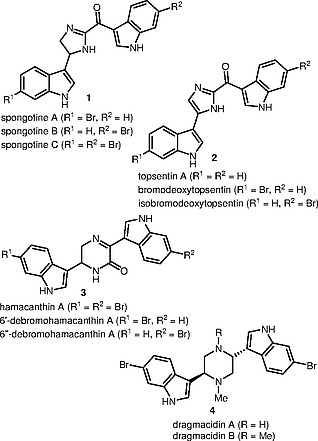
Several marine alkaloids containing an (indol-3-yl)ethanediamine fragment and their aromatized derivatives

Total syntheses of many of the natural products from this group have been reported; 2 5 6 however, no synthesis of topsentin C has been reported. Compounds exhibiting antibacterial, cytotoxic, antiviral, and fungicidal properties were discovered among these alkaloids and their synthetic analogues. 2,7

**Figure 2:**
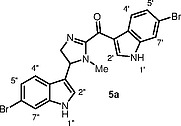
Proposed structure of topsentin C

(Indol-3-yl)ethane-1,2-diamines could be convenient synthetic precursors of topsentins, spongotines, and hamacanthins. 5a b c Furthermore, these diamines are of independent interest because their simple derivatives were recently shown to be capable of preventing the development of resistance to fluoroquinolone antibiotics in *Staphylococcus aureus* . 8

Only two synthetic approaches to (indol-3-yl)ethane-1,2-diamines have been published and neither of them allows the corresponding *N* ^1^ -methyl derivatives to be produced. 5a b c It was also reported that diamines of this type with an unsubstituted indole N atom are relatively stable only as the salts. 5a We propose a convenient preparative synthetic approach to (indol-3-yl)ethane-1,2-diamines ( **6** ) with a protected indole N atom that is based on mild reduction of **7** , the addition product of *O* -pivaloylhydroxylamines ( **8** ) and 3-nitrovinylindoles ( **9** ) (Scheme 1 ). The proposed method has been used for the total synthesis of topsentin C, the previously proposed structure of which has been revised by us.


**Scheme 1** Our Synthetic approach to (indol-3-yl)ethanediamine **6**


Starting nitrovinylindoles **9** , with *tert* -butoxycarbonyl-protected indole N atoms, were synthesized from the corresponding indoles by formylation using *N* , *N* -dimethylform­amide (DMF) and SOCl _2_ followed by condensation of the obtained aldehydes with nitromethane and addition of the protecting group in the presence of 4-( *N* , *N* -dimethylamino)pyridine (DMAP) (Scheme 2 ). We also prepared 1-acetyl-3-[( *E* )-2-nitrovinyl]-1 *H* -indole ( **9f** ) and 1-methyl-3-[( *E* )-2-nitrovinyl]-1 *H* -indole ( **9g** ) according to described procedures. 9 10


**Scheme 2** Synthesis of 3-nitrovinylindoles **9** ; yields of three stages are shown



**Scheme 3** Attempts to convert nitrovinylindole **9b** into the corresponding indolic diamines by using aliphatic amines or *O* -benzylhydroxylamine


Reduction of the adducts of α,β-unsaturated nitrocompounds with amines, *O* -alkylhydroxylamines or azide anion was proposed earlier for the synthesis of vicinal diamines. 11 12 13 The first type of adduct was unstable, although they could be isolated as the more stable salts. 11 Adducts with *O* -alkylhydroxylamines or azide anion were more stable. 12 13 However, catalytic hydrogenation or heating with Zn dust in HOAc was required to cleave the hydroxylamine N–O bond. 12 Catalytic hydrogenation was also used to reduce the azido group. 13 The proposed methods turned out to be ineffective for the synthesis of Br-containing (indol-3-yl)ethane-1,2-diamines **6** . Thus, the products **10** from the reaction of methylamine or benzylamine with nitrovinylindole **9b** could not be isolated; starting **9b** was recovered and the reaction mixture formed a resin. Stable adduct **11** with *O* -benzylhydroxylamine was reduced as expected by H _2_ over Pd/C with hydrogenolysis of the C–Br bond to form diamine **6a** (Scheme 3 ).

Indolic adduct **11** decomposed upon heating with Zn in HOAc, and reduction with Zn under milder conditions was not complete. The yield of diamine **6b** was <15% even when the amount of Zn and the reaction time were increased; the main product was **12** (Scheme 3 ).

**Table:** **Table 1** Synthesis of Adducts **7**

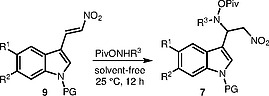							
Entry	Substrate	Product	R ^1^	R ^2^	R ^3^	PG	Yield (%)
1	**9a**	**7a**	H	H	H	Boc	94
2	**9b**	**7b**	Br	H	H	Boc	96
3	**9a**	**7c**	H	H	Me	Boc	97
4	**9b**	**7d**	Br	H	Me	Boc	89
5	**9c**	**7e**	H	Br	Me	Boc	98
6	**9d**	**7f**	OMe	H	Me	Boc	91
7	**9e**	**7g**	H	Cl	Me	Boc	97
8	**9f**	**7h**	H	H	Me	Ac	88
9	**9g**	– ^[a]^	H	H	Me	Me	0

^a^ Nitrovinylindole **9g** , with a methyl on the indole N atom, did not react.

*O* -Acylhydroxylamines have highly labile N–O bonds and have recently been used in synthetic procedures based on sigmatropic shifts with cleavage of N–O bonds 14 in addition to amination reaction. 15 Conjugate addition of *O* -acylhydroxylamines to electron-deficient alkenes has not yet been described, in contrast to *O* -alkylhydroxylamines. We decided to study the possibility of adding *O* -pivaloylhydroxylamine and its *N* -methyl derivative to nitrovinylindoles **9** followed by reduction of the resulting adducts. Derivatives of sterically hindered pivalic acid were chosen because they isomerize rather slowly into the corresponding hydroxamic acids, in contrast to the simpler *O* -acylhydroxylamines. 16

Hydrochlorides of *O* -pivaloylhydroxylamine and *N* -methyl- *O* -pivaloylhydroxylamine were synthesized by using the previously reported methods. 14a 16 17 The corresponding free bases were isolated immediately before performing the next step.

As it turned out, the reaction of nitrovinylindoles **9a** with *O* -pivaloylhydroxylamine ( **8a** , 1.5 equiv) in CH _2_ Cl _2_ was complete in 96 hours and gave target adduct **7a** . We also found that the solvent-free reaction was much faster. The reagents could be mixed and left overnight in a closed vessel. The nitrovinylindoles dissolved gradually, then crystals of the product formed. The solvent-free reaction was clearly advantageous from a green chemistry point of view. In this manner, we obtained the series of adducts **7a** – **h** in high yields (Table 1 ).

**Table:** **Table 2** Reduction of Adducts **7a** , **c**

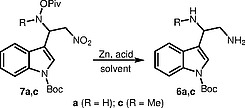							
Entry	R	Equiv. Zn	Acid (equiv)	Solvent	*t* (h)	*T* (°C)	Yield (%)
1	H	10	AcOH (150)	MeOH	2	0–20	34
2	H	10	AcOH (150)	MeOH	6	0–20	31
3	H	10	AcOH (150)	MeOH/H _2_ O/EtOAc	2	0–20	48
4	H	20	AcOH (150)	MeOH/H _2_ O/EtOAc	2	0–20	57
5	H	15	HCl (30)	MeOH/EtOAc	2	–10 to 5	65
6	H	15	HBr (30)	MeOH/EtOAc	2	–10 to 5	84
7	H	15	NH _4_ Br (15)	EtOH/H _2_ O/EtOAc	2	20	62
8	Me	15	HBr (30)	MeOH/EtOAc	2	–10 to 5	68
9	Me	20	HBr (40)	MeOH/EtOAc	6	–10 to 5	93

The next step was the reduction of adducts **7** . We decided to use Zn and acid, anticipating that their hydroxylamine N–O bond would undergo reductive cleavage at room or reduced temperature. Thus, the reduction of **7a** using Zn (10 equiv) and HOAc in MeOH afforded target diamine **6a** in 34% yield (Table 2 , entry 1).

The reaction proceeded rather quickly. However, it was accompanied by the formation of several unidentified side products. Increasing the reaction time did not lead to an increase in the yield of **6a** . We found that the yield could be increased by adding H _2_ O and EtOAc to the reaction mixture (keeping the solution homogeneous) and by doubling the amount of Zn (cf. Table 2 , entries 3 and 4). Replacing HOAc with concentrated HCl at reduced temperature led to a further increase in the yield (entry 5). Finally, the use of HBr (40%) allowed target diamine **6a** to be obtained with a very good yield (entry 6). It was also possible to conduct the reduction of adduct **7a** in the presence of NH _4_ Br (entry 7). The reduction of adduct **7c** , with a methyl group on the hydroxylamine N atom, was more difficult. However, increasing the reaction time and amount of Zn provided a high yield of diamine **6c** (entry 9). The developed method was extended to adducts **7b** and **7d** – **f** (Table 3 ). Boc derivatives gave the corresponding diamines **6b** and **6d** – **g** in good and high yields, whereas diamine **6h** , with an acetyl on the indole N atom, was unstable and decomposed even in solution.

**Table:** **Table 3** Synthesis of Diamines **6**

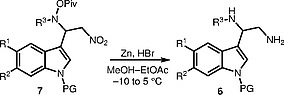							
Entry	Substrate	Product	R ^1^	R ^2^	R ^3^	PG	Yield (%)
1 ^a^	**7a**	**6a**	H	H	H	Boc	84
2 ^a^	**7b**	**6b**	Br	H	H	Boc	95
3 ^b^	**7c**	**6c**	H	H	Me	Boc	93
4 ^b^	**7d**	**6d**	Br	H	Me	Boc	86
5 ^b^	**7e**	**6e**	H	Br	Me	Boc	92
6 ^b^	**7f**	**6f**	OMe	H	Me	Boc	75
7 ^b^	**7g**	**6g**	H	Cl	Me	Boc	90
8 ^b^	**7h**	**6h**	H	H	Me	Ac	– ^c^

^a^ Reaction conditions: Zn (15 equiv), HBr (30 equiv), 2 h.

^b^ Reaction conditions: Zn (20 equiv), HBr (40 equiv), 6 h.

^c^ Decomposition of the product occurred.

Having established a convenient preparative method for *N* ^1^ -methyl(indol-3-yl)ethane-1,2-diamines, we focused on the total synthesis of the proposed structure for topsentin C ( **5a** ), which is related to spongotines **1** and other previously isolated topsentines. The imidazoline fragment of **5a** and its analogue **5b** , without a Br atom, was constructed by using the previously reported synthetic method for imidazolines that involved condensation of the vicinal diamines with aldehydes (including α-keto aldehydes) followed by oxidation of the resulting cyclic aminal. 5c 18 The required indolylglyoxals **14a** and **14b** were prepared from corresponding 3-acetylindoles **15a** and **15b** through iodination followed by Kornblum oxidation (Scheme 4 ). 19


**Scheme 4** Synthesis of spongotine analogues **5a** and **5b**


The aforementioned syntheses of indolylglyoxals **14a** and **14b** , condensations with diamines **6c** and **6e** , and subsequent oxidations to imidazolines **16a** and **16b** were carried out in one pot. This made the developed procedure attractive for preparative reactions. The protecting group could be removed to afford **5a** and **5b** . As it turned out, the spectral characteristics of **5a** synthesized by us and the characteristics of topsentin C that was isolated from the natural source, differed dramatically. Therefore, the initially proposed structure of topsentin C had to be revised. Thus, the synthesized **5a** was a methylated spongotine C derivative that has not yet been observed in nature. The developed method enables analogues of spongotines and top­sentins to be synthesized to study their biological properties, which are known to change abruptly if even a single methyl is added to the molecule. 17

We assumed that natural topsentin C was structurally related to hamacanthins A ( **3** ) but not spongotines ( **1** ), and was the 1-methyl derivative of hamacanthin A **17a** (Figure 3 ), 20 which should have a set of NMR signals similar to that of **5a** .

**Figure 3:**
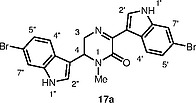
Revised structure of topsentin C


**Scheme 5** Synthesis of topsentin C and its analogue



**Scheme 6** Mechanism of dihydropyrazinone ring formation


We synthesized bis(indolyl)dihydropyrazinones **17a** and **17b** through cyclization of diamines **6e** and **6c** with indoleglyoxylic acid chlorides **18a** and **18b** to confirm this hypothesis (Scheme 5 ).

**Table:** **Table 4** ^1^ H NMR Data for Natural Topsentin C and for Compounds **17a** and **5a** ^a^

^1^ H	**17a**	Topsentin C ^3^	**5a**
N(1)CH _3_	3.04 (s)	3.05 (s)	2.82 (s)
CHC * H _a_* H _b_	4.26 (dd, *J* = 16.5, 5.5)	4.27 (dd, *J* = 16.5, 5.3)	3.91 (dd, *J* = 15.3, 10.3)
CHCH _a_ *H*_b_	4.41 (dd, *J* = 16.5, 5.2)	4.41 (dd, *J* = 16.5, 5.2)	4.31 (dd, *J* = 15.3, 11.3)
C *H* CH _a_ H _b_	5.15 (dd, *J* = 5.5, 5.2)	5.16 (ddd, *J* = 5.3, 5.2, <1)	4.89 (dd, *J* = 11.3, 10.3)
Indolic H			
1′	10.70 (br s)	10.71 (br s)	11.37 (br s)
2′	8.62 (d, *J* = 2.75)	8.62 (d, *J* = 2.7)	8.68 (s)
4′	8.37 (d, *J* = 8.6)	8.37 (d, *J* = 8.7)	8.32 (d, *J* = 8.5)
5′	7.17–7.23 (m) ^b^	7.20 (dd, *J* = 8.7, 1.8)	7.41 (dd, *J* = 8.5, 1.7)
7′	7.66 (d, *J* = 1.8)	7.66 (d, *J* = 1.8)	7.76 (d, *J* = 1.6)
1′′	10.34 (br s)	10.34 (br s)	10.42 (br s)
2′′	7.17–7.23 (m) ^b^	7.22 (dd, *J* = 2.5, <1)	7.45 (d, *J* = 2.1)
4′′	7.69 (d, *J* = 8.6)	7.69 (d, *J* = 8.5)	7.63 (d, *J* = 8.5)
5′′	7.17–7.23 (m) ^b^	7.20 (dd, *J* = 8.5, 1.7)	7.17 (dd, *J* = 8.5, 1.7)
7′′	7.63 (d, *J* = 1.7)	7.63 (d, *J* = 1.7)	7.65 (d, *J* = 1.6)

^a^ Recorded in acetone- *d*_6_ ; shift in ppm, coupling in Hz.

^b^ Double resonance experiments and COSY gave the same interpretation of these signals as shown for natural topsentin C (see the Supporting Information).

Acid chlorides **18a** and **18b** were obtained through acylation of the corresponding indoles by using oxalylchloride according to published methods. 6k The reaction first gave a mixture of amides **19** and **20** , which were further cyclized without isolation (Scheme 6 ). As noted earlier during the development of synthetic methods for hamacanthins, these amides can undergo reversible transformations under the cyclization conditions via intermediate **21** , and can form a mixture of isomeric bis(indolyl)dihydropyrazinones. 6b In this case, the cyclization was unidirectional because of the methyl group, so that **22a** and **22b** were isolated only. 21 These compounds were converted into target **17a** and **17b** by removing the protecting group. The structure of compound **17a** was established by X-ray crystallographic analysis (Figure 4 ).

**Figure 4:**
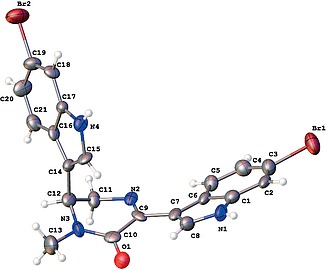
Molecular structure of **17a** presented as ADP ellipsoids at 50% probability

The spectral characteristics of bis(indolyl)dihydropyrazinone **17a** were consistent with those of natural topsentin C, 3 22 in contrast to imidazoline **5a** (Table 4 ). This data confirmed our hypothesis regarding the structure of the natural topsentin C.

Thus, we have developed a convenient preparative synthetic method to prepare (indol-3-yl)ethane-1,2-diamines and found that the natural topsentin C has the structure **17a** . The total synthesis of racemic **17a** was carried out in seven steps from 6-bromoindole **9c** in 55% overall yield.

## 

Starting reagents were either purchased from commercial sources and used without additional purification or were prepared according to reported procedures. ^1^ H and ^13^ C NMR spectra were acquired with 400, 500, or 600 MHz spectrometers at r.t. and referenced to the residual signals of the solvent (for ^1^ H and ^13^ C). The solvents for NMR samples were DMSO- *d*_6_ , CDCl _3_ , and acetone- *d*_6_ . Chemical shifts are reported in parts per million (δ, ppm). Coupling constants are reported in Hertz ( *J* , Hz). The peak patterns are indicated as: s, singlet; d, doublet; t, triplet; q, quadruplet; m, multiplet; dd, doublet of doublets; br s, broad singlet. Signal assignment was based on COSY, HSQC, HMBC and NOESY experiments. Infrared spectra were measured with an Infralum FT-801 FT/IR instrument. The wavelengths are reported in reciprocal centimeters (ν _max_ , cm ^–1^ ). Mass spectra were recorded with LCMS-8040 triple quadrupole liquid chromatograph mass-spectrometer from Shimadzu (ESI) and Kratos MS-30 mass spectrometer (EI, 70 eV). Elemental analysis was performed with an Euro Vector EA-3000 elemental analyzer. The X-ray data collection of **17a** was performed with a Bruker APEX-II CCD diffractometer at 120 K. Details of the X-ray structure determination are given in the Supporting Information. The progress of the reaction was monitored by TLC and the spots were visualized under UV light (254 or 365 nm). Column chromatography was performed using silica gel (230–400 mesh). Melting points were determined with a SMP-10 apparatus and are uncorrected. Solvents were distilled and dried according to standard procedures.

## *tert* -Butyl 3-[( *E* )-2-Nitrovinyl]-1 *H* -indole-1-carboxylate (9a) 23

SOCl _2_ (13.7 mL, 0.188 mol) at 0–5 °C was added dropwise to anhydrous DMF (14.6 mL, 0.188 mol) and the solution was stirred at r.t. for 30 min. The resulting mixture was degassed under vacuum and the residue was diluted with anhydrous DMF (26 mL) and treated with a solution of indole (20.0 g, 0.171 mol) in DMF (10 mL) dropwise at 0–5 °C over 10 min. The mixture was stirred at r.t. for 40 min, then treated with ice (100 g) and NaOH solution (30%) was added until pH ca. 8. The mixture was heated to reflux, and cooled. The resulting precipitate was filtered off, rinsed with H _2_ O (5×), dried in air, heated at reflux in EtOH (40 mL), cooled, filtered off again, and dried in air to afford 1 *H* -indole-3-carbaldehyde (22.5 g, 91%).

A solution of 1 *H* -indole-3-carbaldehyde (12.5 g, 0.086 mol) and NH _4_ OAc (6.6 g, 0.086 mmol) in CH _3_ NO _2_ (46 mL, 0.861 mol) was heated at reflux for 45 min, cooled to r.t., and diluted with H _2_ O (150 mL). The resulting precipitate was filtered off, washed with H _2_ O (5×), dried on air, heated at reflux in EtOH (60 mL), cooled, filtered off again and dried on air to afford 3-[( *E* )-2-nitrovinyl]-1 *H* -indole (11.3 g, 70%).

To a solution of 3-[( *E* )-2-nitrovinyl]-1 *H* -indole (5.6 g, 30 mmol) and DMAP (0.37 g, 3.0 mmol) in anhydrous THF (30 mL) was added dropwise a solution of Boc _2_ O (9.9 g, 45.4 mmol) in anhydrous THF (30 mL) at 0–5 °C over a period of 15 min. The reaction mixture was stirred at r.t. for 3 h, and concentrated in vacuo. The residue was dissolved in CH _2_ Cl _2_ (100 mL), washed with citric acid solution (10%, 30 mL), H _2_ O (30 mL), and concd NaCl solution (20 mL), then dried over anhydrous Na _2_ SO _4_ . The CH _2_ Cl _2_ was evaporated in vacuo and the residue was heated at reflux in MeOH (30 mL) and cooled. The resulting precipitate was filtered off, washed twice with cold hexane, and dried on air to afford *tert* -butyl 3-[( *E* )-2-nitrovinyl]-1 *H* -indole-1-carboxylate.

Yield: 7.6 g (55% from indole); yellow solid; mp 144–145 °C (MeOH).

^1^ H NMR (600 MHz, DMSO- *d*_6_ ): δ = 8.55 (s, 1 H), 8.36 (d, *J* = 13.7 Hz, 1 H, C *H* =CHNO _2_ ), 8.19 (d, *J* = 13.7 Hz, 1 H, CH=C *H* NO _2_ ), 8.11 (d, *J* = 8.1 Hz, 1 H), 8.02 (d, *J* = 7.1 Hz, 1 H), 7.40–7.46 (m, 1 H), 7.33–7.39 (m, 1 H), 1.64 (s, 9 H, C(CH _3_ ) _3_ ).

^13^ C NMR (150 MHz, DMSO- *d*_6_ ): δ = 148.79, 136.50, 136.06, 134.27, 132.37, 127.15, 126.14, 124.54, 121.40, 115.67, 112.76, 85.72, 28.08 (3С).

## *tert* -Butyl 5-Bromo-3-[( *E* )-2-nitrovinyl]-1 *H* -indole-1-carboxylate (9b); Typical Procedure

5-Bromoindole (1.35 g, 6.9 mmol) was formylated as described for 1 *H* -indole-3-carbaldehyde to produce 6-bromo-1 *H* -indole-3-carbaldehyde (1.50 g, 97%) with the exception that the obtained product was not purified by refluxing in EtOH.

5-Bromo-1 *H* -indole-3-carbaldehyde (1.50 g, 6.7 mmol) and NH _4_ OAc (0.52 g, 6.7 mmol) were heated at reflux in CH _3_ NO _2_ (18 mL, 335 mmol) for 45 min, cooled to r.t., and treated with H _2_ O (70 mL). The product was extracted with EtOAc (70 mL), washed with H _2_ O (5 × 50 mL) and NaCl solution (20 mL), and dried over anhydrous Na _2_ SO _4_ . The solvent was removed in vacuo to afford 6-bromo-3-[( *E* )-2-nitrovinyl]-1 *H* -indole (1.72 g, 96%, brownish crystals), which was dried in vacuo and used without further purification.

A suspension of 5-bromo-3-[( *E* )-2-nitrovinyl]-1 *H* -indole (1.72 g, 6.4 mmol) and DMAP (0.08 g, 0.64 mmol) in anhydrous THF (7 mL) was treated with a solution of Boc _2_ O (2.1 g, 9.7 mmol) in anhydrous THF (7 mL) dropwise at 0–5 °C over a period of 15 min, stirred at r.t. for 3 h, and concentrated in vacuo. The residue was dissolved in CH _2_ Cl _2_ (50 mL), washed with citric acid solution (10%, 20 mL), H _2_ O (20 mL), and concd NaCl solution (15 mL), and dried over anhydrous Na _2_ SO _4_ . The CH _2_ Cl _2_ was evaporated in vacuo and the residue was purified by chromatography on a column of silica gel (EtOAc–hexane, 6:1) to provide *tert* -butyl 5-bromo-3-[( *E* )-2-nitrovinyl]-1 *H* -indole-1-carboxylate.

Yield: 2.11 g (83% from 5-bromoindole); pale-yellow solid; mp 156–157 °C (MeOH); *R _f_* = 0.55 (EtOAc–hexane, 1:8).

IR (film): 3130, 2983, 2300 w, 1741 s (CO), 1639, 1509, 1452, 1368, 1344, 1240, 1153, 1107, 956, 853, 803, 764, 649, 613, 579 cm ^–1^ .

^1^ H NMR (400 MHz, DMSO- *d*_6_ ): δ = 8.57 (s, 1 H), 8.33 (d, *J* = 13.7 Hz, 1 H, C *H* =CHNO _2_ ), 8.27 (d, *J* = 1.9 Hz, 1 H), 8.26 (d, *J* = 13.7 Hz, 1 H, CH=C *H* NO _2_ ), 8.02 (d, *J* = 8.8 Hz, 1 H), 7.56 (dd, *J* = 8.8, 1.9 Hz, 1 H), 1.65 (s, 9 H, C(CH _3_ ) _3_ ).

^13^ C NMR (100 MHz, DMSO- *d*_6_ ): δ = 147.89, 136.57, 134.27, 133.94, 130.89, 128.52, 128.16, 123.08, 116.83, 116.76, 111.47, 85.53, 27.48 (3С).

MS (EI, 70 eV): *m* / *z* (%) = 368/366 (4) [M] ^+^ , 312/310 (7) [M – C _4_ H _8_ ] ^+^ , 268/266 (31) [M – C _4_ H _8_ – CO _2_ ] ^+^ , 219 (21), 140 (30), 57 (100).

Anal. Calcd for C _15_ H _15_ BrN _2_ O _4_ : C, 49.06; H, 4.12; N, 7.63. Found: C, 49.19; H, 4.15; N, 7.55.

## *tert* -Butyl 6-Bromo-3-[( *E* )-2-nitrovinyl]-1 *H* -indole-1-carboxylate (9c)

Yield: 2.15 g (85% from 6-bromoindole); pale-yellow solid; mp 124–126 °C (MeOH; *R _f_* = 0.62 (EtOAc–hexane, 1:8).

IR (film): 3129, 2985, 2300 w, 1740 s (CO), 1635, 1505, 1427, 1341, 1299, 1238, 1144, 1106, 965, 842, 808, 764, 726, 650, 591 cm ^–1^ .

^1^ H NMR (600 MHz, CDCl _3_ ): δ = 8.43 (s, 1 H), 8.12 (d, *J* = 13.6 Hz, 1 H, C *H* =CHNO _2_ ), 7.98 (s, 1 H), 7.71 (d, *J* = 13.6 Hz, 1 H, CH=C *H* NO _2_ ), 7.54 (d, *J* = 8.3 Hz, 1 H), 7.48 (dd, *J* = 8.3, 1.7 Hz, 1 H), 1.71 (s, 9 H, C(CH _3_ ) _3_ ).

^13^ C NMR (150 MHz, CDCl _3_ ): δ = 148.29, 137.09, 136.22, 132.14, 130.97, 127.58, 125.79, 121.20, 119.87, 119.27, 112.37, 86.27, 28.15 (3С).

MS (EI, 70 eV): *m* / *z* (%) = 368/366 (7) [M] ^+^ , 312/310 (11) [M – C _4_ H _8_ ] ^+^ , 268/266 (40) [M – C _4_ H _8_ – CO _2_ ] ^+^ , 221 (18), 140 (21), 57 (100).

Anal. Calcd for C _15_ H _15_ BrN _2_ O _4_ : C, 49.06; H, 4.12; N, 7.63. Found: C, 49.24; H, 4.19; N, 7.60.

## *tert* -Butyl 5-Methoxy-3-[( *E* )-2-nitrovinyl]-1 *H* -indole-1-carboxylate (9d)

Yield: 1.73 g (79% from 5-methoxyindole); yellow solid; mp 153–154 °C (MeOH); *R _f_* = 0.52 (EtOAc–hexane, 1:8).

IR (film): 3139, 2982, 2945, 2296 w, 1732 s (CO), 1633, 1544, 1500, 1374, 1325, 1251, 1161, 1097, 1024, 969, 834, 800, 765, 640, 592 cm ^–1^ .

^1^ H NMR (600 MHz, DMSO- *d*_6_ ): δ = 8.52 (s, 1 H), 8.37 (d, *J* = 13.6 Hz, 1 H, C *H* =CHNO _2_ ), 8.24 (d, *J* = 13.6 Hz, 1 H, CH=C *H* NO _2_ ), 7.98 (d, *J* = 9.0 Hz, 1 H), 7.45 (d, *J* = 2.6 Hz, 1 H), 7.01 (dd, *J* = 9.0, 2.6 Hz, 1 H), 3.86 (s, 3 H, OCH _3_ ), 1.64 (s, 9 H, C(CH _3_ ) _3_ ).

^13^ C NMR (150 MHz, DMSO- *d*_6_ ): δ = 156.54, 148.24, 135.93, 133.46, 131.80, 129.90, 127.84, 115.87, 114.40, 112.13, 103.33, 85.04, 55.73, 27.56 (3С).

MS (EI, 70 eV): *m* / *z* (%) = 319 (8), 318 (41) [M] ^+^ , 263 (17), 262 (81) [M – C _4_ H _8_ ] ^+^ , 219 (23), 218 (28) [M – C _4_ H _8_ – CO _2_ ] ^+^ , 186 (31), 175 (69), 156 (29), 57 (100).

Anal. Calcd for C _16_ H _18_ N _2_ O _5_ : C, 60.37; H, 5.70; N, 8.80. Found: C, 60.28; H, 5.76; N, 8.79.

## *tert* -Butyl 6-Chloro-3-[( *E* )-2-nitrovinyl]-1 *H* -indole-1-carboxylate (9e)

Yield: 1.71 g (77% from 6-chloroindole); mp 130–132 °C (MeOH); *R _f_* = 0.62 (EtOAc–hexane, 1:8).

IR (film): 3131, 2983, 2294 w, 1741 s (CO), 1634, 1508, 1431, 1340, 1299, 1240, 1145, 1103, 968, 845, 810, 762, 732, 660, 599 cm ^–1^ .

^1^ H NMR (400 MHz, DMSO- *d*_6_ ): δ = 8.54 (s, 1 H), 8.32 (d, *J* = 13.7 Hz, 1 H, C *H* =CHNO _2_ ), 8.17 (d, *J* = 13.7 Hz, 1 H, CH=C *H* NO _2_ ), 8.08 (d, *J* = 1.7 Hz, 1 H), 8.04 (d, *J* = 8.6 Hz, 1 H), 7.36 (dd, *J* = 8.6, 1.7 Hz, 1 H), 1.65 (s, 9 H, C(CH _3_ ) _3_ ).

^13^ C NMR (100 MHz, DMSO- *d*_6_ ): δ = 147.85, 136.40, 135.86, 133.93, 131.10, 130.14, 125.40, 123.99, 122.14, 114.88, 111.96, 85.68, 27.45 (3С).

MS (EI, 70 eV): *m* / *z* (%) = 324 (14)/322 (42) [M] ^+^ , 268 (9)/266 (27) [M – C _4_ H _8_ ] ^+^ , 224 (16)/222 (55) [M – C _4_ H _8_ – CO _2_ ] ^+^ , 179 (26), 174 (26), 113 (97), 57 (100).

Anal. Calcd for C _15_ H _15_ ClN _2_ O _4_ : C, 55.82; H, 4.68; N, 8.68. Found: C, 55.70; H, 4.73; N, 8.63.

## *tert* -Butyl 3-{1-[(Benzyloxy)amino]-2-nitroethyl}-5-bromo-1 *H* -indole-1-carboxylate (11)

To a solution of 5-bromo-3-[( *E* )-2-nitrovinyl]-1 *H* -indole (0.55 g, 1.5 mmol) in anhydrous THF (3 mL), *O* -benzylhydroxylamine (0.19 g, 1.6 mmol) was added. The mixture was stirred at r.t. for 1 h and allowed to stand overnight. After removing the volatile components in vacuo, the adduct **11** was obtained.

Yield: 0.74 g (ca. 100%); viscous amber oil; *R _f_* = 0.53 (EtOAc–hexane, 1:8).

IR (film): 3253, 2980, 2931, 1738 s (CO), 1554, 1451, 1373, 1278, 1154, 1057, 843, 805, 750, 699, 609 cm ^–1^ .

^1^ H NMR (400 MHz, CDCl _3_ ): δ = 8.03 (d, *J* = 8.9 Hz, 1 H), 7.75 (d, *J* = 1.9 Hz, 1 H), 7.58 (s, 1 H), 7.44 (dd, *J* = 8.9, 1.9 Hz, 1 H), 7.30–7.40 (m, 5 H, Ph), 5.84 (br s, 1 H, NH), 4.93–5.05 (m, 2 H, C *H* CH _a_ H _b_ + CHCH _a_ *H*_b_ ), 4.67–4.74 (m, 2 H, C *H*_2_ Ph), 4.65 (dd, *J* = 11.2, 4.2 Hz, 1 H, CHC *H*_a_ H _b_ ), 1.67 (s, 9 H, OC(CH _3_ ) _3_ ).

^13^ C NMR (125 MHz, CDCl _3_ ): δ = 148.88, 136.92, 134.18, 130.03, 128.75, 128.48, 128.20, 127.94, 125.45, 122.16, 116.90, 116.36, 114.49, 84.78, 77.08, 76.40, 55.70, 28.09.

MS (ESI): *m* / *z* = 492/490 [M + H] ^+^ .

Anal. Calcd for C _22_ H _24_ BrN _3_ O _5_ : C, 53.89; H, 4.93; N, 8.57. Found: C, 54.01; H, 5.00; N, 8.58.

## Addition of *O* -Pivaloylhydroxylamines 8a,b to Nitrovinylindoles 9a–f; General Procedure

The hydrochloride salt of the corresponding *O* -pivaloylhydroxylamine (6 mmol) was dissolved in CH _2_ Cl _2_ (20 mL) and carefully shaken with saturated aqueous NaHCO _3_ solution, then the organic phase was washed with concd NaCl solution and dried over anhydrous Na _2_ SO _4_ . The solvent was removed in vacuo at 30 °C and the resulting *O* -pivaloylhydroxylamine (ca. 5.2 mmol) was carefully mixed with nitrovinylindole (3.5 mmol) in a round-bottom vial and allowed to stand overnight at r.t. The reaction mixture was triturated with hexane (7 mL) and the resulting crystals of adducts **7a** – **h** were filtered, washed with cold hexane (2 × 4 mL), and dried in vacuo.

## *tert* -Butyl 3-{1-[(Pivaloyloxy)amino]-2-nitroethyl}-1 *H* -indole-1-carboxylate (7a)

Yield: 1.33 g (94%); light-brown solid; mp 109–111 °C (hexane); *R _f_* = 0.46 (EtOAc–hexane, 1:8).

IR (film): 3229, 2977, 2936, 1746 s (CO), 1727 s (CO), 1555, 1452, 1371, 1278, 1235, 1154, 1085, 822, 762, 661, 594 cm ^–1^ .

^1^ H NMR (400 MHz, CDCl _3_ ): δ = 8.19 (d, *J* = 8.1 Hz, 1 H), 7.89 (d, *J* = 3.5 Hz, 1 H, NH), 7.70 (d, *J* = 7.7 Hz, 1 H), 7.67 (s, 1 H), 7.39 (dd, *J* = 8.1, 7.3 Hz, 1 H), 7.31 (dd, *J* = 7.7, 7.3 Hz, 1 H), 5.14–5.26 (m, 1 H, C *H* CH _a_ H _b_ ), 5.01 (dd, *J* = 12.9, 8.0 Hz, 1 H, CHCH _a_ *H*_b_ ), 4.75 (dd, *J* = 12.9, 4.5 Hz, 1 H, CHC *H*_a_ H _b_ ), 1.68 (s, 9 H, OC(CH _3_ ) _3_ ), 1.24 (s, 9 H, C(CH _3_ ) _3_ ).

^13^ C NMR (100 MHz, CDCl _3_ ): δ = 178.21, 149.15, 135.48, 127.82, 125.36, 124.60, 123.23, 118.99, 115.61, 113.38, 84.51, 78.04, 56.25, 38.44, 28.15 (3C), 26.90 (3С).

MS (ESI): *m* / *z* = 406 [M + H] ^+^ , 345 [M – CH _3_ NO _2_ + H] ^+^ , 304 [M – (CH _3_ ) _3_ CO _2_ H + H] ^+^ .

Anal. Calcd for C _20_ H _27_ N _3_ O _6_ : C, 59.25; H, 6.71; N, 10.36. Found: C, 59.31; H, 6.73; N, 10.11.

## *tert* -Butyl 3-{1-[(Pivaloyloxy)amino]-2-nitroethyl}-5-bromo-1 *H* -indole-1-carboxylate (7b)

Yield: 1.62 g (96%); white solid; mp 108–109 °C (hexane); *R _f_* = 0.39 (EtOAc–hexane, 1:8).

IR (film): 3260, 2974, 2935, 1728 s (CO), 1557, 1453, 1378, 1276, 1157, 1092, 1057, 872, 805, 778, 662, 604 cm ^–1^ .

^1^ H NMR (400 MHz, CDCl _3_ ): δ = 8.07 (d, *J* = 8.8 Hz, 1 H), 7.84 (d, *J* = 1.8 Hz, 1 H), 7.66 (s, 1 H), 7.47 (dd, *J* = 8.8, 1.8 Hz, 1 H), 7.89 (br s, 1 H, NH), 5.11 (dd, *J* = 7.7, 4.8 Hz, 1 H, C *H* CH _a_ H _b_ ), 4.99 (dd, *J* = 12.8, 7.7 Hz, 1 H, CHCH _a_ *H*_b_ ), 4.72 (dd, *J* = 12.8, 4.8 Hz, 1 H, CHC *H*_a_ H _b_ ), 1.67 (s, 9 H, OC(CH _3_ ) _3_ ), 1.24 (s, 9 H, C(O)C(CH _3_ ) _3_ ).

^13^ C NMR (100 MHz, CDCl _3_ ): δ = 178.26, 148.84, 134.37, 129.59, 128.39, 125.86, 121.98, 117.14, 116.75, 112.91, 85.13, 77.87, 56.20, 38.54, 28.19 (3C), 27.00 (3C).

MS (ESI): *m* / *z* = 486/484 [M + H] ^+^ , 425/423 [M – CH _3_ NO _2_ + H] ^+^ , 384/382 [M – (CH _3_ ) _3_ CO _2_ H + H] ^+^ .

Anal. Calcd for C _20_ H _26_ BrN _3_ O _6_ : C, 49.60; H, 5.41; N, 8.68. Found: C, 49.71; H, 5.50; N, 8.53.

## *tert* -Butyl 3-{1-[(Pivaloyloxy)(methyl)amino]-2-nitroethyl}-1 *H* -indole-1-carboxylate (7c)

Yield: 1.42 g (97%); light-beige solid; mp 113 °C (hexane); *R _f_* = 0.47 (EtOAc–hexane, 1:8).

IR (film): 2977, 2931, 1754 s (CO), 1737 s (CO), 1557, 1452, 1378, 1248, 1153, 1103, 1075, 1026, 845, 750, 702, 659 cm ^–1^ .

^1^ H NMR (400 MHz, CDCl _3_ ): δ = 8.18 (d, *J* = 7.7 Hz, 1 H), 7.81 (d, *J* = 7.7 Hz, 1 H), 7.69 (s, 1 H), 7.38 (dd, *J* = 8.1, 7.3 Hz, 1 H), 7.31 (dd, *J* = 7.8, 7.7 Hz, 1 H), 5.08 (dd, *J* = 7.4, 5.6 Hz, 1 H, C *H* CH _a_ H _b_ ), 5.00 (dd, *J* = 12.7, 7.4 Hz, 1 H, CHCH _a_ *H*_b_ ), 4.65 (dd, *J* = 12.7, 5.6 Hz, 1 H, CHC *H*_a_ H _b_ ), 2.68 (s, 3 H, NCH _3_ ), 1.69 (s, 9 H, OC(CH _3_ ) _3_ ), 1.26 (s, 9 H, C(O)C(CH _3_ ) _3_ ).

^13^ C NMR (100 MHz, CDCl _3_ ): δ = 175.96, 149.16, 135.30, 128.65, 125.22, 124.99, 123.24, 119.51, 115.36, 113.78, 84.38, 77.17, 62.04, 43.84, 38.67, 28.10 (3C), 27.05 (3C).

MS (ESI): *m* / *z* = 420 [M + H] ^+^ , 318 [M – (CH _3_ ) _3_ CO _2_ H + H] ^+^ .

Anal. Calcd for C _21_ H _29_ N _3_ O _6_ : C, 60.13; H, 6.97; N, 10.02. Found: C, 60.27; H, 7.03; N, 10.00.

## *tert* -Butyl 3-{1-[(Pivaloyloxy)(methyl)amino]-2-nitroethyl}-5-bromo-1 *H* -indole-1-carboxylate (7d)

Yield: 1.55 g (89%); amorphous amber solid; *R _f_* = 0.53 (EtOAc–hexane, 1:8).

IR (film): 2977, 2933, 1744 s (CO), 1602, 1559, 1451, 1370, 1276, 1153, 1092, 1057, 842, 807, 767, 679, 609 cm ^–1^ .

^1^ H NMR (600 MHz, CDCl _3_ ): δ = 8.07 (br s, 1 H), 7.93 (d, *J* = 1.7 Hz, 1 H), 7.68 (s, 1 H), 7.47 (dd, *J* = 9.0, 1.7 Hz, 1 H), 4.95–5.03 (m, 2 H, C *H* CH _a_ H _b_ + CHCH _a_ *H*_b_ ), 4.63 (dd, *J* = 11.6, 5.0 Hz, 1 H, CHC *H*_a_ H _b_ ), 2.67 (s, 3 H, NCH _3_ ), 1.68 (s, 9 H, OC(CH _3_ ) _3_ ), 1.26 (s, 9 H, C(O)C(CH _3_ ) _3_ ).

^13^ C NMR (100 MHz, CDCl _3_ ): δ = 175.79, 148.76, 134.11, 130.37, 128.20, 126.17, 122.20, 116.87, 116.71, 113.16, 84.94, 77.00, 61.86, 43.83, 38.66, 28.08 (3C), 27.07 (3C).

MS (ESI): *m* / *z* = 500/498 [M + H] ^+^ , 398/396 [M – (CH _3_ ) _3_ CO _2_ H + H] ^+^ .

Anal. Calcd for C _21_ H _28_ BrN _3_ O _6_ : C, 50.61; H, 5.66; N, 8.43. Found: C, 50.90; H, 5.81; N, 8.25.

## *tert* -Butyl 3-{1-[(Pivaloyloxy)(methyl)amino]-2-nitroethyl}-6-bromo-1 *H* -indole-1-carboxylate (7e)

Yield: 1.70 g (98%); light-beige solid; mp 115–116 °C (hexane); *R _f_* = 0.39 (EtOAc–hexane, 1:8).

IR (film): 2976, 2933, 1762 s (CO), 1727 s (CO), 1561, 1433, 1370, 1255, 1152, 1097, 865, 818, 772, 679, 590 cm ^–1^ .

^1^ H NMR (600 MHz, CDCl _3_ ): δ = 8.40 (s, 1 H), 7.70 (d, *J* = 8.3 Hz, 1 H), 7.64 (s, 1 H), 7.42 (dd, *J* = 8.3, 1.7 Hz, 1 H), 5.02 (dd, *J* = 7.4, 5.8 Hz, 1 H, C *H* CH _a_ H _b_ ), 4.98 (dd, *J* = 12.4, 7.4 Hz, 1 H, CHCH _a_ *H*_b_ ), 4.63 (dd, *J* = 12.7, 5.8 Hz, 1 H, CHC *H*_a_ H _b_ ), 2.66 (s, 3 H, NCH _3_ ), 1.69 (s, 9 H, OC(CH _3_ ) _3_ ), 1.26 (s, 9 H, C(O)C(CH _3_ ) _3_ ).

^13^ C NMR (100 MHz, CDCl _3_ ): δ = 175.92, 148.72, 136.02, 127.39, 126.53, 125.29, 120.84, 119.09, 118.61, 113.91, 85.01, 77.00, 62.02, 43.70, 38.64, 28.03 (3C), 27.02 (3C).

MS (ESI): *m* / *z* = 522/520 [M + Na] ^+^ .

Anal. Calcd for C _21_ H _28_ BrN _3_ O _6_ : C, 50.61; H, 5.66; N, 8.43. Found: C, 50.73; H, 5.72; N, 8.42.

## *tert* -Butyl 3-{1-[(Pivaloyloxy)(methyl)amino]-2-nitroethyl}-5-methoxy­-1 *H* -indole-1-carboxylate (7f)

Yield: 1.43 (91%); yellow solid; mp 96 °C (hexane); *R _f_* = 0.33 (EtOAc–hexane, 1:8).

IR (film): 2971, 2935, 1755 s (CO), 1731 s (CO), 1557, 1480, 1382, 1286, 1157, 1098, 1070, 849, 807, 767, 677, 626 cm ^–1^ .

^1^ H NMR (600 MHz, CDCl _3_ ): δ = 8.04 (br s, 1 H), 7.64 (s, 1 H), 7.28 (d, *J* = 1.7 Hz, 1 H), 6.97 (dd, *J* = 9.0, 1.7 Hz, 1 H), 5.02 (dd, *J* = 7.4, 5.0 Hz, 1 H, C *H* CH _a_ H _b_ ), 4.98 (dd, *J* = 12.4, 7.4 Hz, 1 H, CHCH _a_ *H*_b_ ), 4.63 (dd, *J* = 12.4, 5.0 Hz, 1 H, CHC *H*_a_ H _b_ ), 3.90 (s, 3 H, OCH _3_ ), 2.68 (s, 3 H, NCH _3_ ), 1.67 (s, 9 H, OC(CH _3_ ) _3_ ), 1.25 (s, 9 H, C(O)C(CH _3_ ) _3_ ).

^13^ C NMR (100 MHz, CDCl _3_ ): δ = 175.89, 156.27, 149.14, 130.00, 129.53, 125.39, 116.13, 114.07, 113.70, 102.11, 84.21, 77.00, 62.13, 55.78, 43.71, 38.66, 28.12 (3C), 27.06 (3C).

MS (ESI): *m* / *z* = 450 [M + H] ^+^ , 348 [M – (CH _3_ ) _3_ CO _2_ H + H] ^+^ .

Anal. Calcd for C _22_ H _31_ N _3_ O _7_ : C, 58.78; H, 6.95; N, 9.35. Found: C, 58.89; H, 7.00; N, 9.23.

## *tert* -Butyl 3-{1-[(Pivaloyloxy)(methyl)amino]-2-nitroethyl}-6-chloro-1 *H* -indole-1-carboxylate (7g)

Yield: 1.54 (97%); light-brown solid; mp 108–109 °C (hexane); *R _f_* = 0.39 (EtOAc–hexane, 1:8).

IR (film): 2976, 2906, 1754 s (CO), 1744 s (CO), 1562, 1439, 1368, 1256, 1157, 1100, 866, 817, 761, 661, 600 cm ^–1^ .

^1^ H NMR (400 MHz, CDCl _3_ ): δ = 8.21 (s, 1 H), 7.75 (d, *J* = 8.5 Hz, 1 H), 7.65 (s, 1 H), 7.27 (dd, *J* = 8.5, 1.7 Hz, 1 H), 4.92–5.02 (m, 2 H, C *H* CH _a_ H _b_ + CHCH _a_ *H*_b_ ), 4.63 (dd, *J* = 12.1, 5.3 Hz, 1 H, CHC *H*_a_ H _b_ ), 2.66 (s, 3 H, NCH _3_ ), 1.68 (s, 9 H, OC(CH _3_ ) _3_ ), 1.25 (s, 9 H, C(O)C(CH _3_ ) _3_ ).

^13^ C NMR (100 MHz, CDCl _3_ ): δ = 175.91, 148.75, 135.75, 131.35, 127.04, 125.38, 123.86, 120.53, 115.70, 113.92, 84.98, 77.00, 62.09, 43.68, 38.65, 28.04 (3C), 27.02 (3C).

MS (ESI): *m* / *z* = 456/454 [M + H] ^+^ , 354/352 [M – (CH _3_ ) _3_ CO _2_ H + H] ^+^ .

Anal. Calcd for C _21_ H _28_ ClN _3_ O _6_ : C, 55.57; H, 6.22; N, 9.26. Found: C, 55.69; H, 6.28; N, 9.21.

## 1-Acetyl-3-{1-[(pivaloyloxy)(methyl)amino]-2-nitroethyl}-1 *H* -indole (7h)

Yield: 1.11 g (88%); light-orange solid; mp 84–85 °C (hexane); *R _f_* = 0.15 (EtOAc–hexane, 1:8).

IR (film): 2973, 2937, 2910, 2874, 1753 s (CO), 1715 s (CO), 1553, 1451, 1386, 1313, 1225, 1125, 1077, 1023, 939, 755, 672, 641, 564 cm ^–1^ .

^1^ H NMR (600 MHz, CDCl _3_ ): δ = 8.44 (br d, *J* = 8.3 Hz, 1 H), 7.83 (d, *J* = 7.4 Hz, 1 H), 7.55 (s, 1 H), 7.38–7.43 (m, 1 H), 7.32–7.37 (m, 1 H), 5.07 (dd, *J* = 7.4, 5.8 Hz, 1 H, C *H* CH _a_ H _b_ ), 4.98 (dd, *J* = 12.8, 7.4 Hz, 1 H, CHCH _a_ *H*_b_ ), 4.66 (dd, *J* = 12.8, 5.8 Hz, 1 H, CHC *H*_a_ H _b_ ), 2.70 (s, 3 H, C(O)CH _3_ or NCH _3_ ), 2.66 (s, 3 H, C(O)CH _3_ or NCH _3_ ), 1.25 (s, 9 H, C(O)C(CH _3_ ) _3_ ).

^13^ C NMR (100 MHz, CDCl _3_ ): δ = 175.98, 168.27, 135.76, 128.36, 126.16, 124.28, 124.22, 119.58, 116.69, 116.11, 77.14, 62.39, 44.09, 38.68, 27.00 (3C), 23.92.

MS (ESI): *m* / *z* = 362 [M + H] ^+^ , 260 [M – (CH _3_ ) _3_ CO _2_ H + H] ^+^ .

Anal. Calcd for C _18_ H _23_ N _3_ O _5_ : C, 59.82; H, 6.41; N, 11.63. Found: C, 60.00; H, 6.48; N, 11.58.

## Catalytic Hydrogenation of Adduct 11

To a solution of adduct **11** (0.54 g, 1.1 mmol) in MeOH (10 mL) was added AcOH (9.5 mL, 1.165 mol) and 5% Pd on charcoal (0.05 g) and the mixture was purged with hydrogen. The mixture was vigorously stirred under a hydrogen atmosphere (1 atm) for 18 h, filtered, and concentrated in vacuo. The residue was dissolved in CH _2_ Cl _2_ (50 mL), treated with cold NaOH solution (10%, 40 mL) and concd NaCl solutions (20 mL), and dried over anhydrous Na _2_ SO _4_ . The solvent was removed in vacuo and the residue was purified by chromatography on a column of silica gel (CHCl _3_ –MeOH–NH _3(aq)_ , 100:10:0.2) to afford diamine **7b** (0.206 g, 68 %). Its characteristics correspond to the sample obtained from adduct **9a** (see below).

## Reduction of Adduct 11 with Zn Dust

To a solution of adduct **11** (0.54 g, 1.1 mmol) in MeOH (10 mL) was added AcOH (9.5 mL, 1.165 mol) and Zn dust (1.44 g, 22 mmol). The mixture was vigorously stirred at r.t. for 2 h, and then another portion of Zn dust (1.43 g, 22 mmol) was added. The resulting mixture was vigorously stirred further for 12 h, filtered, and concentrated in vacuo. The residue was dissolved in CH _2_ Cl _2_ (100 mL), treated with chipped ice (10 g), and carefully shaken with cold NaOH solution (10%, 90 mL). The organic layer was separated and washed with cold NaOH (10%, 30 mL) and concd NaCl solutions (20 mL), and dried over anhydrous Na _2_ SO _4_ . The solvent was removed in vacuo and the residue was purified by chromatography on a column of silica gel with gradient of MeOH in CHCl _3_ to afford compound **12** .

Yield: 0.27 g (53%); pale-yellow oil.

IR (film): 3378 br, 2978, 2931, 2866, 1736 s, (CO), 1603, 1451, 1371, 1256, 1155, 1054, 842, 803, 750, 698, 611 cm ^–1^ .

^1^ H NMR (600 MHz, CDCl _3_ ): δ = 8.03 (m, 1 H), 7.81 (d, *J* = 1.8 Hz, 1 H), 7.55 (s, 1 H), 7.41 (dd, *J* = 8.8, 1.8 Hz, 1 H), 7.28–7.36 (m, 5 H, Ph), 6.02 (br s, 1 H, NH), 4.64–4.70 (m, 2 H, C *H*_2_ Ph), 4.19–4.24 (m, 1 H, C *H* CH _2_ ), 3.07–3.13 (m, 2 H, CHC *H*_2_ ), 1.67 (s, 9 H, OC(CH _3_ ) _3_ ), 1.47 (br s, 2 H, NH _2_ ).

^13^ C NMR (150 MHz, CDCl _3_ ): δ = 149.20, 137.62, 134.28, 131.10, 128.54, 128.31, 127.85, 127.33, 124.77, 122.60, 118.24, 116.70, 115.90, 84.18, 76.73, 59.96, 43.26, 28.12 (3C).

MS (ESI): *m* / *z* = 462/460 [M + H] ^+^ .

Anal. Calcd for C _22_ H _26_ BrN _3_ O _3_ : C, 57.40; H, 5.69; N, 9.13. Found: C, 57.37; H, 5.61; N, 9.04.

After the chromatography, diamine **7b** (5.8 mg, 15%) was also isolated. The analytical data was in accordance with the characteristics of the sample obtained from adduct **9b** (see below).

## Synthesis of (Indol-3-yl)ethane-1,2-diamines 6; General Procedure A

A solution of adduct **7** (2.2 mmol) in EtOAc (8.3 mL) was added to a cooled (–10 °C) mixture of MeOH (16.5 mL) and HBr (40%, 5.5 mL, 37.8 mmol) under vigorous stirring and treated with Zn dust (2.2 g, 33 mmol). The reaction mixture was allowed to warm to 0–5 °C (1 h), stirred at that temperature for 1 h, and filtered. The precipitate was filtered off and rinsed with a small amount of MeOH. The filtrate was diluted with CH _2_ Cl _2_ (100 mL), treated with crushed ice (10 g), and carefully shaken with cold NaOH solution (10%, 90 mL). The organic layer was separated and the aqueous layer was washed with CH _2_ Cl _2_ (20 mL). The combined organic extracts were washed with cold NaOH (10%, 30 mL) and concd NaCl solutions (20 mL), and dried over anhydrous Na _2_ SO _4_ . The solvent was removed in vacuo to afford target diamine **6** as a yellowish oil. The product was pure enough for further syntheses. Chromatographic purification on a column with silica gel (CHCl _3_ –MeOH–NH _3(aq)_ , 100:10:0.2) was performed, if necessary.

## Synthesis of (Indol-3-yl)ethane-1,2-diamines 6; General Procedure B

A solution of adduct **7** (2.2 mmol) in EtOAc (13 mL) was added to a cooled (–10 °C) mixture of MeOH (26 mL) and HBr (40%, 13 mL, 89.0 mmol) under vigorous stirring and treated with Zn dust (1.44 g, 22 mmol). The mixture was allowed to warm to 0 °C (1 h), then another portion of Zn dust (1.44 g, 22 mmol) was added. The reaction mixture was stirred further at 0–5 °C for 5 h, and filtered. The precipitate was rinsed with a small amount of MeOH and the filtrate was diluted with CH _2_ Cl _2_ (100 mL), treated with crushed ice (10 g), and shaken carefully with cold NaOH solution (10%, 120 mL). General Procedure A was then followed.

## 1-[(1- *tert* -Butoxycarbonyl)-1 *H* -indol-3-yl]ethane-1,2-diamine (6a)

Obtained by following General Procedure A.

Yield: 0.51 g (84%); pale-yellow viscous oil; *R _f_* = 0.30 (CHCl _3_ –MeOH–NH _3(aq)_ , 10:1:0.02).

IR (film): 3365 br, 2978, 2932, 2868, 1729 s (CO), 1606, 1555, 1453, 1375, 1256, 1158, 1079, 856, 747, 591 cm ^–1^ .

^1^ H NMR (400 MHz, CDCl _3_ ): δ = 8.16 (d, *J* = 8.2 Hz, 1 H, 7-H), 7.61 (d, *J* = 7.8 Hz, 1 H, 4-H), 7.54 (s, 1 H, 2-H), 7.32 (dd, *J* = 8.2, 7.1 Hz, 1 H, 6-H), 7.23 (dd, *J* = 7.8, 7.1 Hz, 1 H, 5-H), 4.23 (dd *J* = 6.6, 4.5 Hz, 1 H, C *H* CH _a_ H _b_ ), 3.10 (dd, *J* = 12.7, 4.5 Hz, 1 H, CHCH _a_ *H*_b_ ), 2.94 (dd, *J* = 12.7, 6.6 Hz, 1 H, CHC *H*_a_ H _b_ ), 1.67 (s, 9 H, OC(CH _3_ ) _3_ ), 1.58 (br s, 4 H, 2NH _2_ ).

^13^ C NMR (100 MHz, CDCl _3_ ): δ = 149.59, 135.74, 128.83, 124.43, 123.34, 122.39, 122.31, 119.17, 115.34, 83.57, 50.51, 47.94, 28.09.

MS (ESI): *m* / *z* = 276 [M + H] ^+^ , 259 [M – NH _3_ +H] ^+^ .

Anal. Calcd for C _15_ H _21_ N _3_ O _2_ : C, 65.43; H, 7.69; N, 15.26. Found: C, 65.33; H, 7.73; N, 15.15.

## 1-[5-Bromo-(1- *tert* -Butoxycarbonyl)-1 *H* -indol-3-yl]ethane-1,2-diamine (6b)

Obtained by following General Procedure A.

Yield: 0.75 g (95%); pale-yellow viscous oil; *R _f_* = 0.35 (CHCl _3_ –MeOH–NH _3(aq)_ , 10:1:0.02).

IR (film): 3368 br, 2979, 2933, 2867, 1733 s (CO), 1601, 1450, 1372, 1276, 1255, 1157, 1055, 842, 801, 765, 636 cm ^–1^ .

^1^ H NMR (400 MHz, CDCl _3_ ): δ = 8.02 (d, *J* = 8.8 Hz, 1 H, 7-H), 7.76 (d, *J* = 2.0 Hz, 1 H, 4-H), 7.54 (s, 1 H, 2-H), 7.39 (dd, *J* = 8.8, 2.0 Hz, 1 H, 6-H), 4.18 (dd *J* = 7.0, 4.7 Hz, 1 H, C *H* CH _a_ H _b_ ), 3.09 (dd, *J* = 12.7, 4.7 Hz, 1 H, CHCH _a_ *H*_b_ ), 2.91 (dd, *J* = 12.7, 7.0 Hz, 1 H, CHC *H*_a_ H _b_ ), 1.67 (br s, 4 H, 2NH _2_ ), 1.66 (s, 9 H, OC(CH _3_ ) _3_ ).

^13^ C NMR (100 MHz, CDCl _3_ ): δ = 149.29, 134.60, 130.69, 127.29, 123.54, 122.82, 122.11, 116.83, 115.84, 84.08, 50.55, 48.13, 28.14 (3C).

MS (ESI): *m* / *z* = 356/354 [M + H] ^+^ , 339/337 [M – NH _3_ + H] ^+^ .

Anal. Calcd for C _15_ H _20_ BrN _3_ O _2_ : C, 50.86; H, 5.69; N, 11.86. Found: C, 50.80; H, 5.75; N, 11.79.

## 1-[(1- *tert* -Butoxycarbonyl)-1 *H* -indol-3-yl]- *N* ^1^ -methylethane-1,2-diamine (6c)

Obtained by following General Procedure B.

Yield: 0.59 g (93%); pale-yellow viscous oil; *R _f_* = 0.34 (CHCl _3_ –MeOH–NH _3(aq)_ , 10:1:0.02).

IR (film): 3366 br, 2976, 2932, 2794, 1728 s (CO), 1607, 1450, 1367, 1250, 1152, 1078, 1017, 854, 743, 665 cm ^–1^ .

^1^ H NMR (400 MHz, CDCl _3_ ): δ = 8.17 (d, *J* = 8.0 Hz, 1 H, 7-H), 7.66 (d, *J* = 7.7 Hz, 1 H, 4-H), 7.53 (s, 1 H, 2-H), 7.33 (dd, *J* = 8.0, 7.4 Hz, 1 H, 6-H), 7.23 (dd, *J* = 7.7, 7.4 Hz, 1 H, 5-H), 3.83 (dd *J* = 6.4, 5.4 Hz, 1 H, C *H* CH _a_ H _b_ ), 3.10 (dd, *J* = 12.7, 5.4 Hz, 1 H, CHCH _a_ *H*_b_ ), 2.98 (dd, *J* = 12.7, 6.4 Hz, 1 H, CHC *H*_a_ H _b_ ), 2.43 (s, 3 H, NCH _3_ ), 1.68 (s, 9 H, OC(CH _3_ ) _3_ ), 1.43 (br s, 3 H, NH + NH _2_ ).

^13^ C NMR (100 MHz, CDCl _3_ ): δ = 149.48, 135.68, 129.27, 124.25, 123.12, 122.23, 120.92, 119.33, 115.19, 83.39, 59.72, 46.04, 34.45, 28.00 (3C).

MS (ESI): *m* / *z* = 290 [M + H] ^+^ .

Anal. Calcd for C _16_ H _23_ N _3_ O _2_ : C, 66.41; H, 8.01; N, 14.52. Found: C, 66.33; H, 8.06; N, 14.45.

## 1-[5-Bromo-(1- *tert* -butoxycarbonyl)-1 *H* -indol-3-yl]- *N* ^1^ -methyl­ethane-1,2-diamine (6d)

Obtained by following General Procedure B.

Yield: 0.70 g (86%); pale-yellow viscous oil; *R _f_* = 0.46 (CHCl _3_ –MeOH–NH _3(aq)_ , 10:1:0.02).

IR (film): 3326 br, 2934, 2791, 1733 s (CO), 1602, 1449, 1373, 1275, 1256, 1156, 1055, 802, 732, 640, 611 cm ^–1^ .

^1^ H NMR (400 MHz, CDCl _3_ ): δ = 8.01 (d, *J* = 8.7 Hz, 1 H, 7-H), 7.81 (d, *J* = 1.9 Hz, 1 H, 4-H), 7.52 (s, 1 H, 2-H), 7.37 (dd, *J* = 8.7, 1.9 Hz, 1 H, 6-H), 3.76 (dd *J* = 6.7, 5.3 Hz, 1 H, C *H* CH _a_ H _b_ ), 3.06 (dd, *J* = 12.8, 5.3 Hz, 1 H, CHCH _a_ *H*_b_ ), 2.93 (dd, *J* = 12.8, 6.7 Hz, 1 H, CHC *H*_a_ H _b_ ), 2.39 (s, 3 H, NCH _3_ ), 1.69 (br s, 3 H, NH + NH _2_ ), 1.64 (s, 9 H, OC(CH _3_ ) _3_ ).

^13^ C NMR (100 MHz, CDCl _3_ ): δ = 149.19, 134.55, 131.07, 127.18, 124.42, 122.24, 120.28, 116.71, 115.77, 84.01, 59.62, 46.05, 34.42, 28.07 (3C).

MS (ESI): *m* / *z* = 370/368 [M + H] ^+^ .

Anal. Calcd for C _16_ H _22_ BrN _3_ O _2_ : C, 52.18; H, 6.02; N, 11.41. Found: C, 52.20; H, 6.06; N, 11.34.

## 1-[6-Bromo-(1- *tert* -butoxycarbonyl)-1 *H* -indol-3-yl]- *N* ^1^ -methyl­ethane-1,2-diamine (6e)

Obtained by following General Procedure B.

Yield: 0.74 g (92%); pale-yellow viscous oil; *R _f_* = 0.31 (CHCl _3_ –MeOH–NH _3(aq)_ , 10:1:0.02).

IR (film): 3320 br, 2977, 2935, 2792, 1736 s (CO), 1602, 1453, 1432, 1370, 1251, 1156, 1082, 810, 766, 590 cm ^–1^ .

^1^ H NMR (400 MHz, CDCl _3_ ): δ = 8.37 (br s, 1 H, 7-H), 7.55 (d, *J* = 8.4 Hz, 1 H, 4-H), 7.50 (s, 1 H, 2-H), 7.33 (dd, *J* = 8.4, 1.7 Hz, 1 H, 5-H), 3.81 (dd *J* = 6.8, 5.3 Hz, 1 H, C *H* CH _a_ H _b_ ), 3.08 (dd, *J* = 12.8, 5.3 Hz, 1 H, CHCH _a_ *H*_b_ ), 2.96 (dd, *J* = 12.8, 6.8 Hz, 1 H, CHC *H*_a_ H _b_ ), 2.41 (s, 3 H, NCH _3_ ), 1.76 (br s, 3 H, NH + NH _2_ ), 1.67 (s, 9 H, OC(CH _3_ ) _3_ ).

^13^ C NMR (100 MHz, CDCl _3_ ): δ = 149.23, 136.56, 128.20, 125.69, 123.79, 120.75, 120.69, 118.59, 118.25, 84.23, 59.65, 46.11, 34.43, 28.12 (3C).

MS (ESI): *m* / *z* = 370/368 [M + H] ^+^ .

Anal. Calcd for C _16_ H _22_ BrN _3_ O _2_ : C, 52.18; H, 6.02; N, 11.41. Found: C, 52.27; H, 6.07; N, 11.30.

## 1-[5-Methoxy-(1- *tert* -butoxycarbonyl)-1 *H* -indol-3-yl]- *N* ^1^ -methyl­ethane-1,2-diamine (6f)

Obtained by following General Procedure B.

Yield: 0.53 g (75%); pale-yellow viscous oil; *R _f_* = 0.34 (CHCl _3_ –MeOH–NH _3(aq)_ , 10:1:0.02).

IR (film): 3325 br, 2976, 2836, 2794, 1730 s (CO), 1612, 1477, 1449, 1385, 1256, 1159, 1074, 855, 803, 732, 656 cm ^–1^ .

^1^ H NMR (400 MHz, CDCl _3_ ): δ = 8.02 (d, *J* = 9.0 Hz, 1 H, 7-H), 7.51 (s, 1 H, 2-H), 7.12 (d, *J* = 2.5 Hz, 1 H, 4-H), 6.92 (dd, *J* = 9.0, 2.5 Hz, 1 H, 6-H), 3.84 (s, 3 H, OCH _3_ ), 3.81 (dd *J* = 6.5, 5.5 Hz, 1 H, C *H* CH _a_ H _b_ ), 3.09 (dd, *J* = 12.9, 5.5 Hz, 1 H, CHCH _a_ *H*_b_ ), 2.97 (dd, *J* = 12.9, 6.5 Hz, 1 H, CHC *H*_a_ H _b_ ), 2.42 (s, 3 H, NCH _3_ ), 1.91 (br s, 3 H, NH + NH _2_ ), 1.65 (s, 9 H, OC(CH _3_ ) _3_ ).

^13^ C NMR (100 MHz, CDCl _3_ ): δ = 155.68, 149.53, 130.52, 130.17, 123.99, 120.33, 115.99, 112.89, 102.46, 83.42, 59.54, 55.73, 45.85, 34.32, 28.13 (3C).

MS (ESI): *m* / *z* = 320 [M + H] ^+^ .

Anal. Calcd for C _17_ H _25_ N _3_ O _3_ : C, 63.93; H, 7.89; N, 13.16. Found: C, 63.81; H, 7.96; N, 13.10.

## 1-[6-Chloro-(1- *tert* -butoxycarbonyl)-1 *H* -indol-3-yl]- *N* ^1^ -methyl­ethane-1,2-diamine (6g)

Obtained by following General Procedure B.

Yield: 0.655 g (92%); pale-yellow viscous oil; *R _f_* = 0.32 (CHCl _3_ –MeOH–NH _3(aq)_ , 10:1:0.02).

IR (film): 3366 br, 2978, 2937, 2796, 1737 s (CO), 1606, 1435, 1455, 1371, 1252, 11576, 1087, 812, 767, 733, 595 cm ^–1^ .

^1^ H NMR (400 MHz, CDCl _3_ ): δ = 8.18 (br s, 1 H, 7-H), 7.57 (d, *J* = 8.3 Hz, 1 H, 4-H), 7.50 (s, 1 H, 2-H), 7.16 (dd, *J* = 8.3, 2.0 Hz, 1 H, 5-H), 3.80 (dd *J* = 6.7, 5.3 Hz, 1 H, C *H* CH _a_ H _b_ ), 3.06 (dd, *J* = 12.8, 5.3 Hz, 1 H, CHCH _a_ *H*_b_ ), 2.94 (dd, *J* = 12.8, 6.7 Hz, 1 H, CHC *H*_a_ H _b_ ), 2.38 (s, 3 H, NCH _3_ ), 2.01 (br s, 3 H, NH + NH _2_ ), 1.64 (s, 9 H, OC(CH _3_ ) _3_ ).

^13^ C NMR (100 MHz, CDCl _3_ ): δ = 149.24, 136.24, 130.48, 127.83, 123.91, 123.01, 120.61, 120.33, 115.67, 84.21, 59.46, 45.99, 34.37, 28.12 (3C).

MS (ESI): *m* / *z* = 326/324 [M + H] ^+^ .

Anal. Calcd for C _16_ H _22_ ClN _3_ O _2_ : C, 59.35; H, 6.85; N, 12.98. Found: C, 59.27; H, 6.90; N, 12.91.

## *tert* -Butyl 6-Bromo-3-{2-[(6-bromo-1 *H* -indol-3-yl)carbonyl]-1-methyl-4,5-dihydro-1 *H* -imidazol-5-yl}-1 *H* -indole-1-carboxylate (16a)

A solution of 3-acetyl-6-bromoindole (0.2 g, 0.84 mmol) and I _2_ (0.23 g, 0.92 mmol) in DMSO (1.7 mL) was heated for 2 h at 100 °C. The reaction mixture was cooled to 0–5 °C then treated with NaHCO _3_ (0.08 g, 0.92 mmol) and a solution of diamine **6e** (0.32 g, 0.86 mmol) in MeCN (8.5 mL). After stirring for 0.5 h the reaction mixture was treated with NCS (0.11 g, 0.86 mmol) under cooling (0–5 °C), stirred for 20 min, and left to stand overnight. The reaction mixture was diluted with CHCl _3_ (50 mL), washed with saturated NaHCO _3_ (30 mL) and Na _2_ S _2_ O _3_ (30 mL) solutions, H _2_ O (5 × 30 mL), and concd NaCl solution (30 mL), and dried over anhydrous Na _2_ SO _4_ . The solvent was removed in vacuo and the solid was purified by chromatography on a column with silica gel (СHCl _3_ –MeOH, 100:1) to provide the title compound.

Yield: 0.388 g (77%); brownish amorphous solid; *R _f_* = 0.53 (СHCl _3_ –MeOH, 40:1).

IR (film): 3119, 2978, 2869, 1742 s (CO), 1630, 1574, 1434, 1369, 1249, 1155, 1086, 980, 810, 689, 591 cm ^–1^ .

^1^ H NMR (500 MHz, CDCl _3_ ): δ = 12.14 (br s, 1 H, NH), 8.41 (br s, 1 H), 8.17 (d, *J* = 8.2 Hz, 1 H), 7.98 (s, 1 H), 7.62 (s, 1 H), 7.44 (s, 1 H), 7.29–7.41 (m, 3 H), 4.91 (dd *J* = 12.2, 10.4 Hz, 1 H, C *H* CH _a_ H _b_ ), 4.17 (dd, *J* = 14.4, 12.2 Hz, 1 H, CHCH _a_ *H*_b_ ), 3.80 (dd, *J* = 14.4, 10.4 Hz, 1 H, CHC *H*_a_ H _b_ ), 2.81 (s, 3 H, NCH _3_ ), 1.70 (s, 9 H, OC(CH _3_ ) _3_ ).

^13^ C NMR (100 MHz, CDCl _3_ ): δ = 180.54, 163.28, 149.06, 137.87, 137.63, 136.93, 126.62, 126.42 (2C), 124.88, 124.57, 123.46, 120.55, 118.96, 118.93, 118.39, 117.63, 115.60, 115.24, 84.82, 61.00, 58.76, 31.86, 28.16 (3C).

MS (ESI): *m* / *z* = 602/601/600 [M + H] ^+^ .

Anal. Calcd for C _26_ H _24_ Br _2_ N _4_ O _3_ : C, 52.02; H, 4.03; N, 9.33. Found: C, 52.21; H, 4.11; N, 9.21.

## *tert* -Butyl 3-[2-(1 *H* -Indol-3-ylcarbonyl)-1-methyl-4,5-dihydro-1 *H* -imidazol-5-yl]-1 *H* -indole-1-carboxylate (16b)

The compound was prepared as described above for **16a** .

Yield: 0.290 (79%); light-brown amorphous solid; *R _f_* = 0.46 (СHCl _3_ –MeOH, 40:1).

IR (film): 3105 br, 2979, 2932, 1737 s (CO), 1640, 1607, 1514, 1451, 1369, 1240, 1154, 1091, 749, 589 cm ^–1^ .

^1^ H NMR (400 MHz, CDCl _3_ ): δ = 12.40 (br s, 1 H, NH), 8.42 (d, *J* = 7.9 Hz, 1 H), 8.21 (d, *J* = 8.3 Hz, 1 H), 7.88 (s, 1 H), 7.58–7.67 (m, 2 H), 7.34–7.41 (m, 1 H), 7.20–7.29 (m, 3 H), 7.13–7.19 (m, 1 H), 4.82 (dd *J* = 11.6, 10.6 Hz, 1 H, C *H* CH _a_ H _b_ ), 4.15 (dd, *J* = 14.5, 11.6 Hz, 1 H, CHCH _a_ *H*_b_ ), 3.88 (dd, *J* = 14.5, 10.6 Hz, 1 H, CHC *H*_a_ H _b_ ), 2.83 (s, 3 H, NCH _3_ ), 1.72 (s, 9 H, OC(CH _3_ ) _3_ ).

^13^ C NMR (100 MHz, CDCl _3_ ): δ = 180.93, 163.58, 149.51, 137.72, 137.20, 136.18, 127.99, 125.79, 124.95, 124.30, 123.97, 123.08, 123.05, 122.16, 119.58, 118.71, 115.69, 115.58, 112.29, 84.13, 61.08, 58.78, 31.86, 28.19 (3C).

MS (ESI): *m* / *z* = 443 [M + H] ^+^ .

Anal. Calcd for C _26_ H _26_ N _4_ O _3_ : C, 70.57; H, 5.92; N, 12.66. Found: C, 70.41; H, 5.84; N, 12.55.

## *tert* -Butyl 6-Bromo-3-[5-(6-bromo-1 *H* -indol-3-yl)-1-methyl-6-oxo-1,2,3,6-tetrahydropyrazin-2-yl]-1 *H* -indole-1-carboxylate (22a)

A solution of diamine **6e** (0.26 g, 0.70 mmol) in EtOH (2.6 mL) at 0–5 °C was treated portionwise with acid chloride **18a** (0.20 g, 0.7 mmol) over a period of 10 min and stirred for 15 min at r.t. Anhydrous AcONa (57.4 mg, 0.7 mmol) and AcOH (0.26 mL) were then added and the reaction mixture was heated at reflux for 1.5 h. After cooling, the mixture was diluted with EtOAc (25 mL), washed with saturated NaHCO _3_ (20 mL) and concd NaCl (10 mL) solutions, and dried over anhydrous Na _2_ SO _4_ . The solvent was removed in vacuo and the residue was purified by chromatography on a column with silica gel (toluene–EtOAc, 3:1) to provide the title compound.

Yield: 0.302 g (72%); pale-yellow amorphous solid; *R _f_* = 0.49 (toluene–EtOAc, 2:1).

IR (film): 3151 br, 2976, 2932, 1740 s (CO), 1650, 1590, 1434, 1369, 1252, 1154, 1088, 809, 766, 731, 589 cm ^–1^ .

^1^ H NMR (600 MHz, CDCl _3_ ): δ = 8.89 (br s, 1 H, NH), 8.47 (d, *J* = 2.6 Hz, 1 H), 8.35 (br s, 1 H), 8.27 (d, *J* = 8.8 Hz, 1 H), 7.54 (d, *J* = 1.8 Hz, 1 H), 7.45 (d, *J* = 8.4 Hz, 1 H), 7.42 (br s, 1 H), 7.40 (dd, *J* = 8.4, 1.8 Hz, 1 H), 7.28 (dd, *J* = 8.8, 1.8 Hz, 1 H), 4.96 (dd *J* = 5.9, 5.5 Hz, 1 H, C *H* CH _a_ H _b_ ), 4.38 (dd, *J* = 16.5, 5.9 Hz, 1 H, CHCH _a_ *H*_b_ ), 4.31 (dd, *J* = 16.5, 5.5 Hz, 1 H, CHC *H*_a_ H _b_ ), 3.10 (s, 3 H, NCH _3_ ), 1.60 (s, 9 H, OC(CH _3_ ) _3_ ).

^13^ C NMR (125 MHz, CDCl _3_ ): δ = 157.84, 157.21, 148.91, 136.90, 136.52, 131.99, 127.18, 126.25, 125.22, 124.65, 124.54, 124.10, 119.98, 118.94, 118.79, 117.00, 116.40, 114.11, 112.13, 84.90, 53.31, 51.87, 33.00, 28.00 (3C).

MS (ESI): *m* / *z* = 602/601/600 [M + H] ^+^ .

Anal. Calcd for C _26_ H _24_ Br _2_ N _4_ O _3_ : C, 52.02; H, 4.03; N, 9.33. Found: C, 52.24; H, 4.11; N, 9.24.

## *tert* -Butyl 3-[5-(1 *H* -Indol-3-yl)-1-methyl-6-oxo-1,2,3,6-tetrahydropyrazin-2-yl]-1 *H* -indole-1-carboxylate (22b)

The compound was prepared as described for **22a** .

Yield: 0.214 g (69 %); light-brown amorphous solid; *R _f_* = 0.45 (toluene–EtOAc, 2:1).

IR (film): 3270 br, 2976, 2931, 1736 s (CO), 1650, 1590, 1453, 1371, 1310, 1256, 1155, 1088, 852, 746, 591 cm ^–1^ .

^1^ H NMR (400 MHz, CDCl _3_ ): δ = 8.88 (br s, 1 H, NH), 8.54 (d, *J* = 2.6 Hz, 1 H), 8.43 (d, *J* = 8.7 Hz, 1 H), 8.14 (d, *J* = 8.0 Hz, 1 H), 7.61 (d, *J* = 7.7 Hz, 1 H), 7.45 (s, 1 H), 7.33–7.42 (m, 2 H), 7.25–7.31 (m, 1 H), 7.17–7.25 (m, 2 H), 4.98 (dd, *J* = 5.9, 5.4 Hz, 1 H, C *H* CH _a_ H _b_ ), 4.46 (dd, *J* = 16.5, 5.9 Hz, 1 H, CHCH _a_ *H*_b_ ), 4.31 (dd, *J* = 16.5, 5.4 Hz, 1 H, CHC *H*_a_ H _b_ ), 3.13 (s, 3 H, NCH _3_ ), 1.60 (s, 9 H, OC(CH _3_ ) _3_ ).

^13^ C NMR (100 MHz, CDCl _3_ ): δ = 158.07, 157.29, 149.36, 136.09, 135.87, 132.13, 128.44, 126.23, 124.93, 124.18, 122.94 (2C), 122.70, 121.57, 118.93, 117.00, 115.67, 111.94, 111.19, 84.20, 53.45, 51.63, 32.96, 28.06 (3C).

MS (ESI): *m* / *z* = 443 [M + H] ^+^ .

Anal. Calcd for C _26_ H _26_ N _4_ O _3_ : C, 70.57; H, 5.92; N, 12.66. Found: C, 70.71; H, 5.99; N, 12.60.

## Removal of the Boc Protecting Group; General Procedure

A suspension of the substrate (0.43 mmol) in CH _2_ Cl _2_ (2 mL) at 0–5 °C was treated with TFA (0.4 mL, 5.2 mmol) in four portions, stirred at the same temperature for 20 min, left overnight, and evaporated in vacuo. The residue was dissolved in EtOAc (40 mL), washed with saturated NaHCO _3_ (2 × 20 mL) and concd NaCl (20 mL) solutions, and dried over anhydrous Na _2_ SO _4_ . The solvent was removed in vacuo and the residue was purified by chromatography on a column with silica gel.

## (6-Bromo-1 *H* -indol-3-yl)[5-(6-bromo-1 *H* -indol-3-yl)-1-methyl-4,5-dihydro-1 *H* -imidazol-2-yl]methanone (5a)

Yield: 0.210 g (98%); light-beige solid; mp 247–249 °C (dec.) (EtOAc); *R _f_* = 0.36 (CHCl _3_ –MeOH, 20:1).

IR (film): 3426, 3119, 3012, 2850, 1637, 1569, 1513, 1450, 1265, 1085, 986, 822, 793, 692, 600 cm ^–1^ .

^1^ H NMR (400 MHz, DMSO- *d*_6_ ): δ = 12.30 (br s, 1 H, 1′-NH), 11.20 (br s, 1 H, 1′′-NH), 8.51 (s, 1 H, 2′-H), 8.17 (d, *J* = 8.4 Hz, 1 H, 4′-H), 7.74 (d, *J* = 1.6 Hz, 1 H, 7′-H), 7.59 (d, *J* = 1.6 Hz, 1 H, 7′′-H), 7.53 (d, *J* = 8.4 Hz, 1 H, 4′′-H), 7.45 (d, *J* = 2.4 Hz, 1 H, 2′′-H), 7.40 (dd, *J* = 8.4, 1.6 Hz, 1 H, 5′-H), 7.14 (dd, *J* = 8.4, 1.6 Hz, 1 H, 5′′-H), 4.86 (dd *J* = 11.2, 10.3 Hz, 1 H, C *H* CH _a_ H _b_ ), 4.26 (dd, *J* = 15.1, 11.2 Hz, 1 H, CHCH _a_ *H*_b_ ), 3.80 (dd, *J* = 15.1, 10.3 Hz, 1 H, CHC *H*_a_ H _b_ ), 2.67 (s, 3 H, NCH _3_ ).

^13^ C NMR (100 MHz, DMSO- *d*_6_ ): δ = 182.24, 162.09, 139.09, 137.80, 137.60, 125.27 (2C), 124.65, 124.43, 122.90, 121.67, 120.56, 115.82, 115.23, 114.80, 114.40, 114.13, 113.88, 60.54 (2C), 31.69.

^1^ H NMR (400 MHz, acetone- *d*_6_ ): δ = 11.37 (br s, 1 H, 1′-NH), 10.42 (br s, 1 H, 1′′-NH), 8.68 (s, 1 H, 2′-H), 8.32 (d, *J* = 8.5 Hz, 1 H, 4′-H), 7.76 (d, *J* = 1.6 Hz, 1 H, 7′-H), 7.65 (d, *J* = 1.6 Hz, 1 H, 7′′-H), 7.63 (d, *J* = 8.5 Hz, 1 H, 4′′-H), 7.45 (d, *J* = 2.1 Hz, 1 H, 2′′-H), 7.41 (dd, *J* = 8.5, 1.7 Hz, 1 H, 5′-H), 7.17 (dd, *J* = 8.5, 1.7 Hz, 1 H, 5′′-H), 4.90 (dd *J* = 11.3, 10.3 Hz, 1 H, C *H* CH _a_ H _b_ ), 4.31 (dd, *J* = 15.3, 11.3 Hz, 1 H, CHCH _a_ *H*_b_ ), 3.91 (dd, *J* = 15.3, 10.3 Hz, 1 H, CHC *H*_a_ H _b_ ), 2.82 (s, 3 H, NCH _3_ ).

^13^ C NMR (100 MHz, acetone- *d*_6_ ): δ = 183.28, 163.42, 139.61, 139.36, 138.78, 126.40, 126.31, 125.93, 125.78, 124.34, 123.13, 121.83, 117.27, 116.72, 116.13, 115.88, 115.83, 115.59, 62.40, 61.88, 32.51.

MS (ESI): *m* / *z* = 502/501/500 [M + H] ^+^ .

Anal. Calcd for C _21_ H _16_ Br _2_ N _4_ O: C, 50.43; H, 3.22; N, 11.20. Found: C, 50.49; H, 3.26; N, 11.18.

## 1 *H* -Indol-3-yl[5-(1 *H* -indol-3-yl)-1-methyl-4,5-dihydro-1 *H* -imidazol-2-yl]methanone (5b)

Yield: 0.139 g (94%); light-brown amorphous solid; *R _f_* = 0.33 (CHCl _3_ –MeOH, 20:1).

IR (film): 3360 br, 3059, 2919, 1620, 1580, 1518, 1442, 1241, 1085, 977, 858, 743, 642 cm ^–1^ .

^1^ H NMR (400 MHz, DMSO- *d*_6_ ): δ = 12.22 (br s, 1 H, 1′-NH), 11.08 (br s, 1 H, 1′′-NH), 8.50 (s, 1 H), 8.24–8.29 (m, 1 H), 7.61 (d, *J* = 8.0 Hz, 1 H), 7.52–7.58 (m, 1 H), 7.37–7.44 (m, 2 H), 7.22–7.32 (m, 2 H), 7.08–7.15 (m, 1 H), 6.97–7.04 (m, 1 H), 4.88 (dd *J* = 11.3, 10.2 Hz, 1 H, C *H* CH _a_ H _b_ ), 4.26 (dd, *J* = 14.8, 11.3 Hz, 1 H, CHCH _a_ *H*_b_ ), 3.86 (dd, *J* = 14.8, 10.2 Hz, 1 H, CHC *H*_a_ H _b_ ), 2.68 (s, 3 H, NCH _3_ ).

^13^ C NMR (100 MHz, DMSO- *d*_6_ ): δ = 182.32, 162.48, 138.42, 137.02, 137.79, 125.62, 125.50, 124.34, 123.46, 122.52, 121.36 (2C), 118.94, 118.83, 115.01, 113.41, 112.60, 111.92, 60.83, 60.36, 31.73.

MS (ESI): *m* / *z* = 343 [M + H] ^+^ .

Anal. Calcd for C _21_ H _18_ N _4_ O: C, 73.67; H, 5.30; N, 16.36. Found: C, 73.48; H, 5.23; N, 16.28.

## Topsentin C (17a)

Yield: 0.214 g (99%); pale-yellow solid; mp 179–181 °C (dec.) ( *i* -PrOH); *R _f_* = 0.18 (CHCl _3_ ).

IR (film): 3404, 3208, 2924, 2853, 1647, 1592, 1524, 1440, 1397, 1340, 1290, 1231, 1111, 1049, 947, 895, 852, 793, 734, 647, 570 cm ^–1^ .

^1^ H NMR (400 MHz, DMSO- *d*_6_ ): δ = 11.62 (br s, 1 H, 1′-NH), 11.14 (br s, 1 H, 1′′-NH), 8.46 (d, *J* = 2.4 Hz, 1 H, 2′-H), 8.22 (d, *J* = 8.6 Hz, 1 H, 4′-H), 7.49–7.71 (m, 3 H, 7′-H + 7′′-H + 4′′-H), 7.03–7.25 (m, 3 H, 2′′-H + 5′-H + 5′′-H), 5.11 (dd, *J* = 5.3, 4.8 Hz, 1 H, C *H* CH _a_ H _b_ ), 4.29 (dd, *J* = 16.5, 4.8 Hz, 1 H, CHCH _a_ *H*_b_ ), 4.18 (dd, *J* = 16.5, 5.3 Hz, 1 H, CHC *H*_a_ H _b_ ), 2.97 (s, 3 H, NCH _3_ ).

^13^ C NMR (100 MHz, DMSO- *d*_6_ ): δ = 157.08, 156.69, 137.42, 137.04, 132.77, 125.09, 124.64, 124.52, 124.10, 123.21, 121.83, 120.33, 114.70, 114.35, 114.20 (2C), 112.12, 111.02, 52.46, 52.28, 32.36.

^1^ H NMR (400 MHz, acetone- *d*_6_ ): δ = 10.70 (br s, 1 H, 1′-NH), 10.34 (br s, 1 H, 1′′-NH), 8.62 (d, *J* = 2.75 Hz, 1 H, 2′-H), 8.37 (d, *J* = 8.6 Hz, 1 H, 4′-H), 7.69 (d, *J* = 8.6 Hz, 1 H, 4′′-H), 7.66 (d, *J* = 1.8 Hz, 1 H, 7′-H), 7.63 (d, *J* = 1.7 Hz, 1 H, 7′′-H), 7.17–7.23 (m, 3 H, 2′′-H + 5′-H + 5′′-H), 5.15 (dd *J* = 5.5, 5.2 Hz, 1 H, C *H* CH _a_ H _b_ ), 4.41 (dd, *J* = 16.5, 5.2 Hz, 1 H, CHCH _a_ *H*_b_ ), 4.26 (dd, *J* = 16.5, 5.5 Hz, 1 H, CHC *H*_a_ H _b_ ), 3.04 (s, 3 H, NCH _3_ ).

^13^ C NMR (100 MHz, acetone- *d*_6_ ): δ = 158.47, 158.06, 138.86, 138.34, 133.67, 126.67, 126.07, 125.49, 125.39, 124.40, 123.30, 121.19, 116.17, 115.82, 115.53, 115.09, 113.93, 113.05, 54.07, 53.54, 32.77.

MS (ESI): *m* / *z* = 502/501/500 [M + H] ^+^ .

Anal. Calcd for C _21_ H _16_ Br _2_ N _4_ O: C, 50.43; H, 3.22; N, 11.20. Found: C, 50.55; H, 3.25; N, 11.16.

## 3,6-Di-1 *H* -Indol-3-yl-1-methyl-5,6-dihydropyrazin-2(1 *H* )-one (17b)

Yield: 0.141 g (96%); *R _f_* = 0.60 (CHCl _3_ –MeOH, 15:1).

IR (film): 3400, 3271, 3055, 2926, 1644, 1589, 1422, 1339, 1239, 1172, 1100, 1011, 851, 744 cm ^–1^ .

^1^ H NMR (600 MHz, DMSO- *d*_6_ ): δ = 11.53 (br s, 1 H, NH), 11.01 (br s, 1 H, NH), 8.44 (d, *J* = 3.3 Hz, 1 H), 8.29 (d, *J* = 8.3 Hz, 1 H), 7.64 (d, *J* = 8.3 Hz, 1 H), 7.42 (d, *J* = 8.3 Hz, 1 H), 7.36 (d, *J* = 8.3 Hz, 1 H), 7.08–7.17 (m, 2 H), 7.07 (d, *J* = 2.5 Hz, 1 H), 6.98–7.05 (m, 2 H), 5.11 (dd, *J* = 5.8, 4.9 Hz, 1 H, C *H* CH _a_ H _b_ ), 4.30 (dd, *J* = 16.5, 4.9 Hz, 1 H, CHCH _a_ *H*_b_ ), 4.19 (dd, *J* = 16.5, 5.8 Hz, 1 H, CHC *H*_a_ H _b_ ), 2.99 (s, 3 H, NCH _3_ ).

^13^ C NMR (125 MHz, DMSO- *d*_6_ ): δ = 157.43, 157.02, 136.63, 136.17, 132.06, 126.14, 125.73, 123.46, 122.54, 122.07, 121.46, 120.44, 119.09, 118.59, 111.85 (2C), 111.61, 111.06, 52.78, 52.40, 32.47.

MS (ESI): *m* / *z* = 343 [M + H] ^+^ .

Anal. Calcd for C _21_ H _18_ N _4_ O: C, 73.67; H, 5.30; N, 16.36. Found: C, 73.79; H, 5.34; N, 16.31.
