# Supplementary material for: Revision of the Structure and Total Synthesis of Topsentin C
Source: Synthesis (Stuttg). 2017 Feb 23;49(11):2562–74. doi: 10.1055/s-0036-1588731 (PMC6193241; doi:10.1055/s-0036-1588731)
Supplement: Supplementary file 1 — Supporting Information [file sup_ss-2016-n0804-op_10-1055_s-0036-1588731.pdf]

Synthesis

Reviews and Full Papers in Chemical Synthesis

Supporting Information

for DOI: 10.1055/s-0036-1588731

© Georg Thieme Verlag KG Stuttgart · New York 2017

Thieme

Supporting Information

Revision of the structure and total synthesis of topsentin C

Nikita E. Golantsov, Alexey A. Festa, Alexey A. Varlamov, Leonid G. Voskressensky*

Peoples' Friendship University of Russia (RUDN University), 6, Miklukho-Maklaya St., Moscow, Russia 117198

1. Comparison of NMR data for natural topsentin C and synthetic compound **5a** and **17a** 2

2. Copies of ^{1}H and ^{13}C NMR spectra 4

3. X-Ray data for topsentin C (**17a**) 38

1. Comparison of NMR data for natural topsentin C[1] and synthetic compound **5a** and **17a**

Previously proposed structure of topsentine C

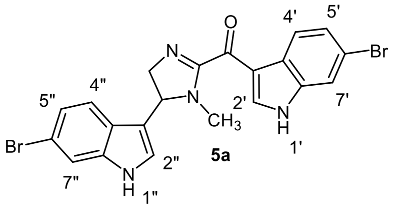

Revised structure of topsentin C

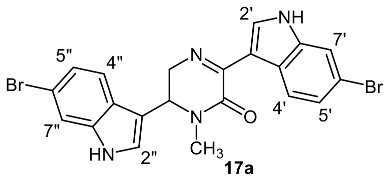

^{1}H NMR data for compounds **5a**, **17a** and natural topsentin C (400 MHz, acetone-d_6)

^{1}H	**5a**	**17a**	Natural topsentin C
N(1)CH_3	2.82 (s)	3.04 (s)	3.05 (s)
CHC$H_a$$H_b$	3.91 (dd, J = 15.3, 10.3)	4.26 (dd, J = 16.5, 5.5)	4.27 (dd, J = 16.5, 5.3)
CHC$H_a$$H_b$	4.31 (dd, J = 15.3, 11.3)	4.41 (dd, J = 16.5, 5.2)	4.41 (dd, J = 16.5, 5.2)
CHC$H_a$$H_b$	4.89 (dd, J = 11.3, 10.3)	5.15 (dd, J = 5.5, 5.2)	5.16 (ddd, J = 5.3, 5.2, <1)
1’	11.37 (br s)	10.70 (br s)	10.71 (br s)
2’	8.68 (s)	8.62 (d, J = 2.75)	8.62 (d, J = 2.7)
4’	8.32 (d, J = 8.5)	8.37 (d, J = 8.6)	8.37 (d, J = 8.7)
5’	7.41 (dd, J = 8.5, 1.7)	7.17-7.23 (m)[b]	7.20 (dd, J = 8.7, 1.8)
7’	7.76 (d, J = 1.6)	7.66 (d, J = 1.8)	7.66 (d, J = 1.8)
1”	10.42 (br s)	10.34 (br s)	10.34 (br s)
2”	7.45 (d, J = 2.1)	7.17-7.23 (m)[b]	7.22 (dd, J = 2.5, <1)
4”	7.63 (d, J = 8.5)	7.69 (d, J = 8.6)	7.69 (d, J = 8.5)
5”	7.17 (dd, J = 8.5, 1.7)	7.17-7.23 (m)[b]	7.20 (dd, J = 8.5, 1.7)
7”	7.65 (d, J = 1.6)	7.63 (d, J = 1.7)	7.63 (d, J = 1.7)

[1] Morris, S. A.; Andersen, R. J. *Tetrahedron*, **1990**, 46, 715.

^{13}C NMR data for compounds **17a**, **5a** (100 MHz, acetone-d_6)

^{13}C	**5a**	**17a**
N(1)CH_3	32.51	32.77
CH*C*H_2	61.88	53.54
*C*HCH_2	62.40	54.07
C=N	163.42	158.06
C=O	183.28	158.47
2’	139.61	133.67
3’	117.27	113.05
3a’	126.31	126.67
4’	124.34	125.49
5’	126.40	124.40
6’	117.27	116.17
7’	116.13	115.09
7a’	138.78	138.34
2”	125.93	125.39
3”	116.72	113.93
3a”	125.78	126.07
4”	121.83	121.19
5”	123.13	123.30
6”	115.83	115.82
7”	115.59	115.53
7a”	139.36	138.86

^{13}C NMR data for natural topsentin C (75 MHz, acetone-d^6): 32.8; 53.5; 54.0; 115.0; 115.1; 115.5; 115.8; 116.2; 121.2; 123.3; 123.7; 124.4; 125.3; 125.4; 125.5; 126.1; 129.8; 132.0; 133.8; 157.8; 158.0.[2]

[2] There are differences in chemical shifts of several low intensity signals of tertiary carbon atoms in ^{13}C spectra. It is obvious, that due to a very small amount of isolated topsentin C, background signals or admixtures masked these low peaks in spectrum of the natural compound.

2. Copies of ^{1}H and ^{13}C NMR Spectra

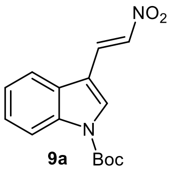

9a

DMSO-d$_6$

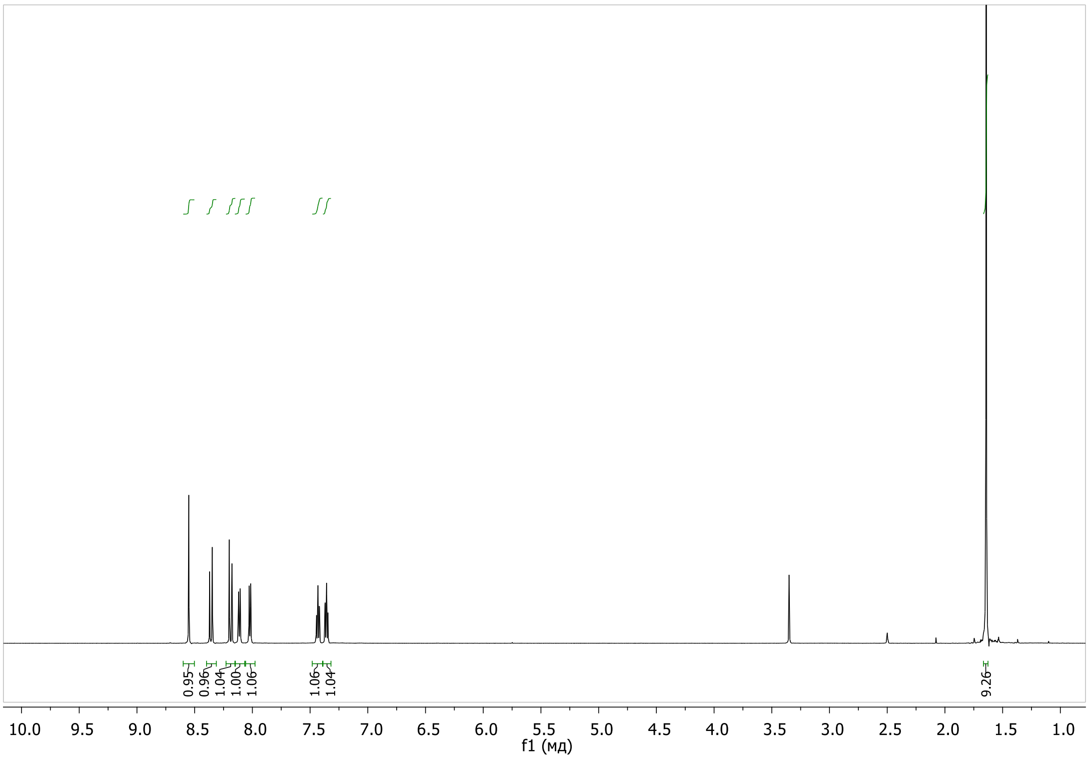

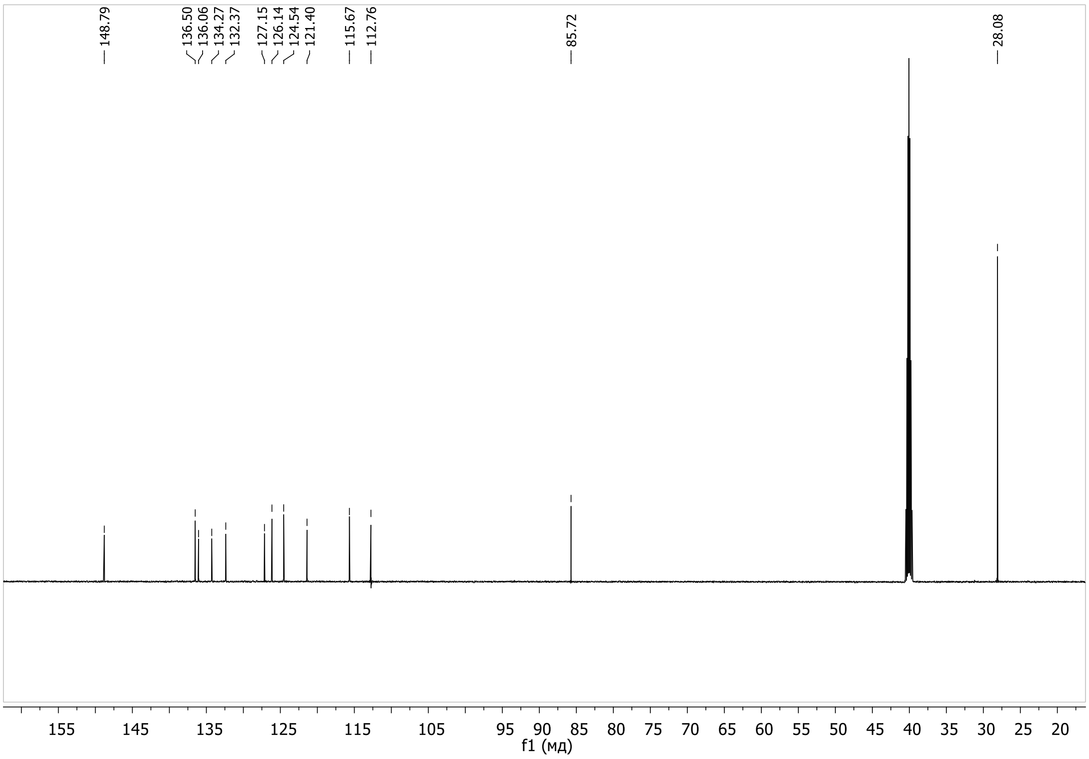

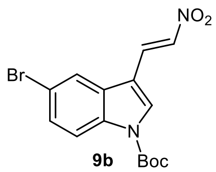
NO2
Br
N
Boc

9b

DMSO-d$_6$

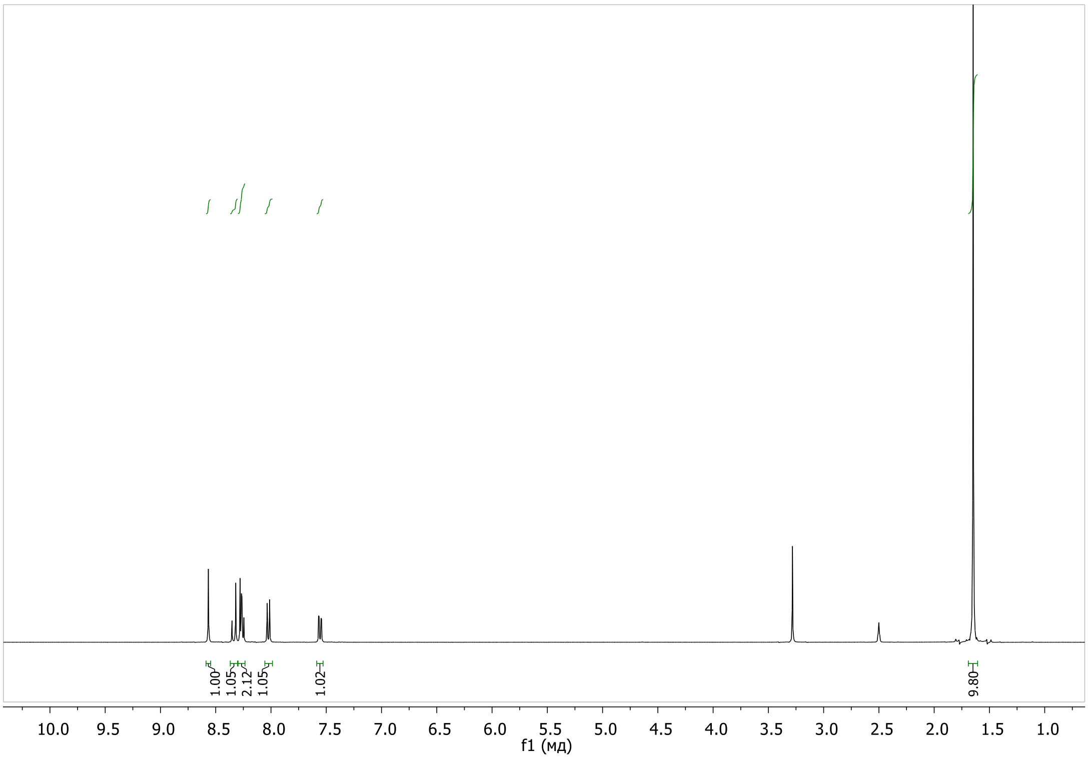
1.00
1.05
2.12
1.05
1.02
9.80
10.0 9.5 9.0 8.5 8.0 7.5 7.0 6.5 6.0 5.5 5.0 4.5 4.0 3.5 3.0 2.5 2.0 1.5 1.0
f1 (мд)

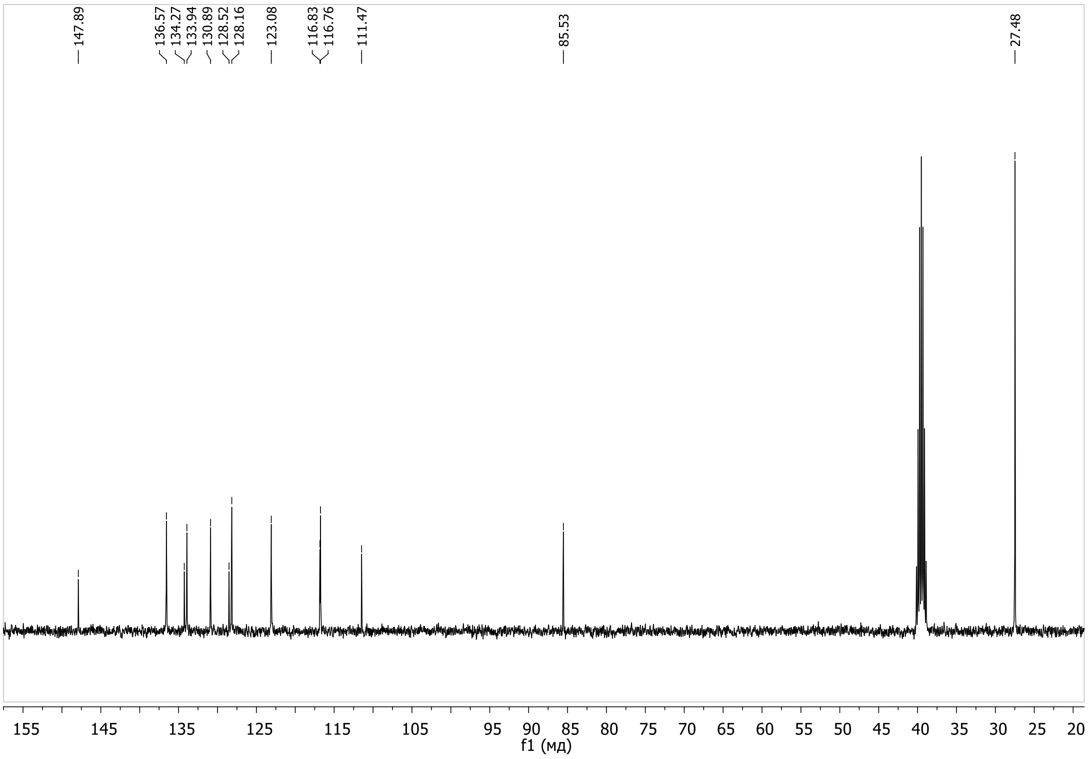
147.89
136.57
134.27
133.94
130.89
128.52
128.16
123.08
116.83
116.76
111.47
85.53
27.48
155 145 135 125 115 105 95 90 85 80 75 70 65 60 55 50 45 40 35 30 25 20
f1 (мд)

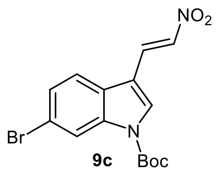
NO2
Br
N
9c
Boc

CDCl$_3$

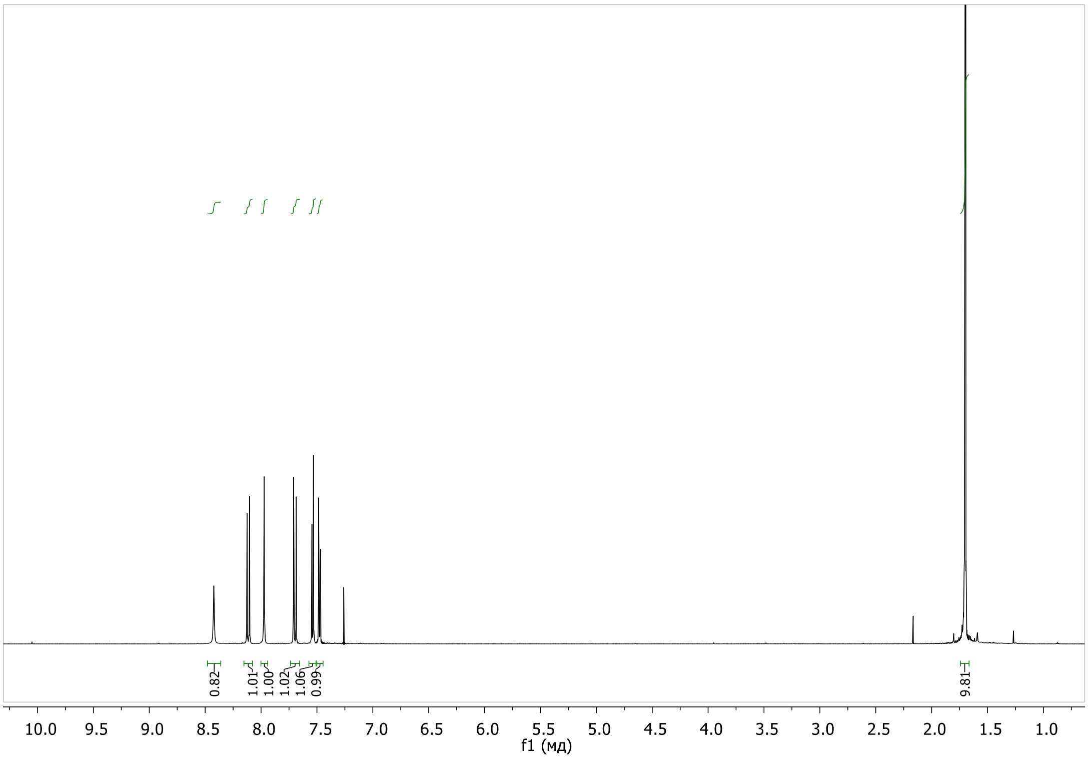
0.82
1.01
1.00
1.02
1.06
0.99
9.81
10.0
9.5
9.0
8.5
8.0
7.5
7.0
6.5
6.0
5.5
5.0
4.5
4.0
3.5
3.0
2.5
2.0
1.5
1.0
f1 (мд)

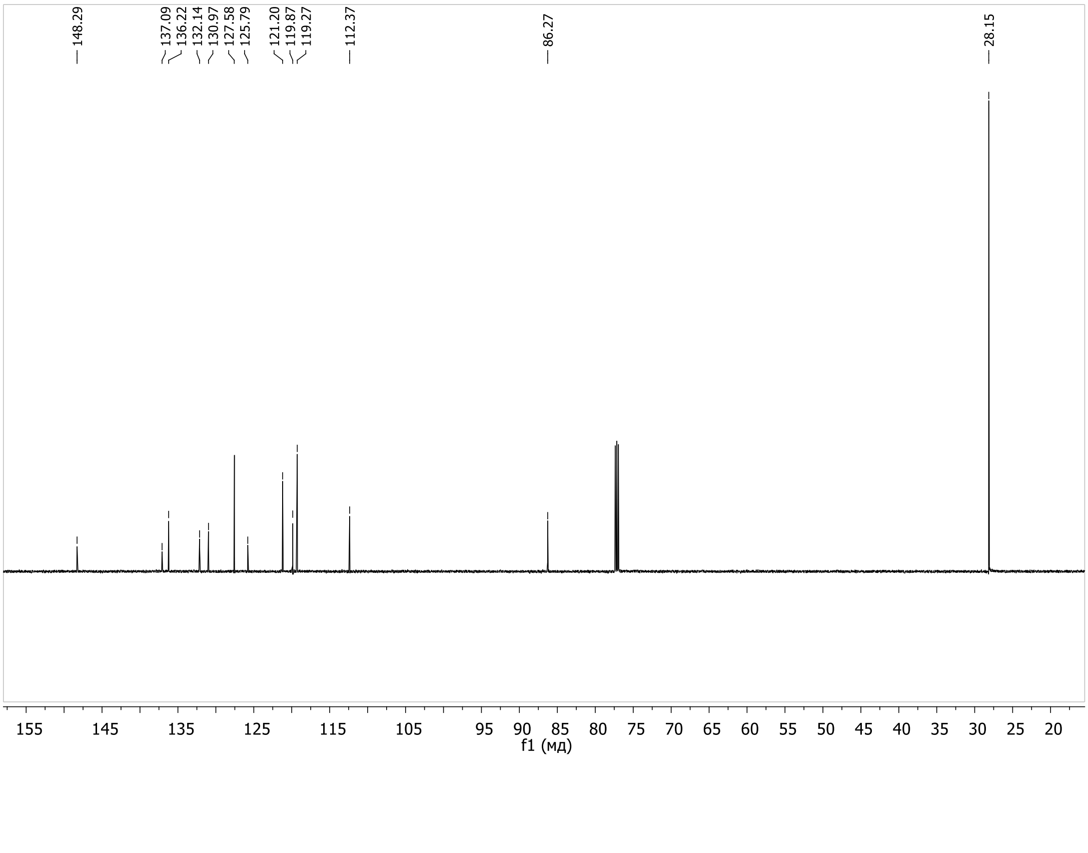
148.29
137.09
136.22
132.14
130.97
127.58
125.79
121.20
119.87
119.27
112.37
86.27
28.15
155
145
135
125
115
105
95
90
85
80
75
70
65
60
55
50
45
40
35
30
25
20
f1 (мд)

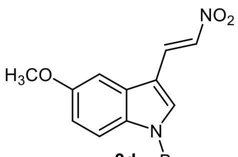

9d

DMSO-d6

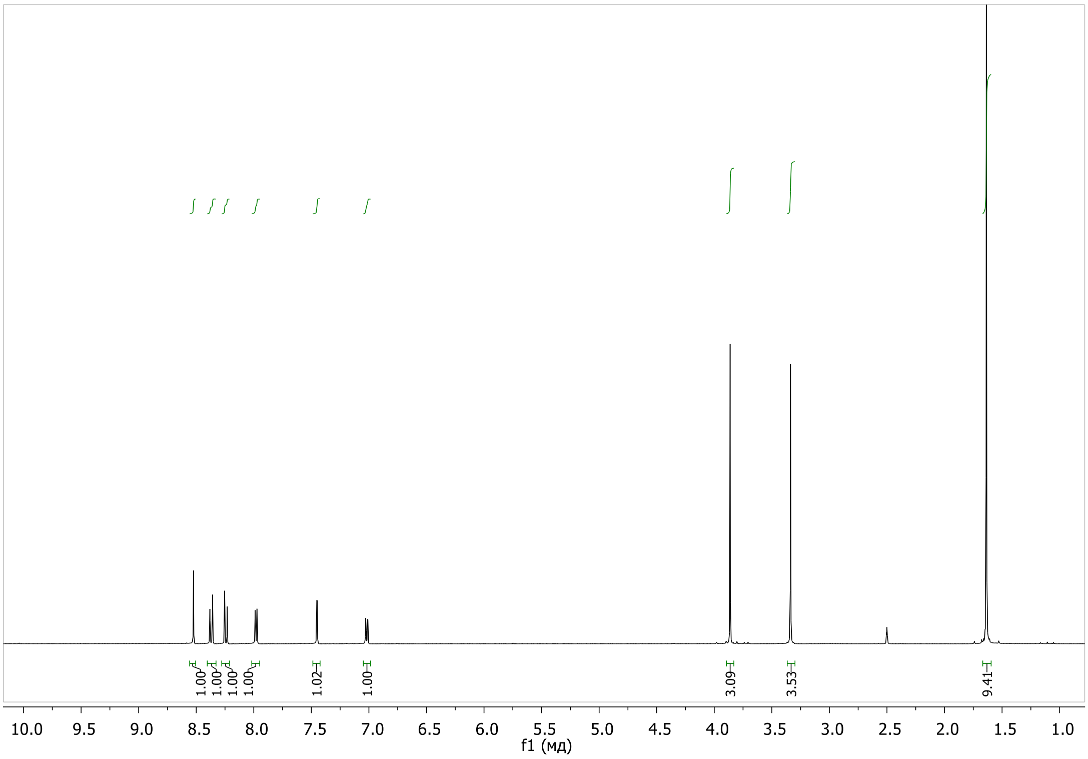

1.00
1.00
1.00
1.00
1.02
1.00
3.09
3.53
9.41
10.0 9.5 9.0 8.5 8.0 7.5 7.0 6.5 6.0 5.5 5.0 4.5 4.0 3.5 3.0 2.5 2.0 1.5 1.0
f1 (мд)

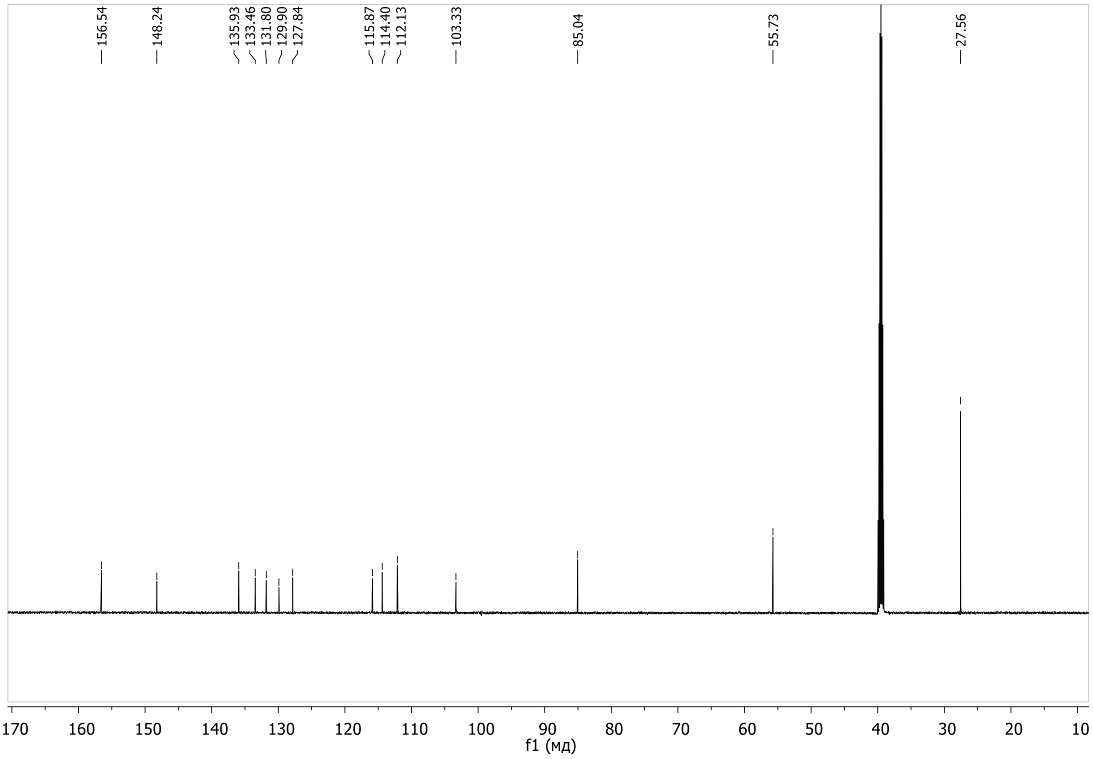

156.54
148.24
135.93
133.46
131.80
129.90
127.84
115.87
114.40
112.13
103.33
85.04
55.73
27.56
170 160 150 140 130 120 110 100 90 80 70 60 50 40 30 20 10
f1 (мд)

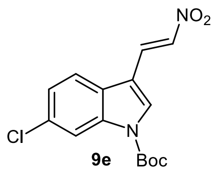
NO2
Cl
N
Boc

9e

DMSO-d6

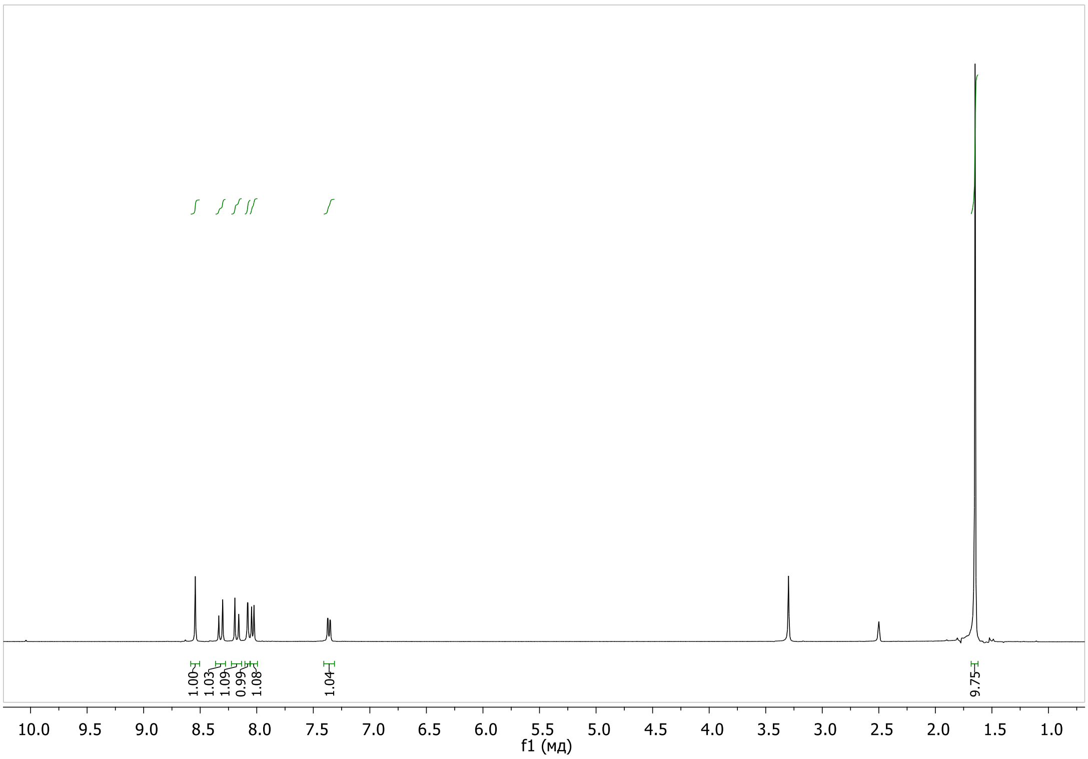
1.00
1.03
1.09
0.99
1.08
1.04
9.75
10.0 9.5 9.0 8.5 8.0 7.5 7.0 6.5 6.0 5.5 5.0 4.5 4.0 3.5 3.0 2.5 2.0 1.5 1.0
f1 (мд)

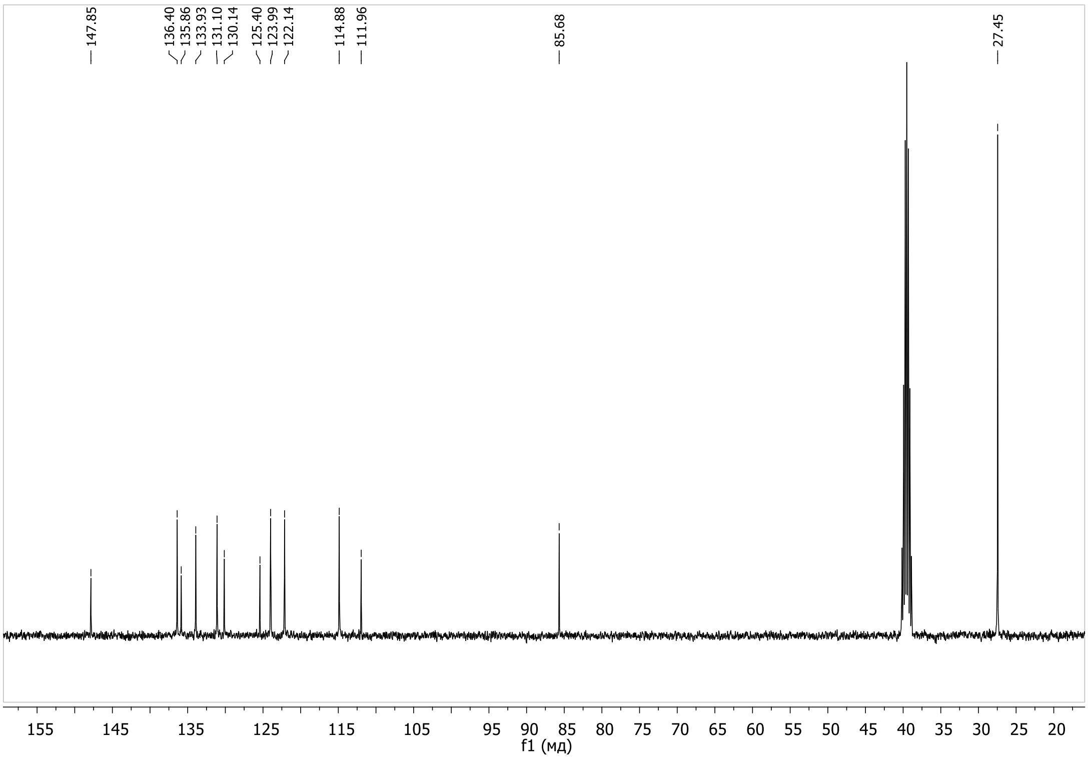
147.85
136.40
135.86
133.93
131.10
130.14
125.40
123.99
122.14
114.88
111.96
85.68
27.45
155 145 135 125 115 105 95 90 85 80 75 70 65 60 55 50 45 40 35 30 25 20
f1 (мд)

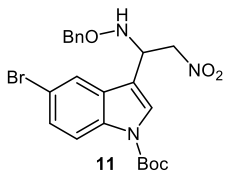
BnO
H
N
NO2
Br
N
Boc

11

CDCl$_3$

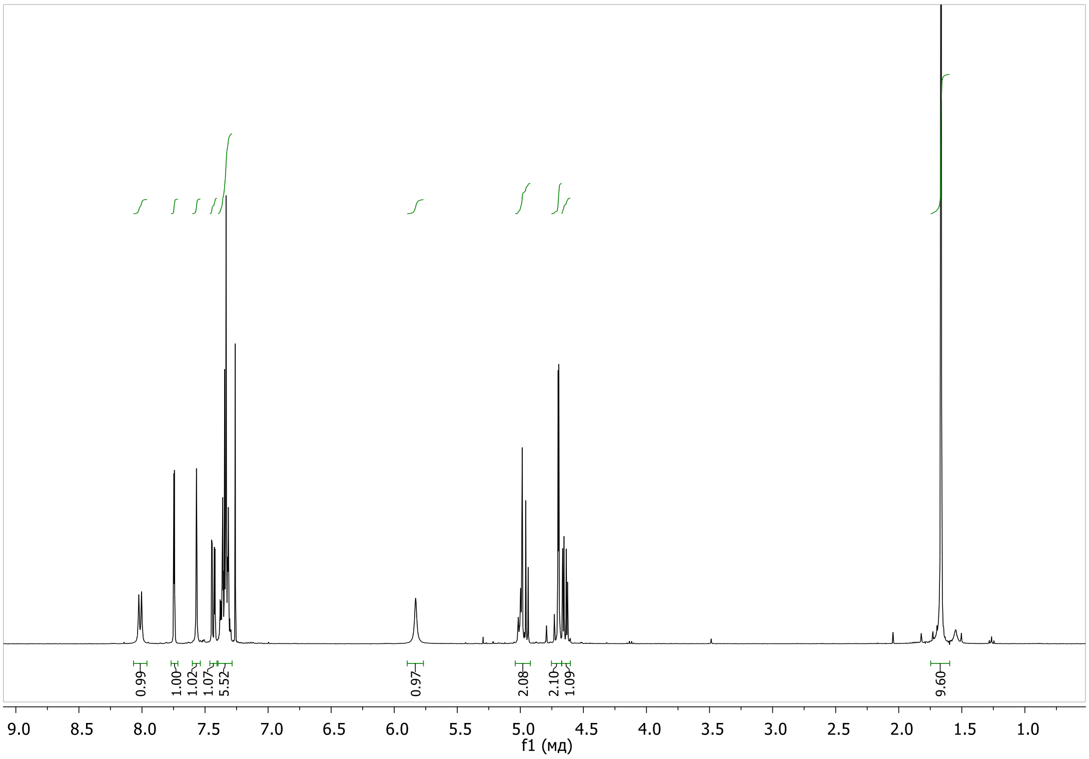
0.99
1.00
1.02
1.07
5.52
0.97
2.08
2.10
1.09
9.60
9.0 8.5 8.0 7.5 7.0 6.5 6.0 5.5 5.0 4.5 4.0 3.5 3.0 2.5 2.0 1.5 1.0
f1 (мд)

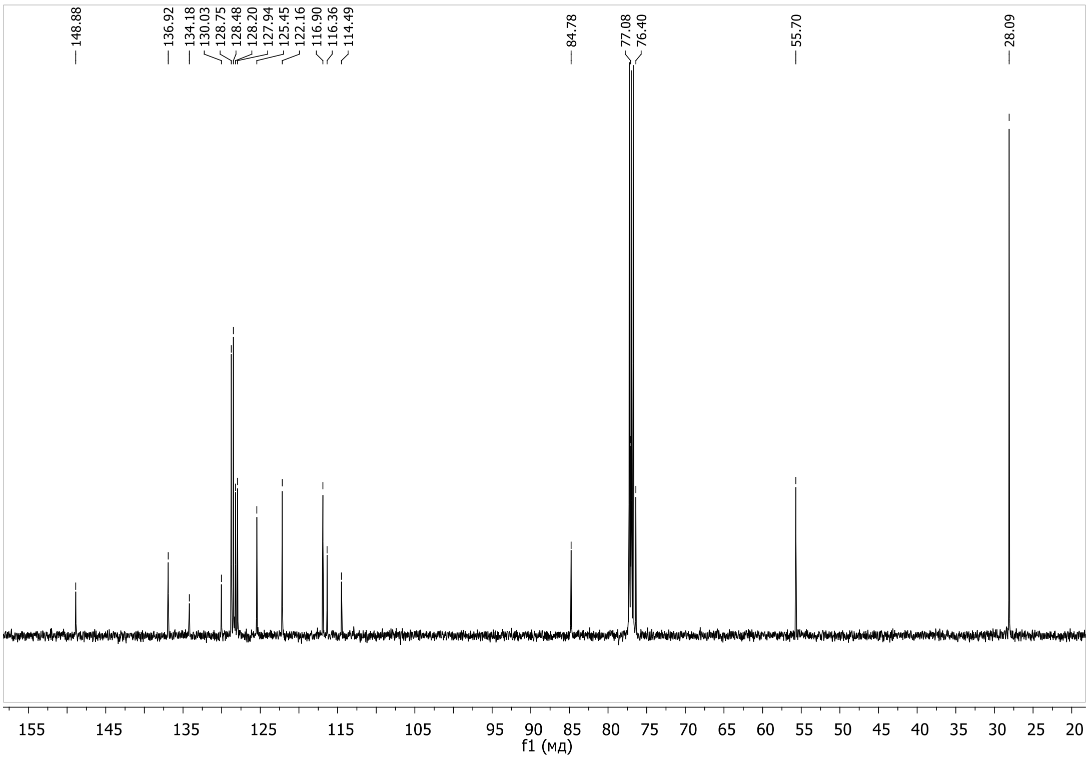
148.88
136.92
134.18
130.03
128.75
128.48
128.20
127.94
125.45
122.16
116.90
116.36
114.49
84.78
77.08
76.40
55.70
28.09
155 145 135 125 115 105 95 90 85 80 75 70 65 60 55 50 45 40 35 30 25 20
f1 (мд)

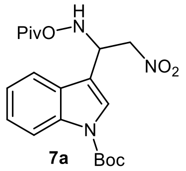
PivO
N
H
NO2
N
Boc

7a

$CDCl_3$

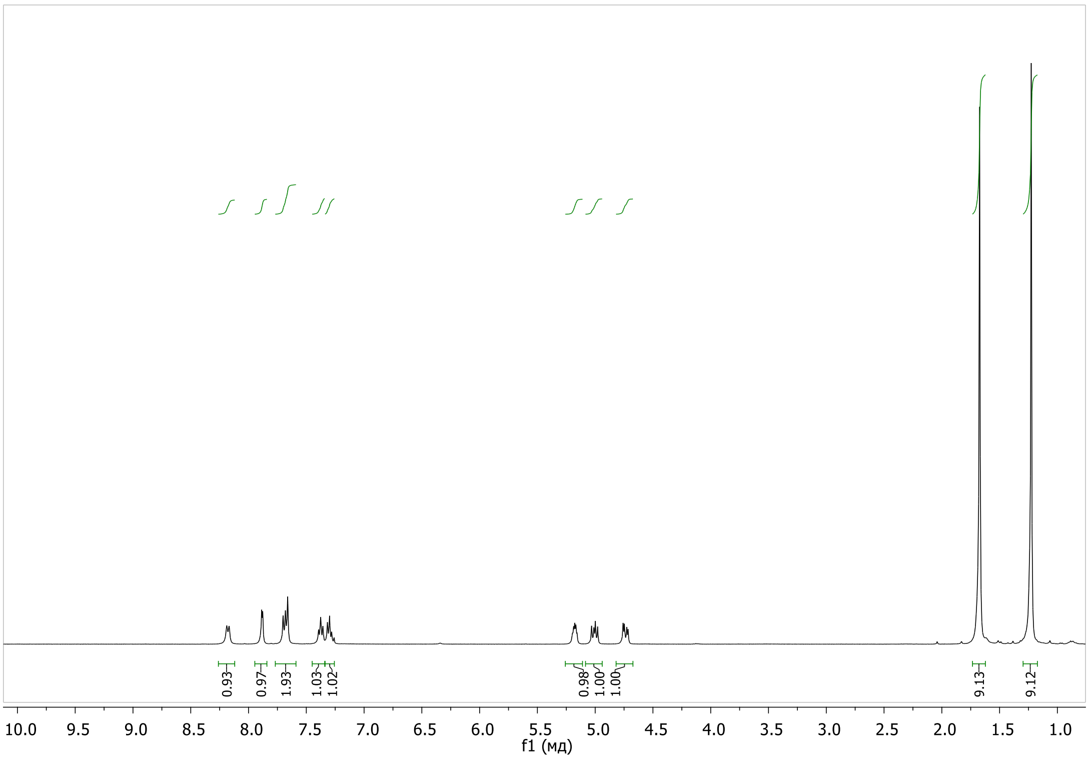
0.93
0.97
1.93
1.03
1.02
0.98
1.00
1.00
9.13
9.12
10.0
9.5
9.0
8.5
8.0
7.5
7.0
6.5
6.0
5.5
5.0
4.5
4.0
3.5
3.0
2.5
2.0
1.5
1.0
f1 (мд)

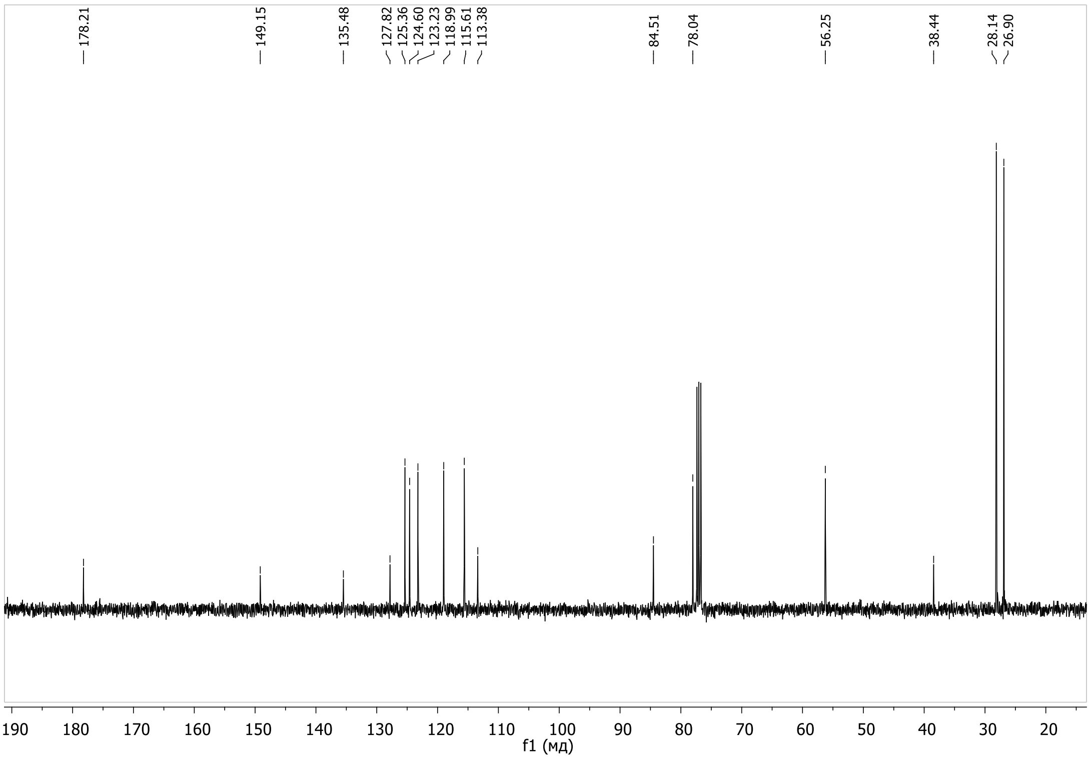
178.21
149.15
135.48
127.82
125.36
124.60
123.23
118.99
115.61
113.38
84.51
78.04
56.25
38.44
28.14
26.90
190
180
170
160
150
140
130
120
110
100
90
80
70
60
50
40
30
20
f1 (мд)

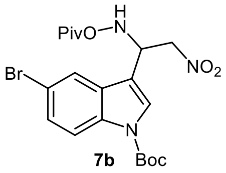
PivO
H
N
NO2
Br
N
Boc

7b

$CDCl_3$

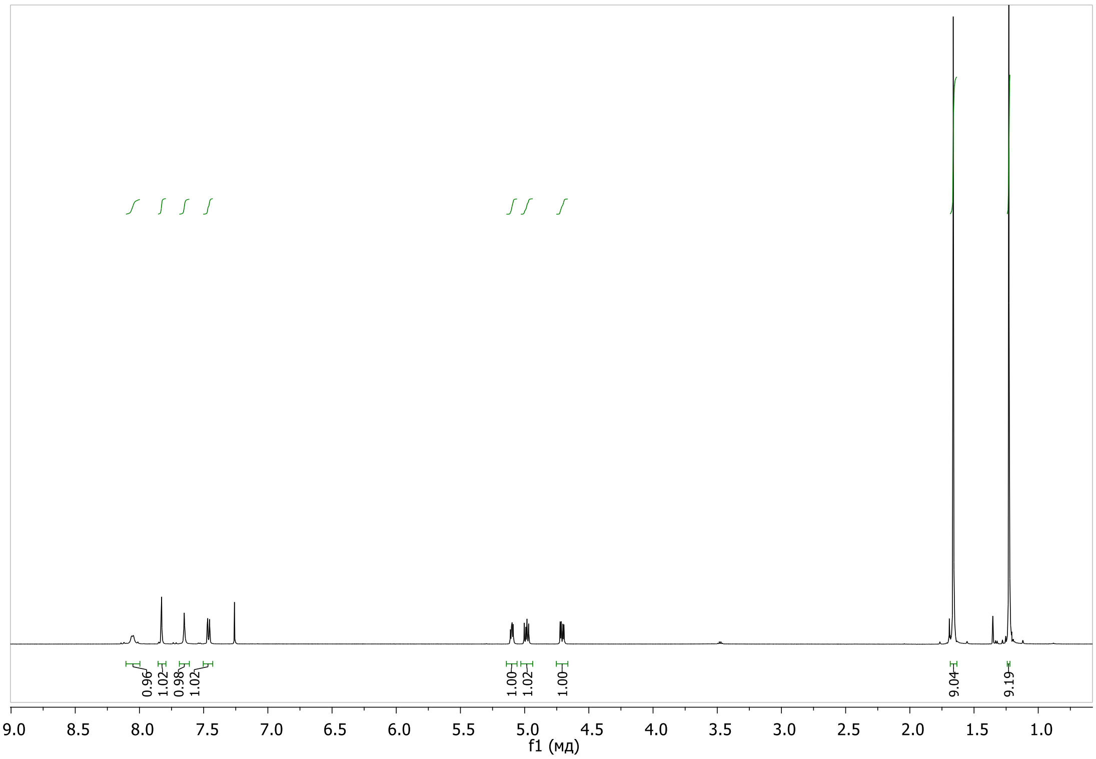
0.96
1.02
0.98
1.02
1.00
1.02
1.00
9.04
9.19
9.0 8.5 8.0 7.5 7.0 6.5 6.0 5.5 5.0 4.5 4.0 3.5 3.0 2.5 2.0 1.5 1.0
f1 (мд)

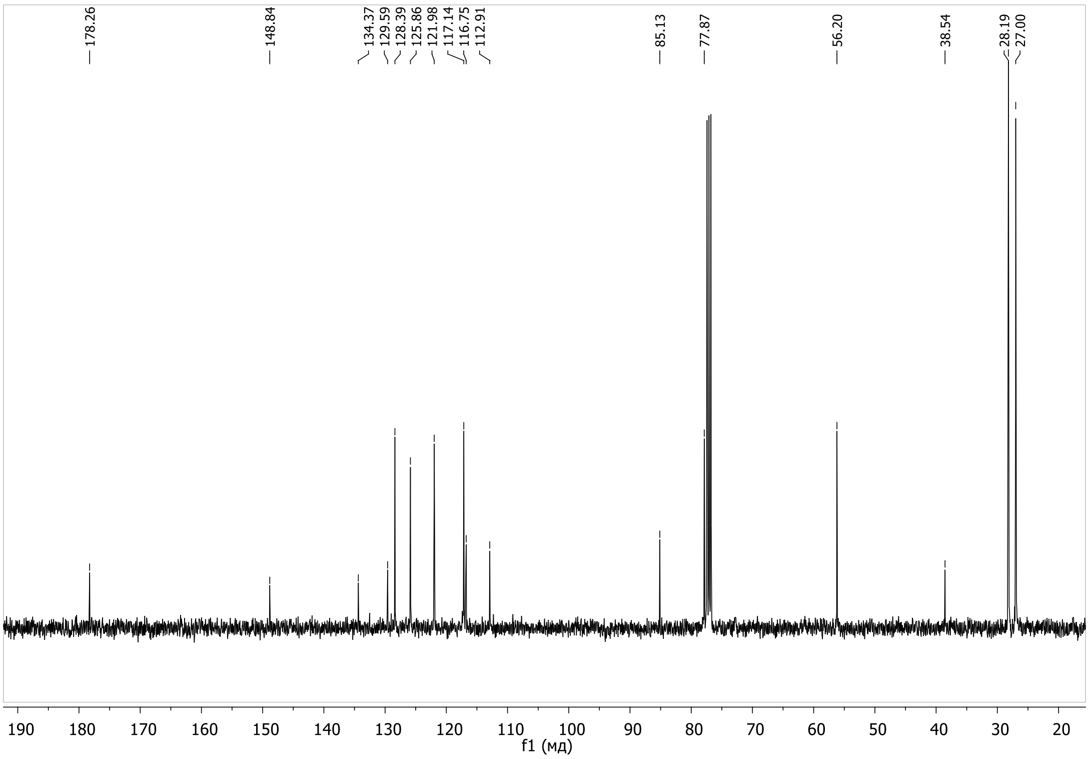
178.26
148.84
134.37
129.59
128.39
125.86
121.98
117.14
116.75
112.91
85.13
77.87
56.20
38.54
28.19
27.00
190 180 170 160 150 140 130 120 110 100 90 80 70 60 50 40 30 20
f1 (мд)

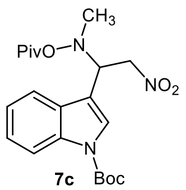

7c

$CDCl_3$

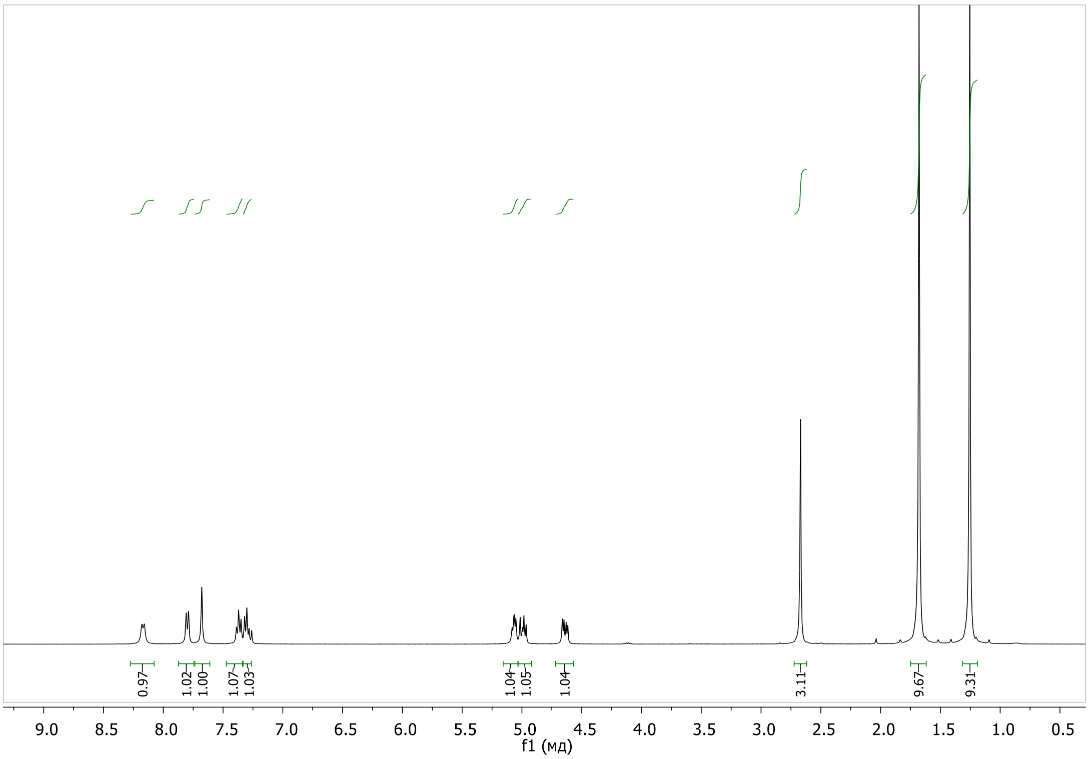
0.97
1.02
1.00
1.07
1.03
1.04
1.05
1.04
3.11
9.67
9.31
9.0 8.5 8.0 7.5 7.0 6.5 6.0 5.5 5.0 4.5 4.0 3.5 3.0 2.5 2.0 1.5 1.0 0.5
f1 (мд)

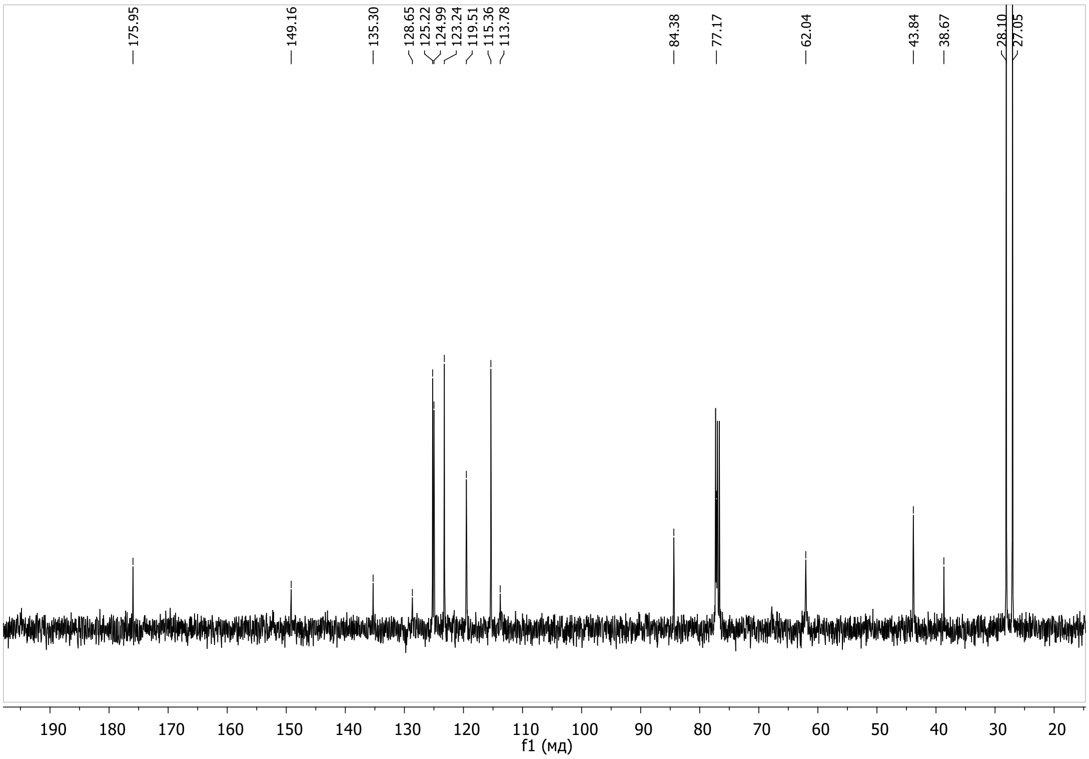
175.95
149.16
135.30
128.65
125.22
124.99
123.24
119.51
115.36
113.78
84.38
77.17
62.04
43.84
38.67
28.10
27.05
190 180 170 160 150 140 130 120 110 100 90 80 70 60 50 40 30 20
f1 (мд)

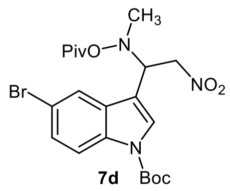
CH3
PivO
N
NO2
Br
N
7d
Boc

CDCl$_3$

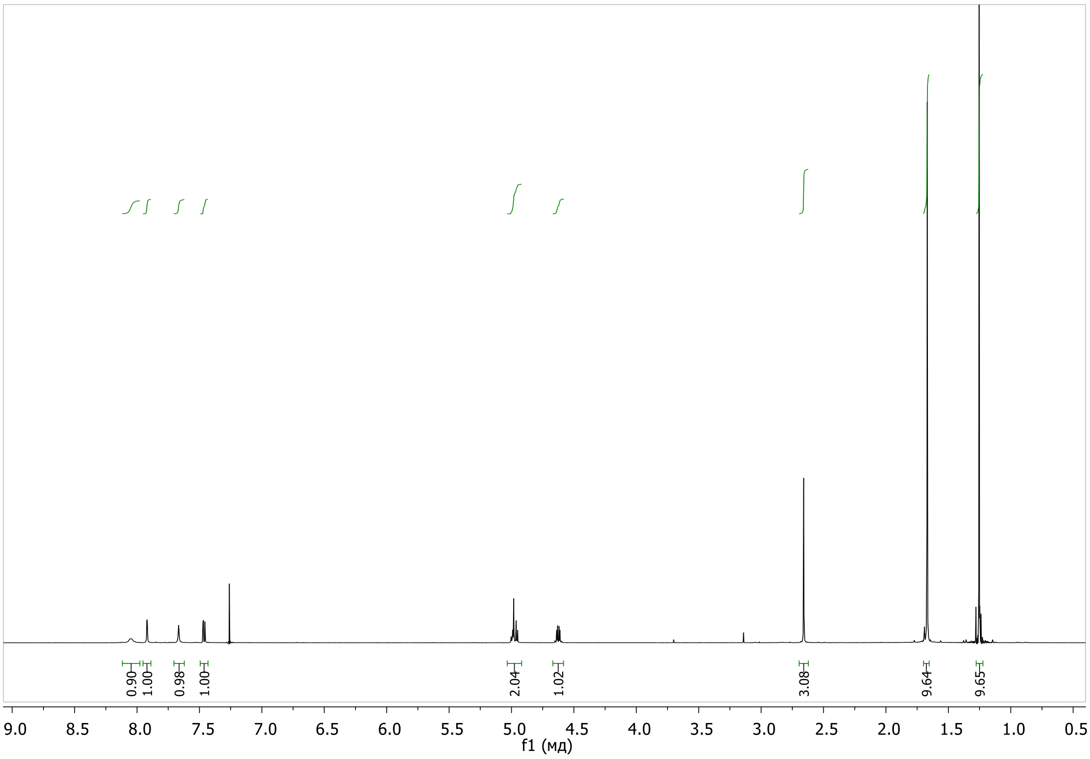
0.90
1.00
0.98
1.00
2.04
1.02
3.08
9.64
9.65
9.0 8.5 8.0 7.5 7.0 6.5 6.0 5.5 5.0 4.5 4.0 3.5 3.0 2.5 2.0 1.5 1.0 0.5
f1 (мд)

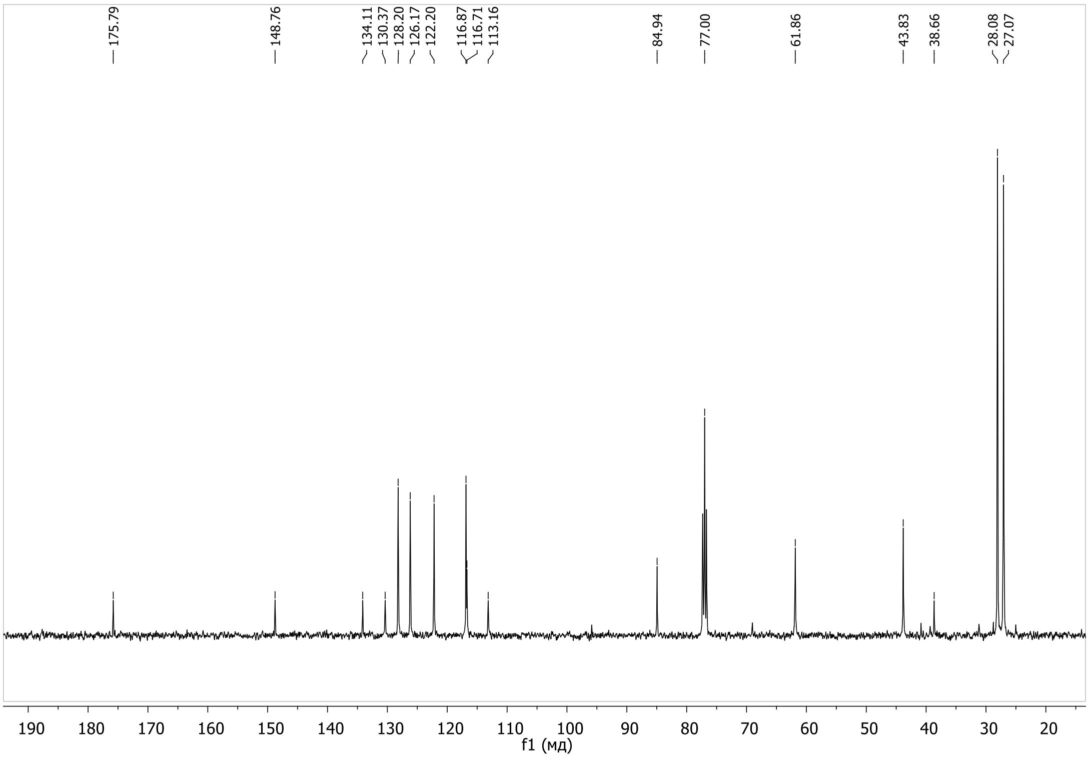
175.79
148.76
134.11
130.37
128.20
126.17
122.20
116.87
116.71
113.16
84.94
77.00
61.86
43.83
38.66
28.08
27.07
190 180 170 160 150 140 130 120 110 100 90 80 70 60 50 40 30 20
f1 (мд)

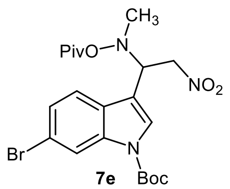
PivO
N
CH3
NO2
Br
N
Boc

7e

$CDCl_3$

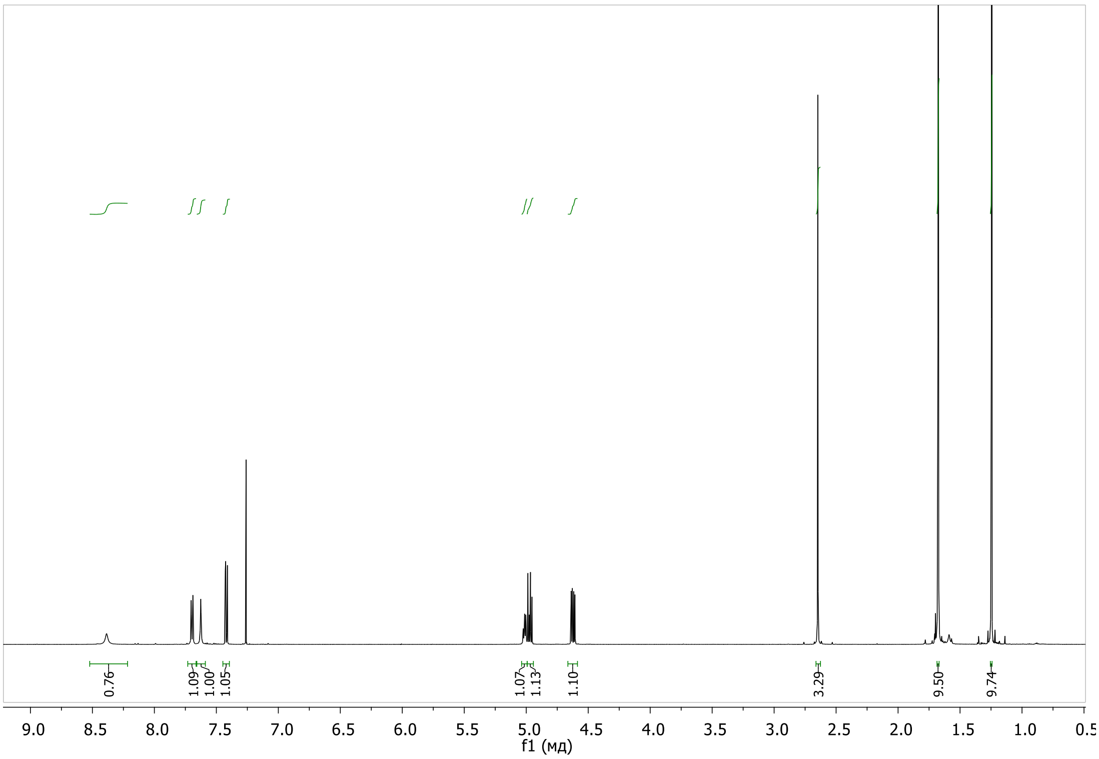
0.76
1.09
1.00
1.05
1.07
1.13
1.10
3.29
9.50
9.74
9.0
8.5
8.0
7.5
7.0
6.5
6.0
5.5
5.0
4.5
4.0
3.5
3.0
2.5
2.0
1.5
1.0
f1 (мд)

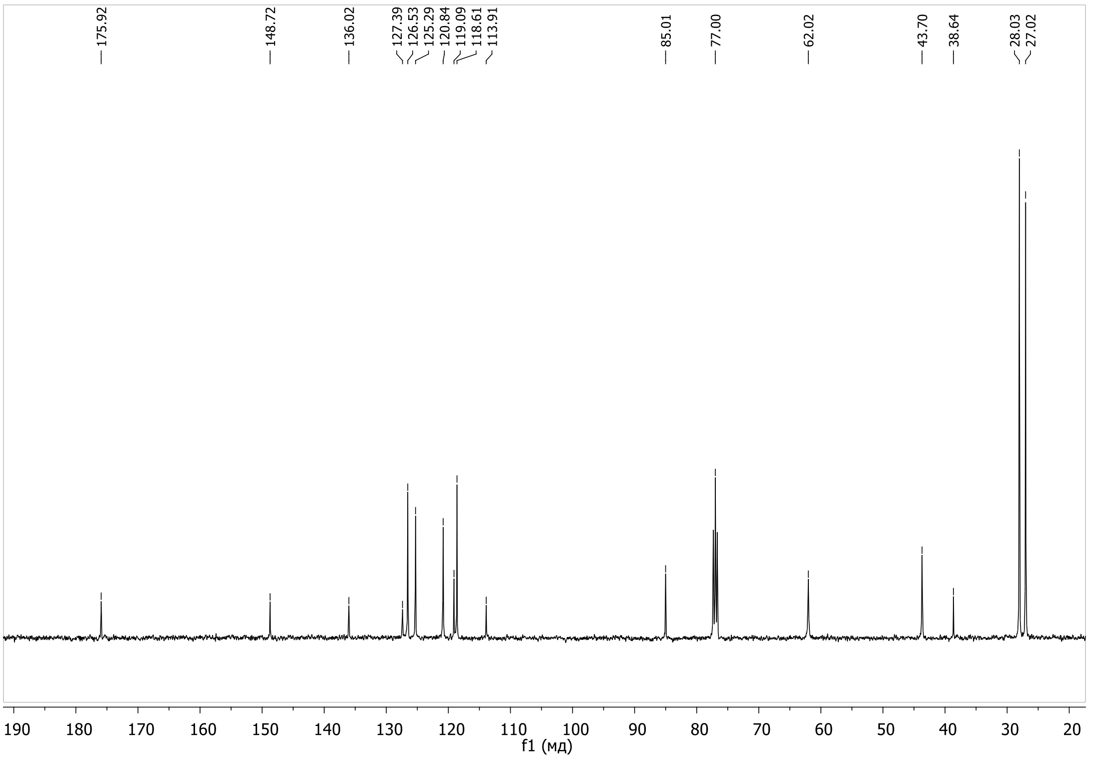
175.92
148.72
136.02
127.39
126.53
125.29
120.84
119.09
118.61
113.91
85.01
77.00
62.02
43.70
38.64
28.03
27.02
190
180
170
160
150
140
130
120
110
100
90
80
70
60
50
40
30
20
f1 (мд)

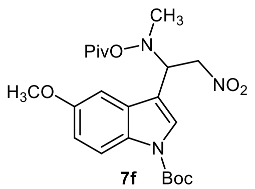

7f

CDCl$_3$

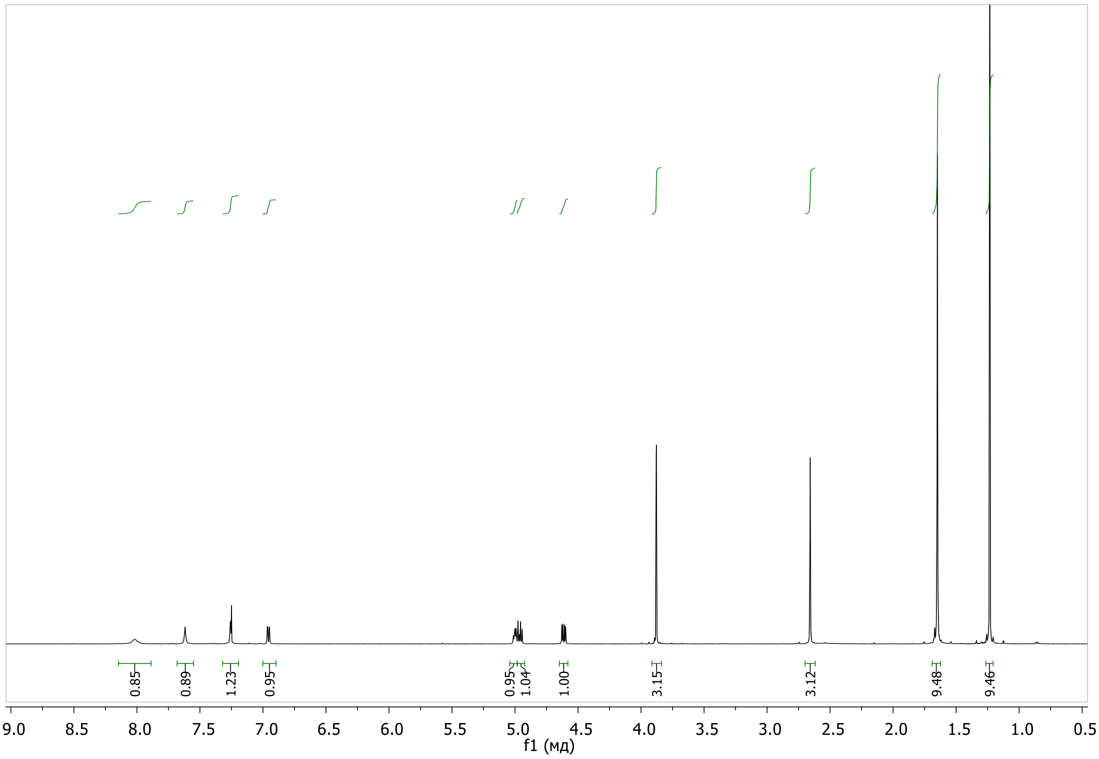
0.85
0.89
1.23
0.95
0.95
1.04
1.00
3.15
3.12
9.48
9.46
9.0 8.5 8.0 7.5 7.0 6.5 6.0 5.5 5.0 4.5 4.0 3.5 3.0 2.5 2.0 1.5 1.0 0.5
f1 (мд)

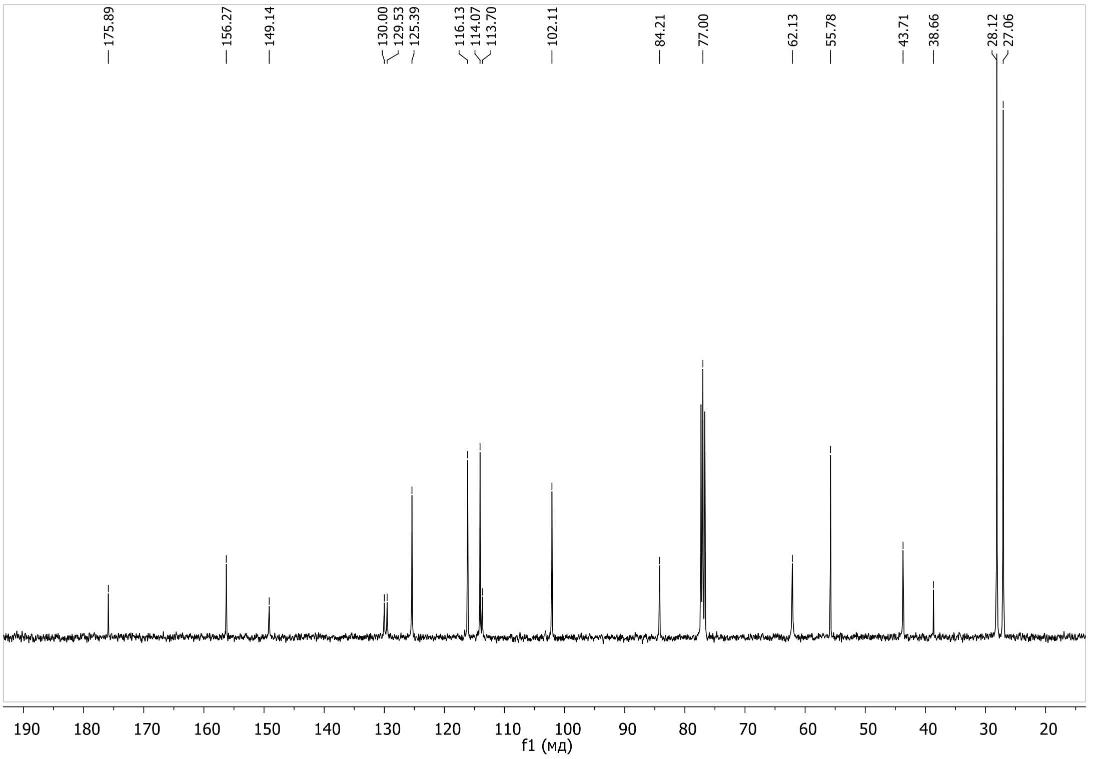
175.89
156.27
149.14
130.00
129.53
125.39
116.13
114.07
113.70
102.11
84.21
77.00
62.13
55.78
43.71
38.66
28.12
27.06
190 180 170 160 150 140 130 120 110 100 90 80 70 60 50 40 30 20
f1 (мд)

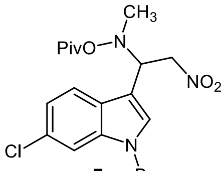

CH3
PivO
N
NO2
Cl
N
Boc

7g

CDCl$_3$

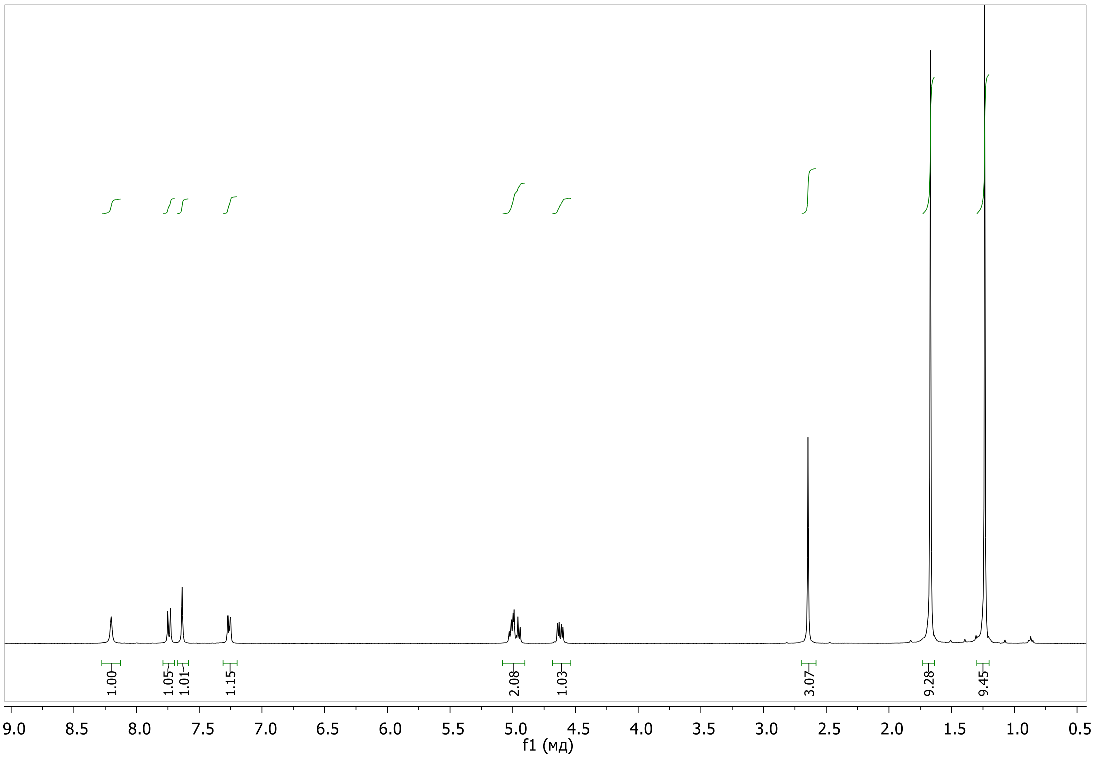

1.00
1.05
1.01
1.15
2.08
1.03
3.07
9.28
9.45
9.0
8.5
8.0
7.5
7.0
6.5
6.0
5.5
5.0
4.5
4.0
3.5
3.0
2.5
2.0
1.5
1.0
0.5
f1 (мд)

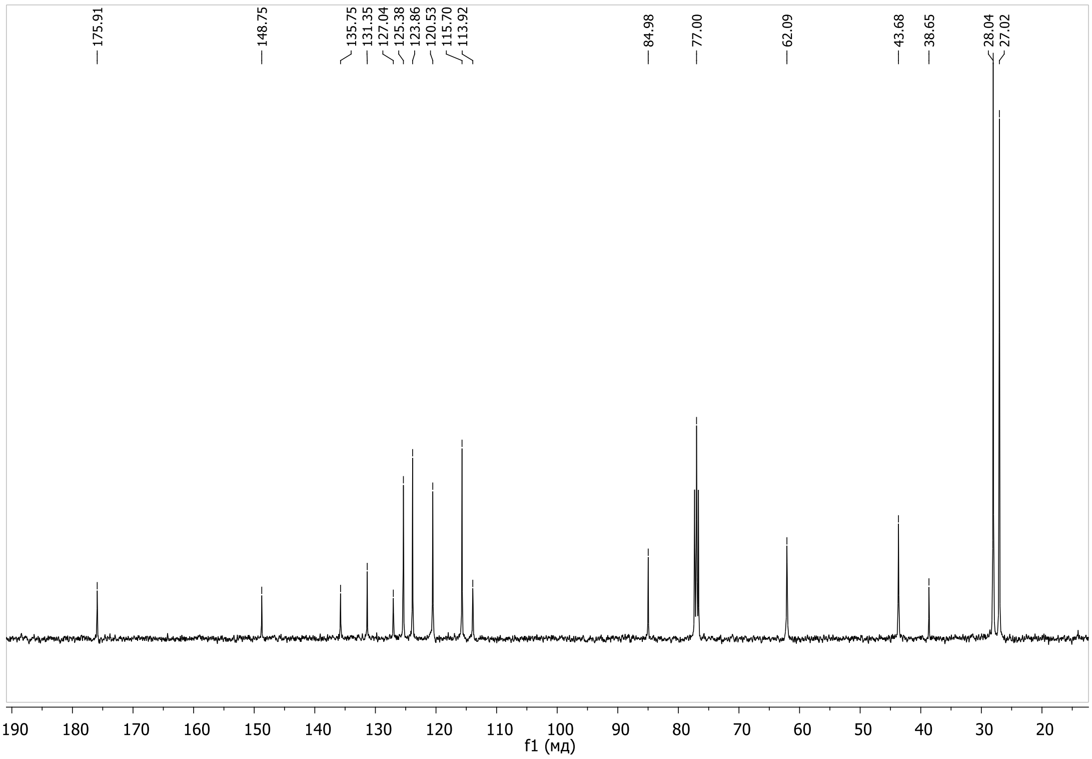

175.91
148.75
135.75
131.35
127.04
125.38
123.86
120.53
115.70
113.92
84.98
77.00
62.09
43.68
38.65
28.04
27.02
190
180
170
160
150
140
130
120
110
100
90
80
70
60
50
40
30
20
f1 (мд)

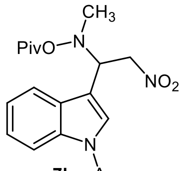
CH3
PivO
N
NO2
N
Ac

7h

$CDCl_3$

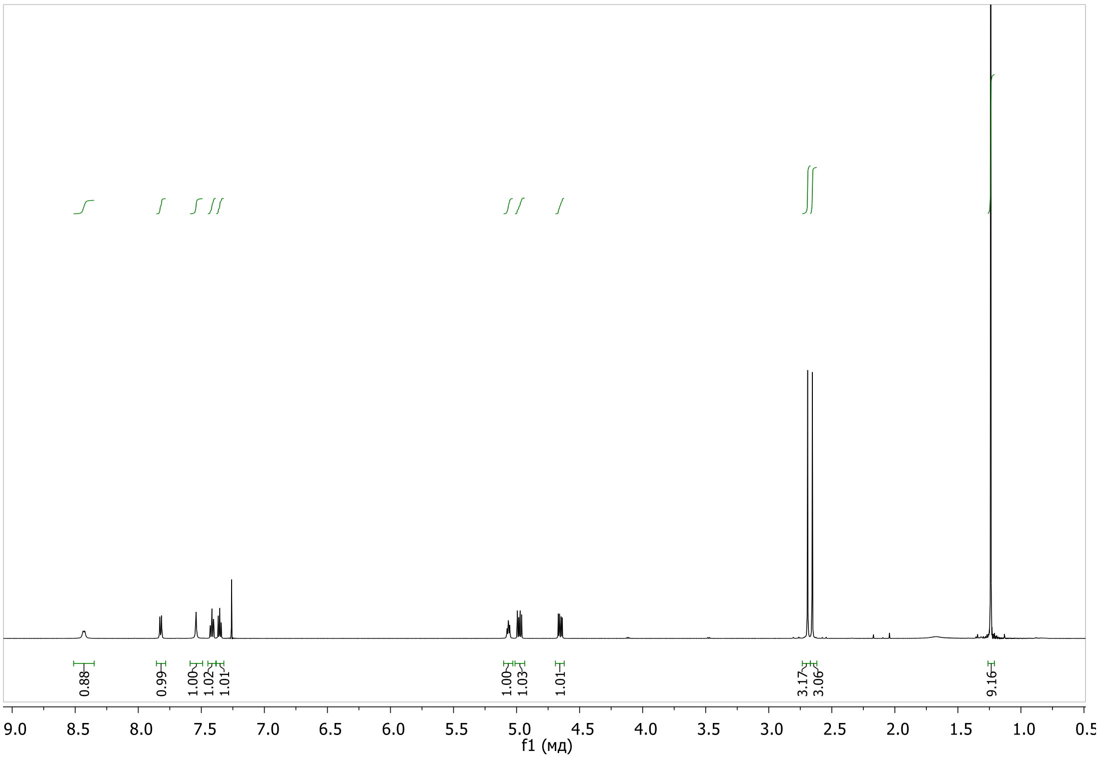
0.88
0.99
1.00
1.02
1.01
1.00
1.03
1.01
3.17
3.06
9.16
9.0 8.5 8.0 7.5 7.0 6.5 6.0 5.5 5.0 4.5 4.0 3.5 3.0 2.5 2.0 1.5 1.0 0.5
f1 (мд)

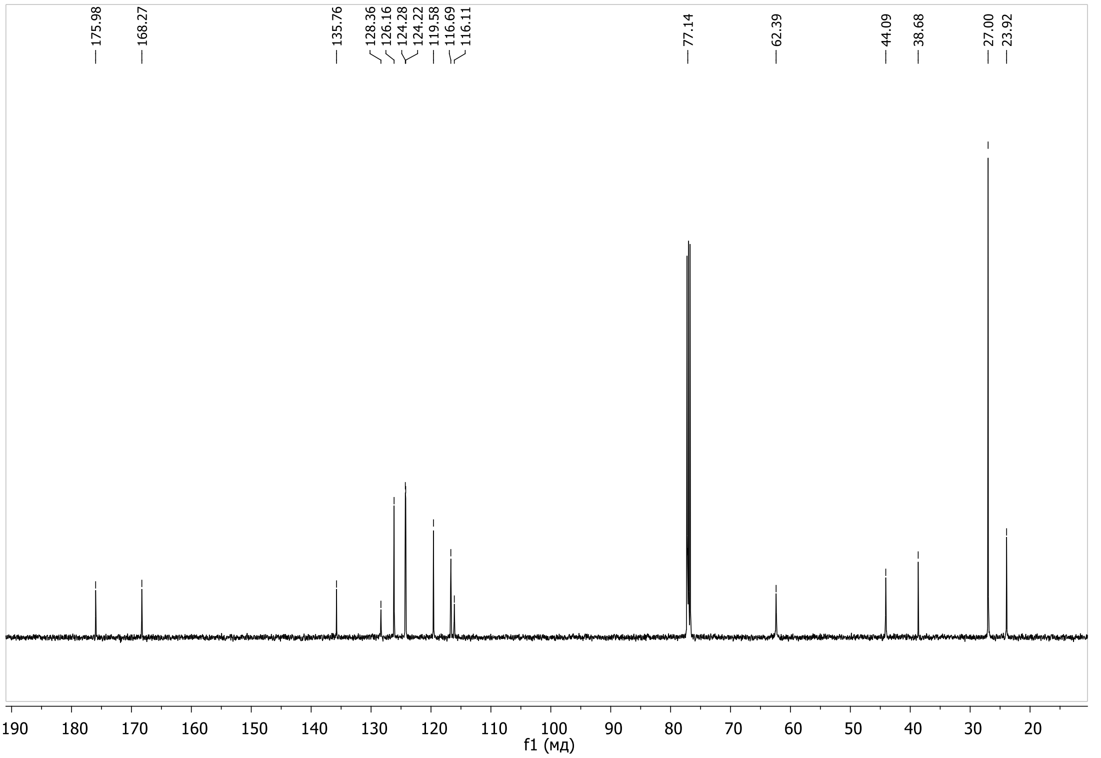
175.98
168.27
135.76
128.36
126.16
124.28
124.22
119.58
116.69
116.11
77.14
62.39
44.09
38.68
27.00
23.92
190 180 170 160 150 140 130 120 110 100 90 80 70 60 50 40 30 20
f1 (мд)

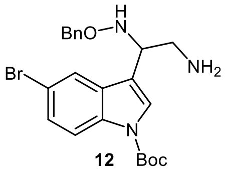
H
BnO
N
NH2
Br
N
12
Boc

$CDCl_3$

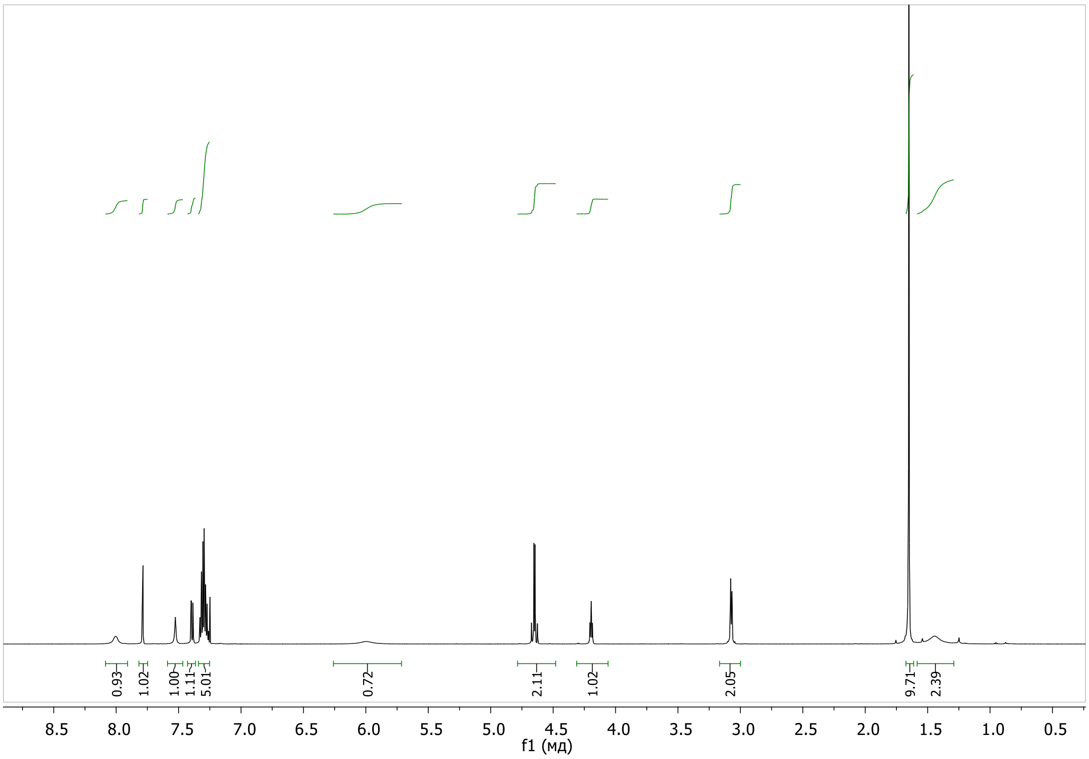
0.93
1.02
1.00
1.11
5.01
0.72
2.11
1.02
2.05
9.71
2.39
8.5
8.0
7.5
7.0
6.5
6.0
5.5
5.0
4.5
4.0
3.5
3.0
2.5
2.0
1.5
1.0
0.5
f1 (мд)

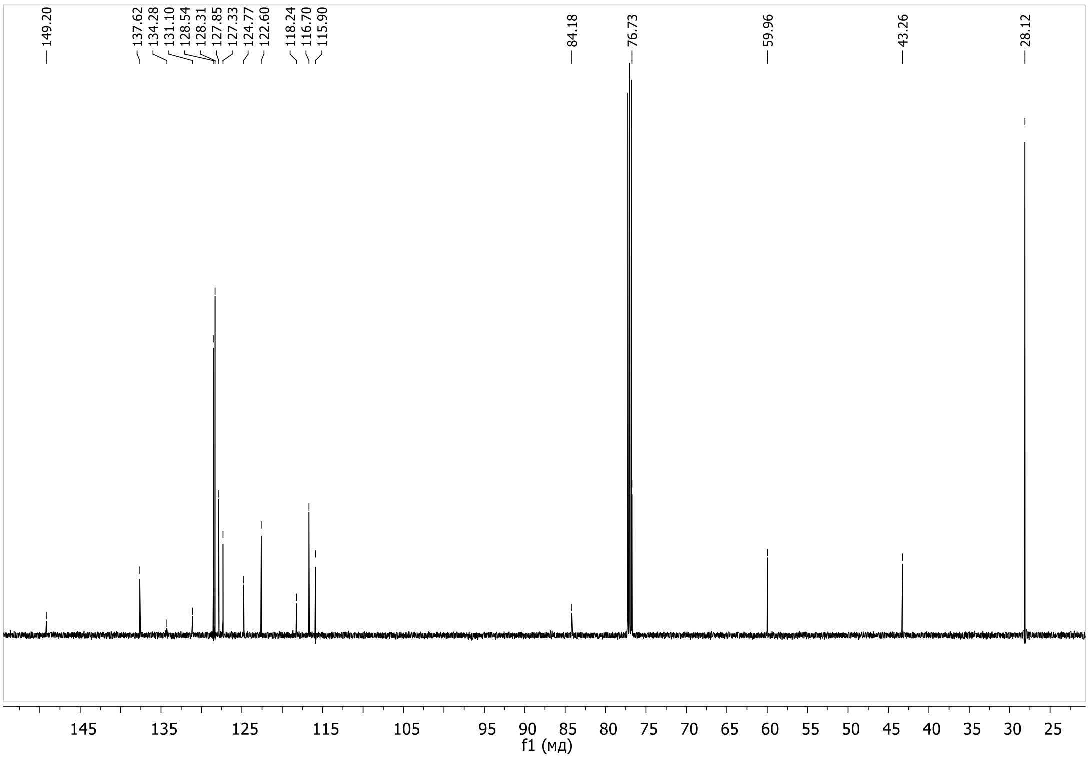
149.20
137.62
134.28
131.10
128.54
128.31
127.85
127.33
124.77
122.60
118.24
116.70
115.90
84.18
76.73
59.96
43.26
28.12
145
135
125
115
105
95
90
85
80
75
70
65
60
55
50
45
40
35
30
25
f1 (мд)

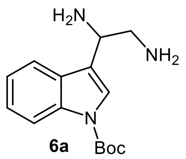
H2N
NH2
N
Boc

6a

$CDCl_3$

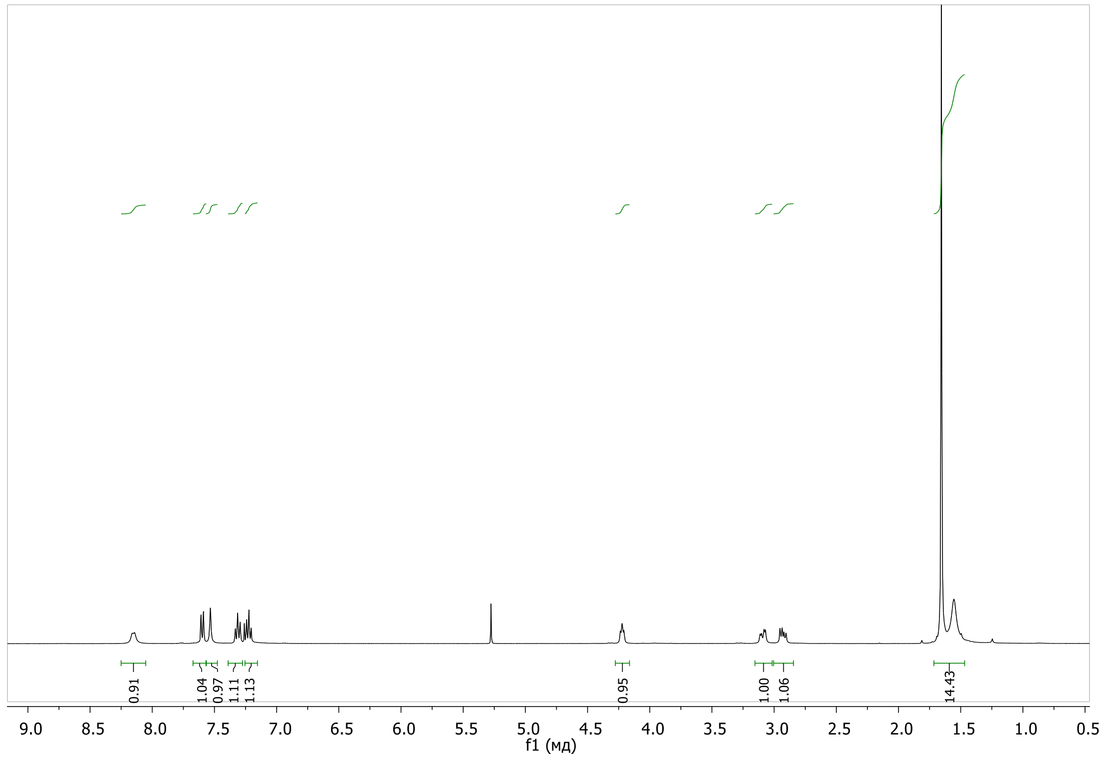
0.91
1.04
0.97
1.11
1.13
0.95
1.00
1.06
14.43
9.0
8.5
8.0
7.5
7.0
6.5
6.0
5.5
5.0
4.5
4.0
3.5
3.0
2.5
2.0
1.5
1.0
0.5
f1 (мд)

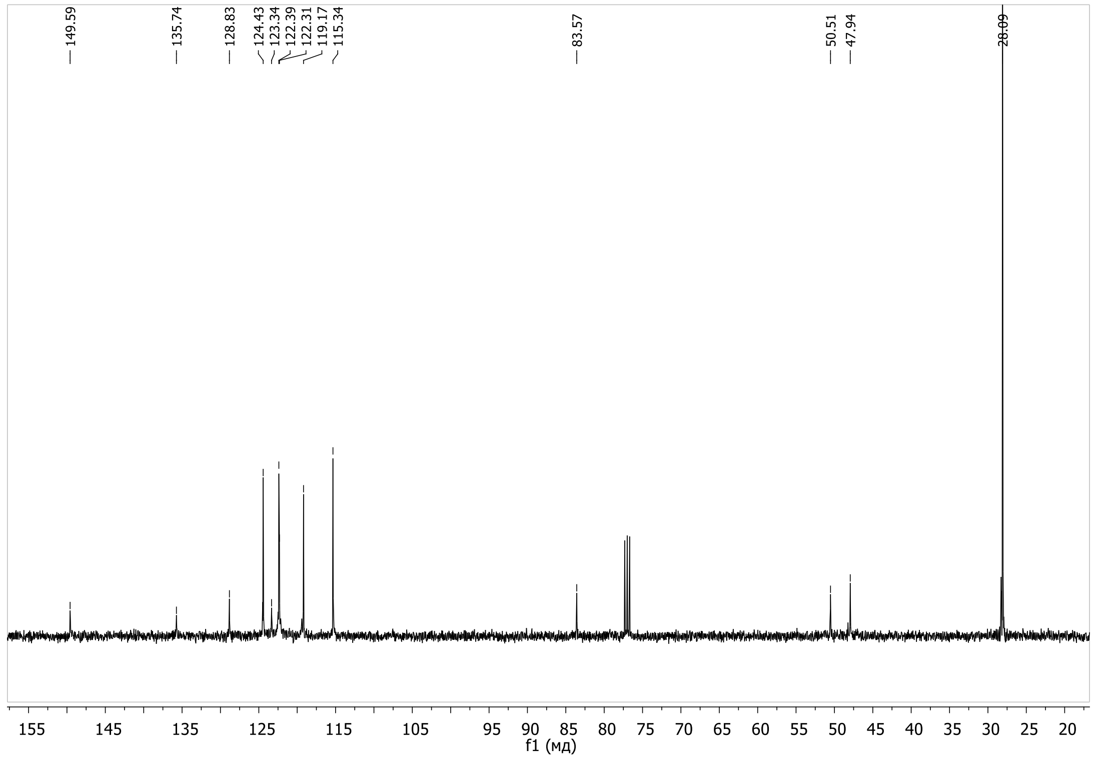
149.59
135.74
128.83
124.43
123.34
122.39
122.31
119.17
115.34
83.57
50.51
47.94
28.09
155
145
135
125
115
105
95
90
85
80
75
70
65
60
55
50
45
40
35
30
25
20
f1 (мд)

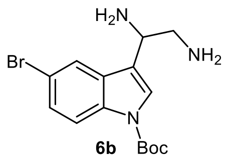
H2N
NH2
Br
N
Boc

6b

CDCl$_3$

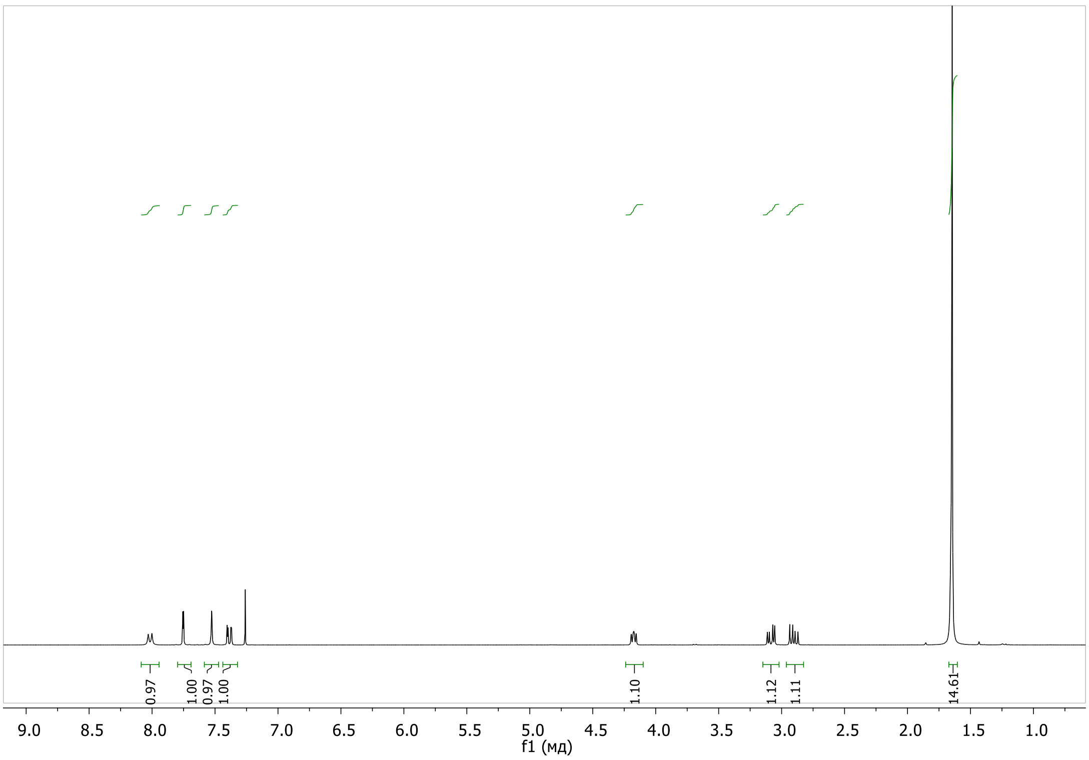
0.97
1.00
0.97
1.00
1.10
1.12
1.11
14.61
9.0 8.5 8.0 7.5 7.0 6.5 6.0 5.5 5.0 4.5 4.0 3.5 3.0 2.5 2.0 1.5 1.0
f1 (мд)

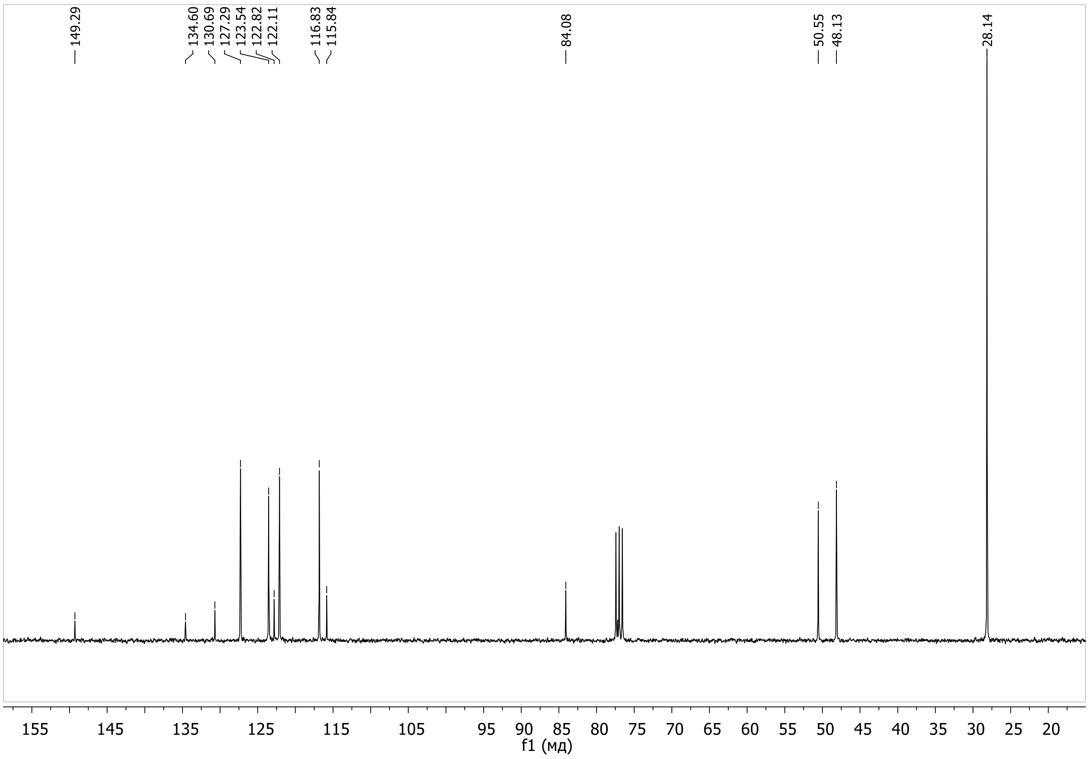
149.29
134.60
130.69
127.29
123.54
122.82
122.11
116.83
115.84
84.08
50.55
48.13
28.14
155 145 135 125 115 105 95 90 85 80 75 70 65 60 55 50 45 40 35 30 25 20
f1 (мд)

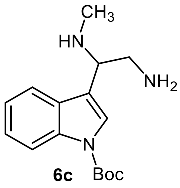

CH3
HN
NH2
N
Boc

6c

$CDCl_3$

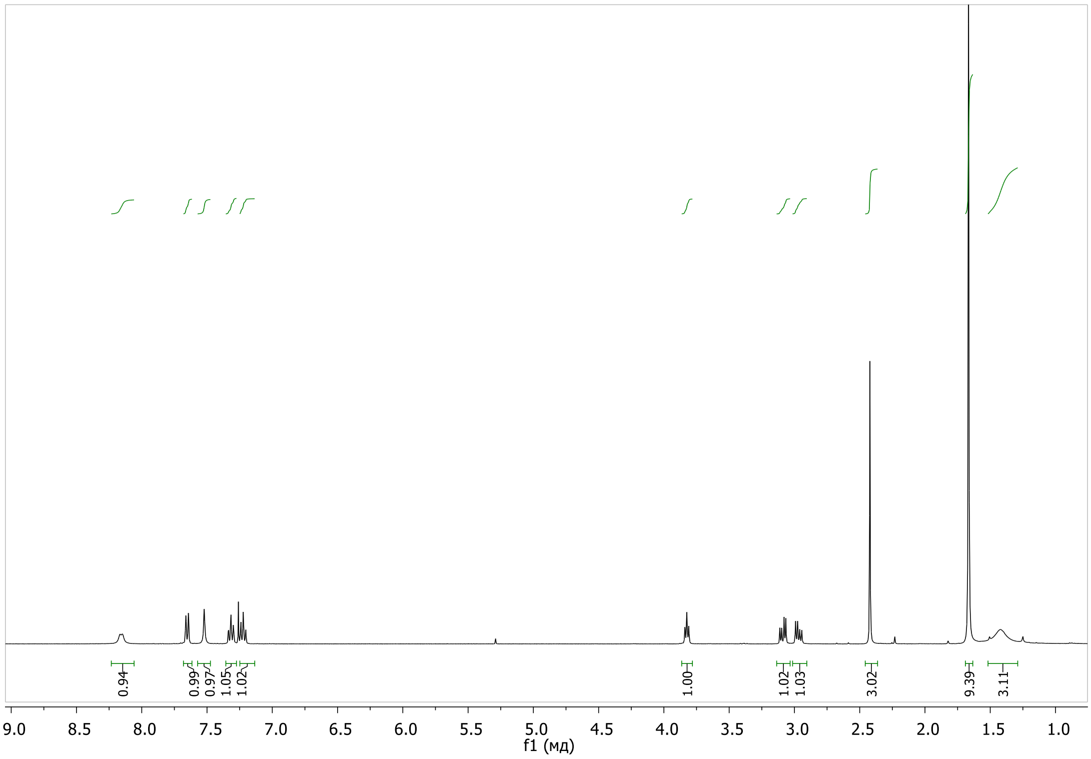

0.94
0.99
0.97
1.05
1.02
1.00
1.02
1.03
3.02
9.39
3.11
9.0 8.5 8.0 7.5 7.0 6.5 6.0 5.5 5.0 4.5 4.0 3.5 3.0 2.5 2.0 1.5 1.0
f1 (мд)

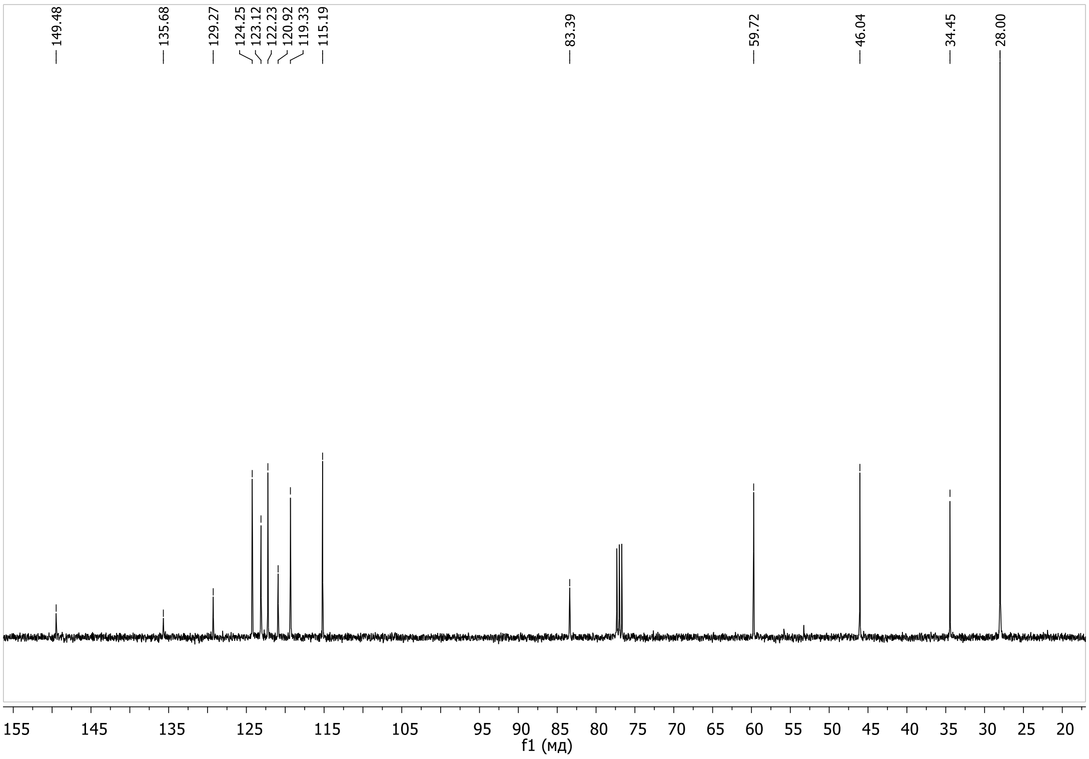

149.48
135.68
129.27
124.25
123.12
122.23
120.92
119.33
115.19
83.39
59.72
46.04
34.45
28.00
155 145 135 125 115 105 95 90 85 80 75 70 65 60 55 50 45 40 35 30 25 20
f1 (мд)

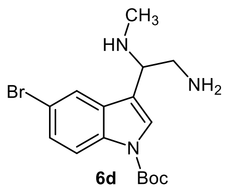

6d

$CDCl_3$

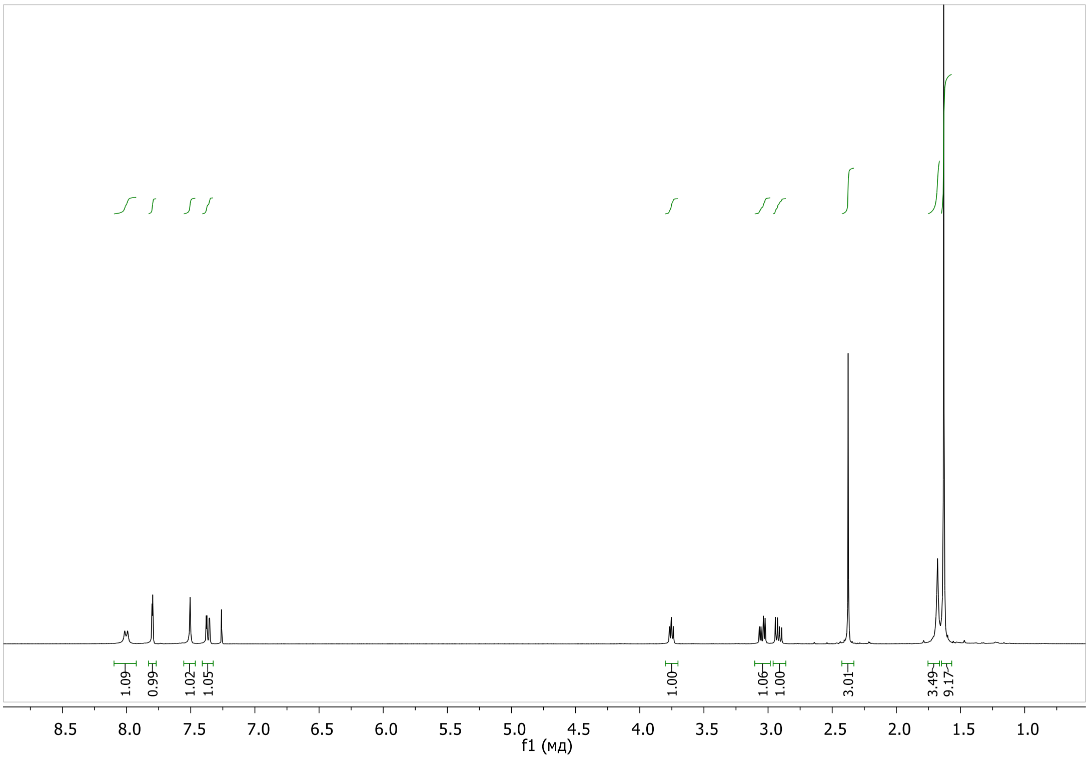

1.09
0.99
1.02
1.05
1.00
1.06
1.00
3.01
3.49
9.17
8.5 8.0 7.5 7.0 6.5 6.0 5.5 5.0 4.5 4.0 3.5 3.0 2.5 2.0 1.5 1.0
f1 (мд)

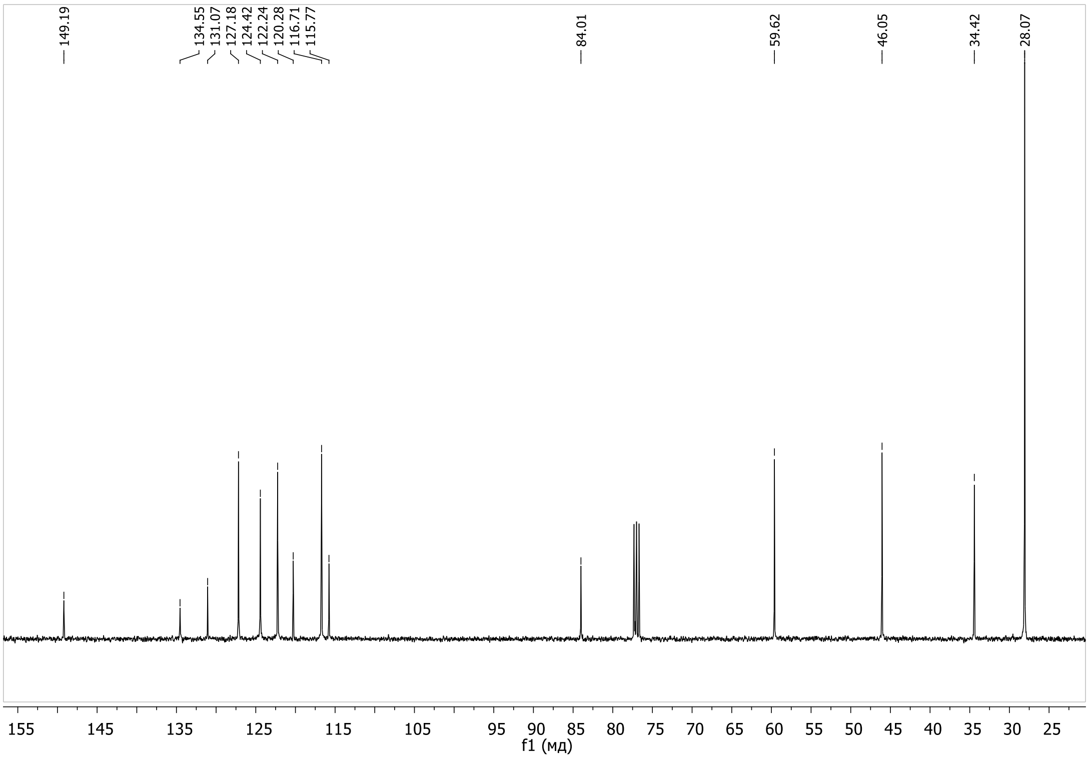

149.19
134.55
131.07
127.18
124.42
122.24
120.28
116.71
115.77
84.01
59.62
46.05
34.42
28.07
155 145 135 125 115 105 95 90 85 80 75 70 65 60 55 50 45 40 35 30 25
f1 (мд)

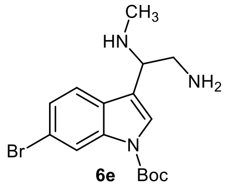
CH3
HN
NH2
Br
N
Boc

6e

$CDCl_3$

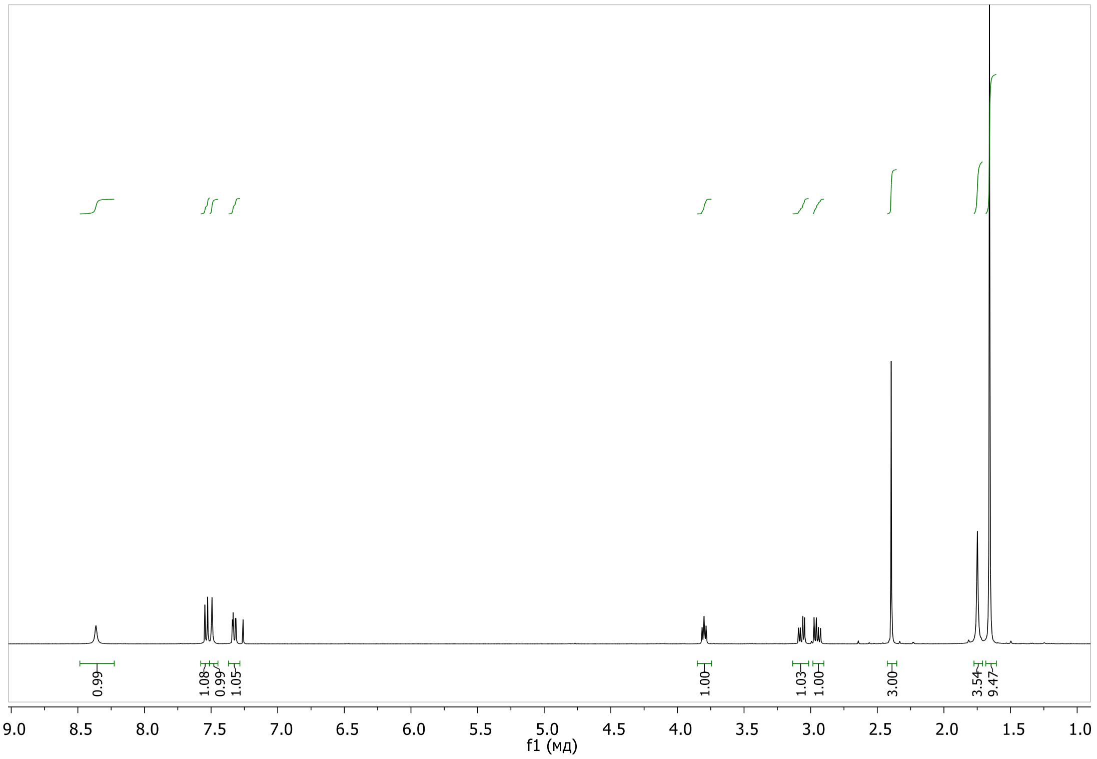
0.99
1.08
0.99
1.05
1.00
1.03
1.00
3.00
3.54
9.47
9.0 8.5 8.0 7.5 7.0 6.5 6.0 5.5 5.0 4.5 4.0 3.5 3.0 2.5 2.0 1.5 1.0
f1 (мд)

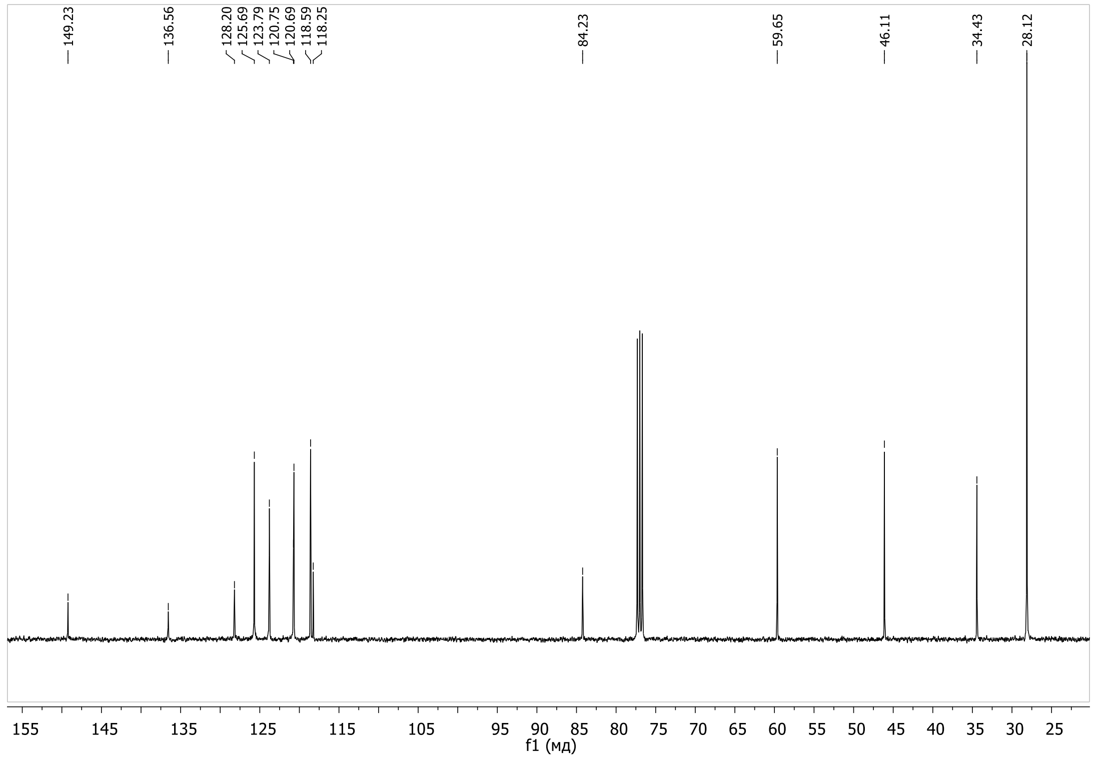
149.23
136.56
128.20
125.69
123.79
120.75
120.69
118.59
118.25
84.23
59.65
46.11
34.43
28.12
155 145 135 125 115 105 95 90 85 80 75 70 65 60 55 50 45 40 35 30 25
f1 (мд)

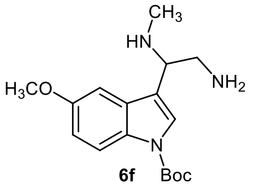

6f

$CDCl_3$

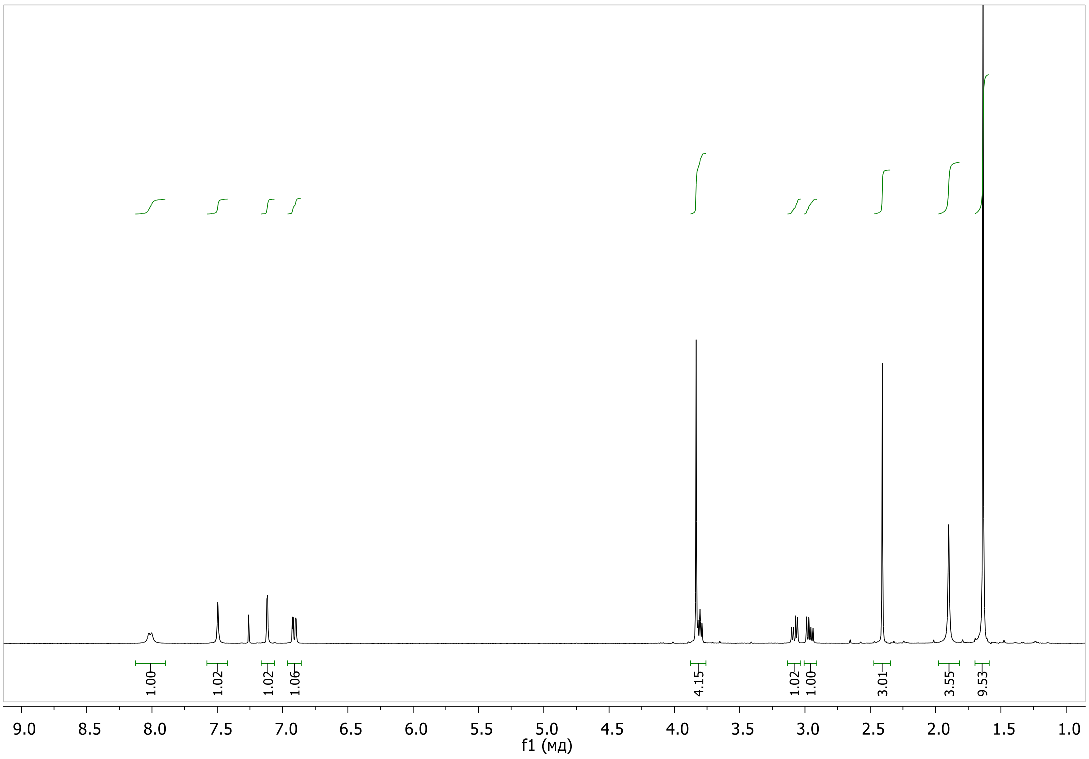

1.00
1.02
1.02
1.06
4.15
1.02
1.00
3.01
3.55
9.53
9.0 8.5 8.0 7.5 7.0 6.5 6.0 5.5 5.0 4.5 4.0 3.5 3.0 2.5 2.0 1.5 1.0
f1 (мд)

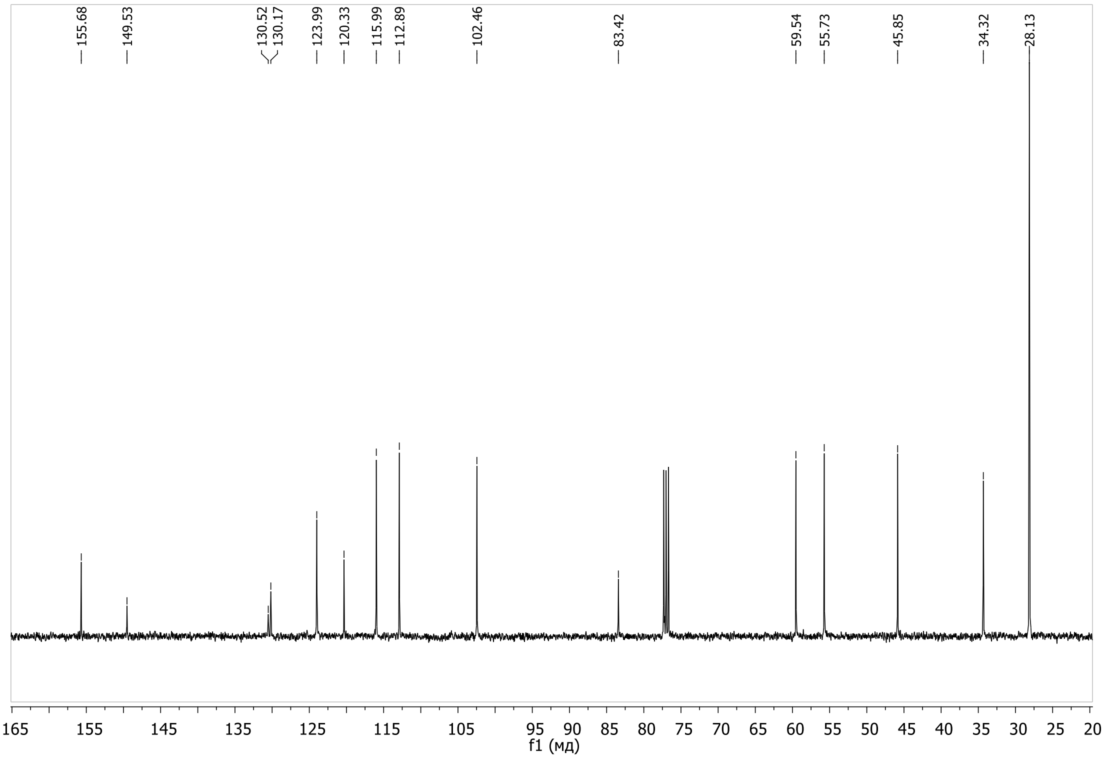

155.68
149.53
130.52
130.17
123.99
120.33
115.99
112.89
102.46
83.42
59.54
55.73
45.85
34.32
28.13
165 155 145 135 125 115 105 95 90 85 80 75 70 65 60 55 50 45 40 35 30 25 20
f1 (мд)

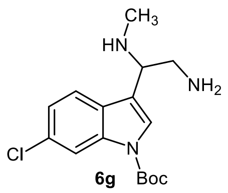
CH3
HN
NH2
Cl
N
6g
Boc

CDCl$_3$

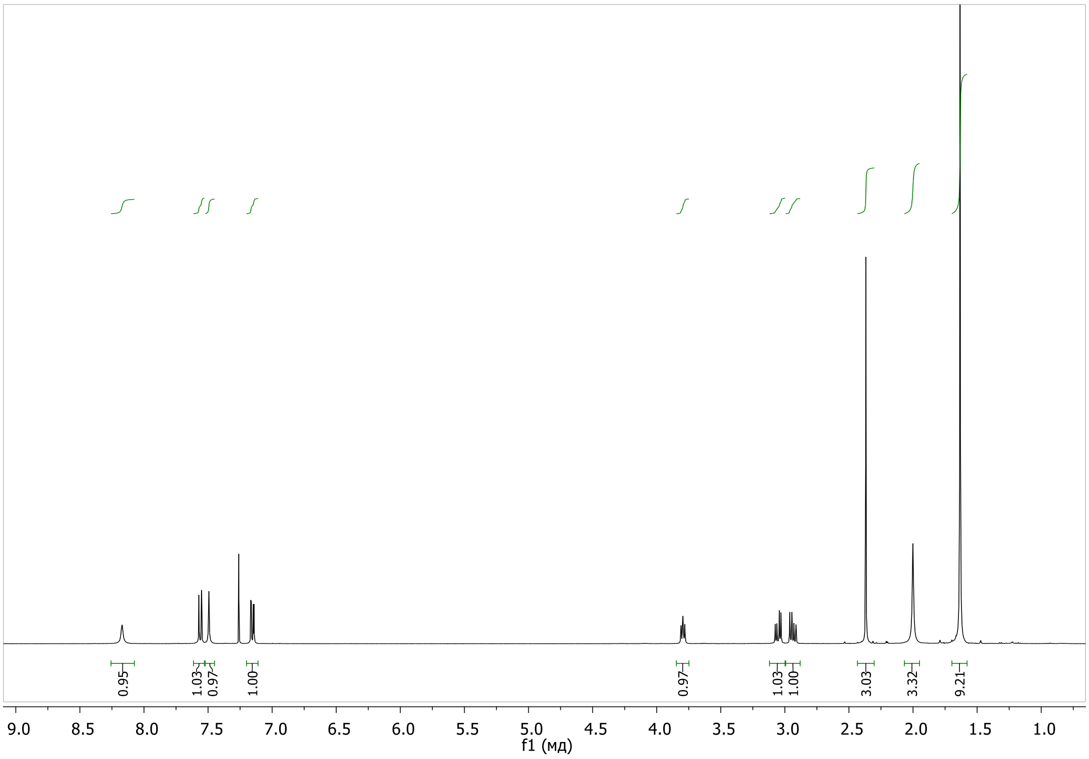
0.95
1.03
0.97
1.00
0.97
1.03
1.00
3.03
3.32
9.21
9.0 8.5 8.0 7.5 7.0 6.5 6.0 5.5 5.0 4.5 4.0 3.5 3.0 2.5 2.0 1.5 1.0
f1 (мд)

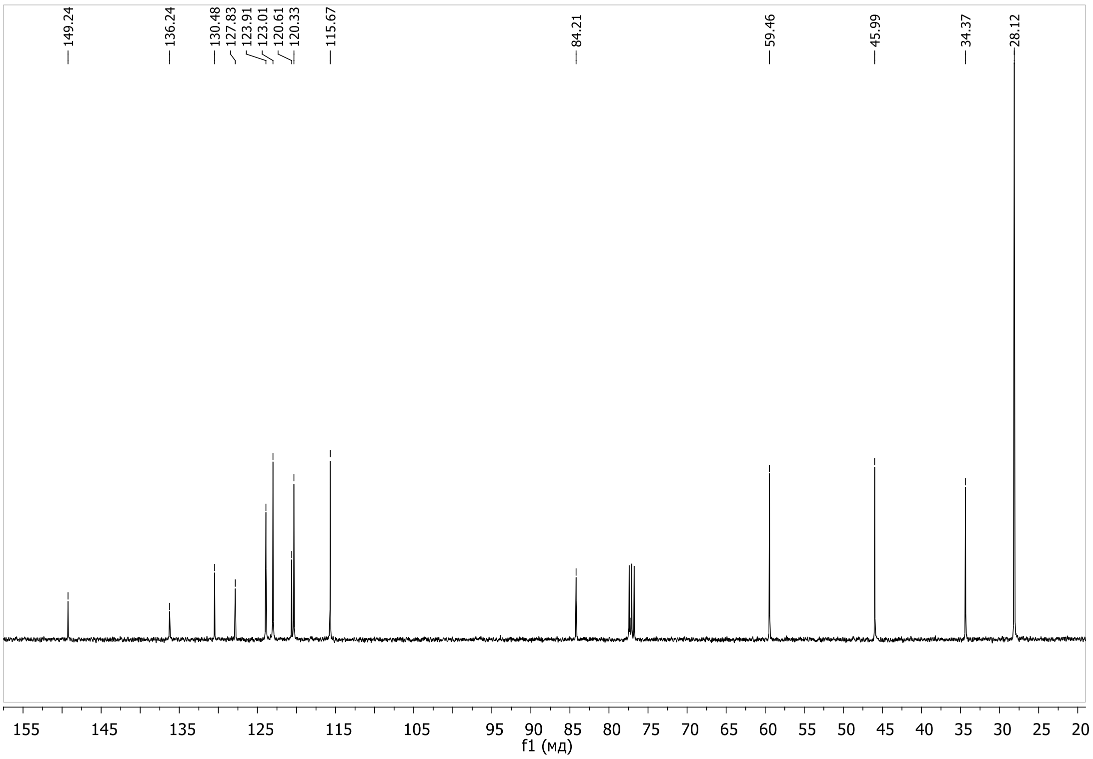
149.24
136.24
130.48
127.83
123.91
123.01
120.61
120.33
115.67
84.21
59.46
45.99
34.37
28.12
155 145 135 125 115 105 95 90 85 80 75 70 65 60 55 50 45 40 35 30 25 20
f1 (мд)

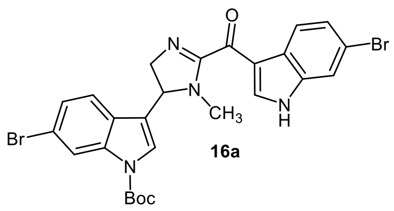
Br
N
O
Br
N
N
CH3
N
H
N
Boc

16a

$CDCl_3$

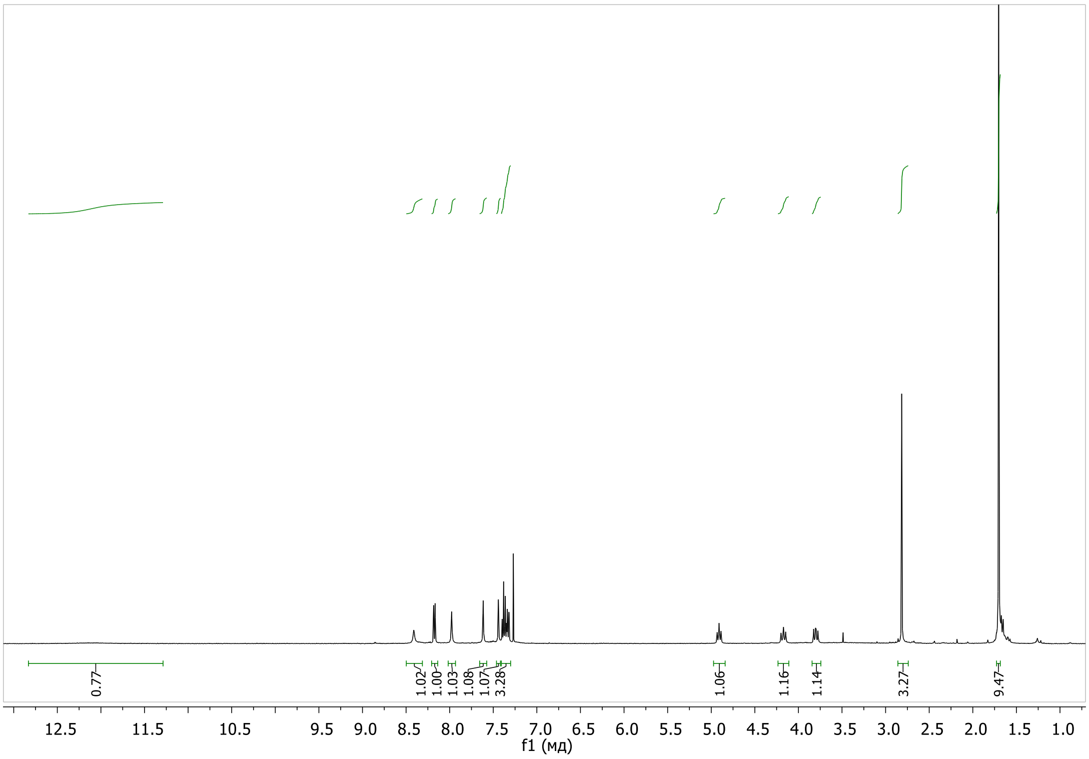
0.77
1.02
1.00
1.03
1.08
1.07
3.28
1.06
1.16
1.14
3.27
9.47
12.5
11.5
10.5
9.5
9.0
8.5
8.0
7.5
7.0
6.5
6.0
5.5
5.0
4.5
4.0
3.5
3.0
2.5
2.0
1.5
1.0
f1 (мд)

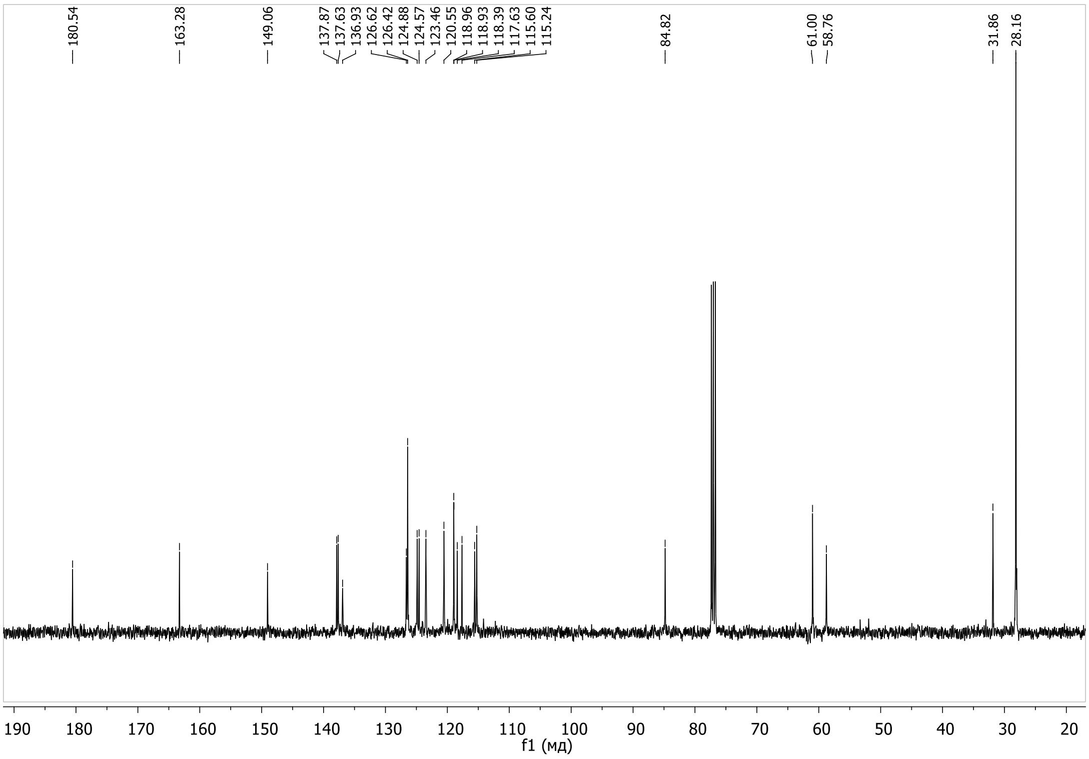
180.54
163.28
149.06
137.87
137.63
136.93
126.62
126.42
124.88
124.57
123.46
120.55
118.96
118.93
118.39
117.63
115.60
115.24
84.82
61.00
58.76
31.86
28.16
190
180
170
160
150
140
130
120
110
100
90
80
70
60
50
40
30
20
f1 (мд)

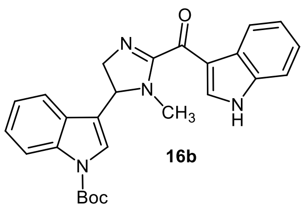
O
N
N
CH3
N
H
N
Boc

16b

CDCl$_3$

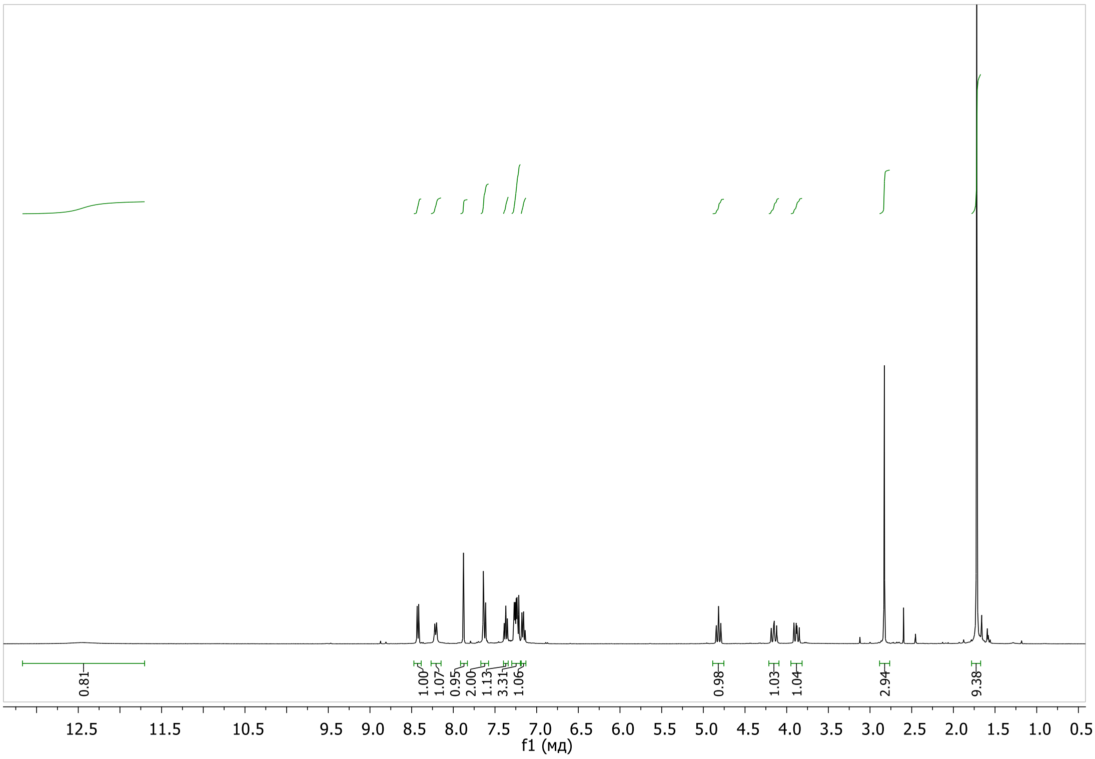
0.81
1.00
1.07
0.95
2.00
1.13
3.31
1.06
0.98
1.03
1.04
2.94
9.38
12.5 11.5 10.5 9.5 9.0 8.5 8.0 7.5 7.0 6.5 6.0 5.5 5.0 4.5 4.0 3.5 3.0 2.5 2.0 1.5 1.0 0.5
f1 (мд)

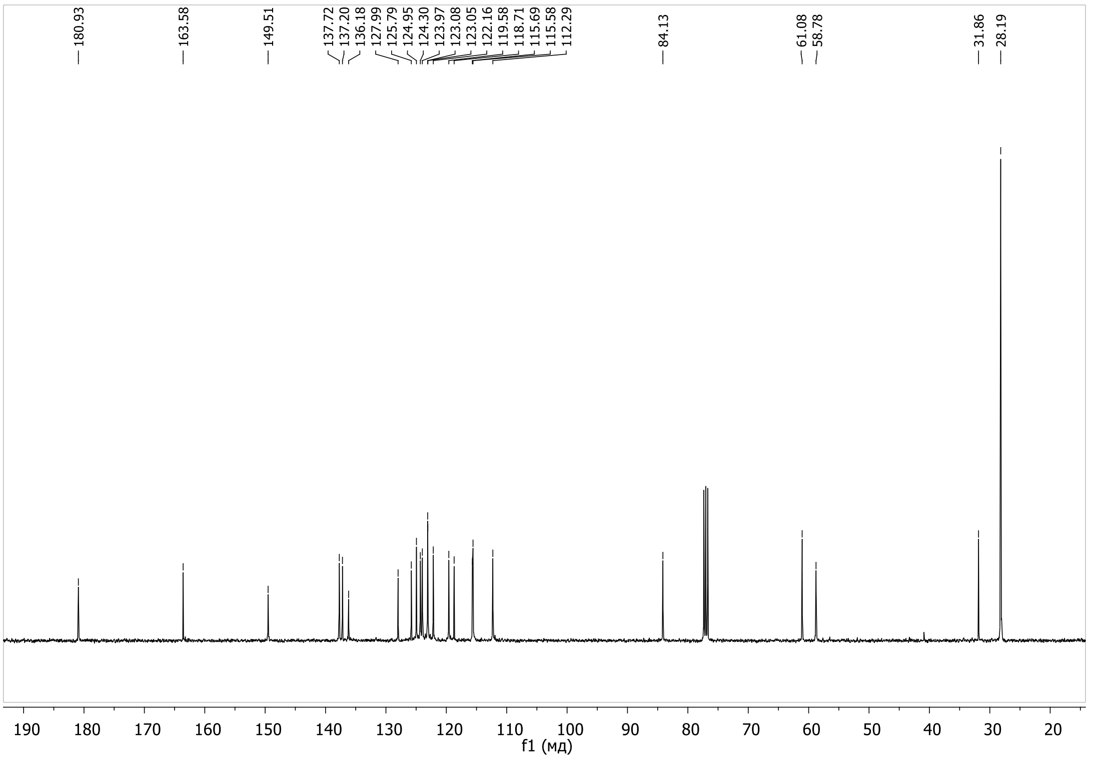
180.93
163.58
149.51
137.72
137.20
136.18
127.99
125.79
124.95
124.30
123.97
123.08
123.05
122.16
119.58
118.71
115.69
115.58
112.29
84.13
61.08
58.78
31.86
28.19
190 180 170 160 150 140 130 120 110 100 90 80 70 60 50 40 30 20
f1 (мд)

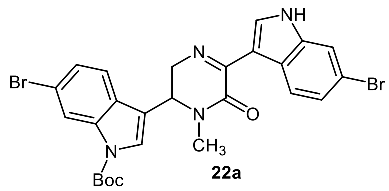
Br
NH
N
Br
N
O
N
CH3
Boc

22a

CDCl3

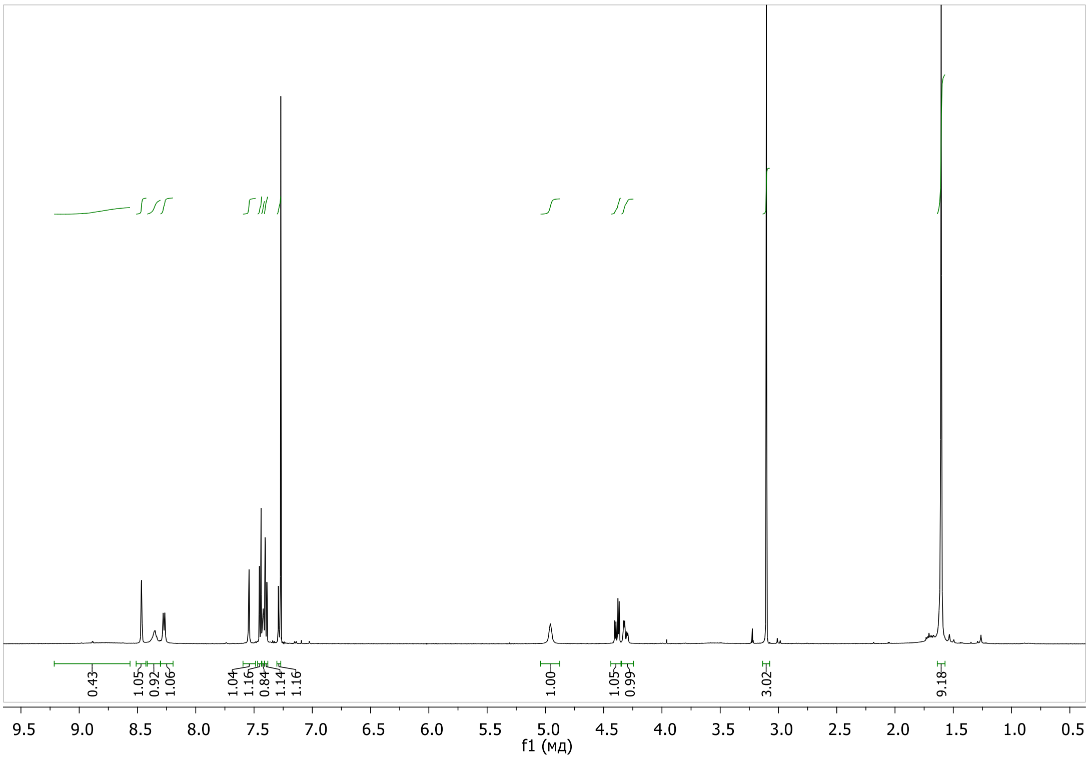
0.43
1.05
0.92
1.06
1.04
1.16
0.84
1.14
1.16
1.00
1.05
0.99
3.02
9.18
9.5 9.0 8.5 8.0 7.5 7.0 6.5 6.0 5.5 5.0 4.5 4.0 3.5 3.0 2.5 2.0 1.5 1.0 0.5
f1 (мд)

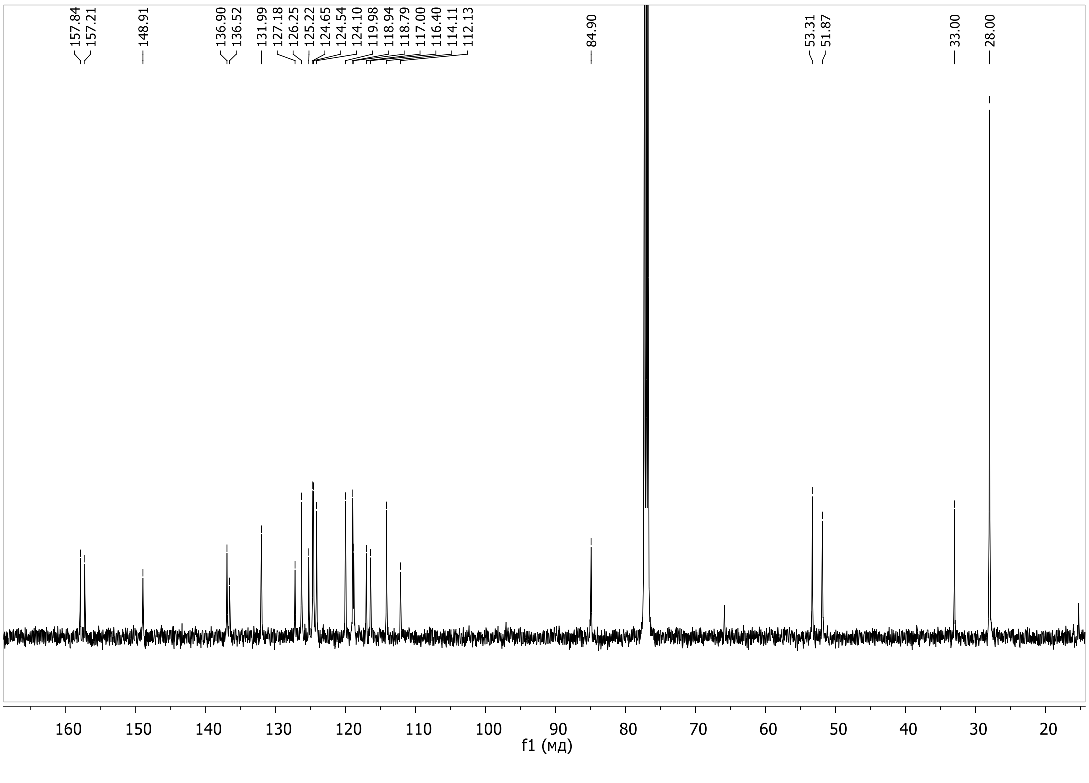
157.84
157.21
148.91
136.90
136.52
131.99
127.18
126.25
125.22
124.65
124.54
124.10
119.98
118.94
118.79
117.00
116.40
114.11
112.13
84.90
53.31
51.87
33.00
28.00
160 150 140 130 120 110 100 90 80 70 60 50 40 30 20
f1 (мд)

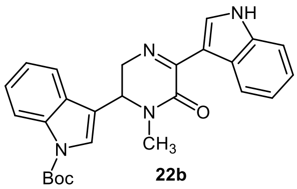
NH
N
O
N
CH3
N
Boc

22b

CDCl$_3$

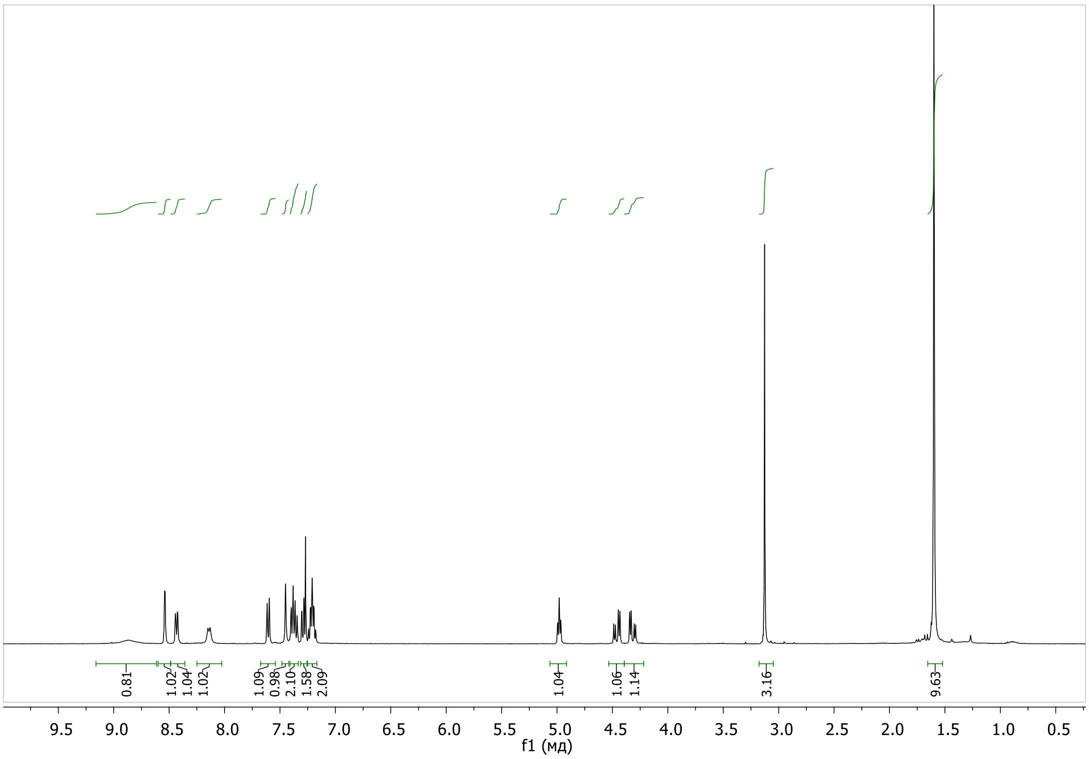
0.81
1.02
1.04
1.02
1.09
0.98
2.10
1.58
2.09
1.04
1.06
1.14
3.16
9.63
9.5 9.0 8.5 8.0 7.5 7.0 6.5 6.0 5.5 5.0 4.5 4.0 3.5 3.0 2.5 2.0 1.5 1.0 0.5
f1 (мд)

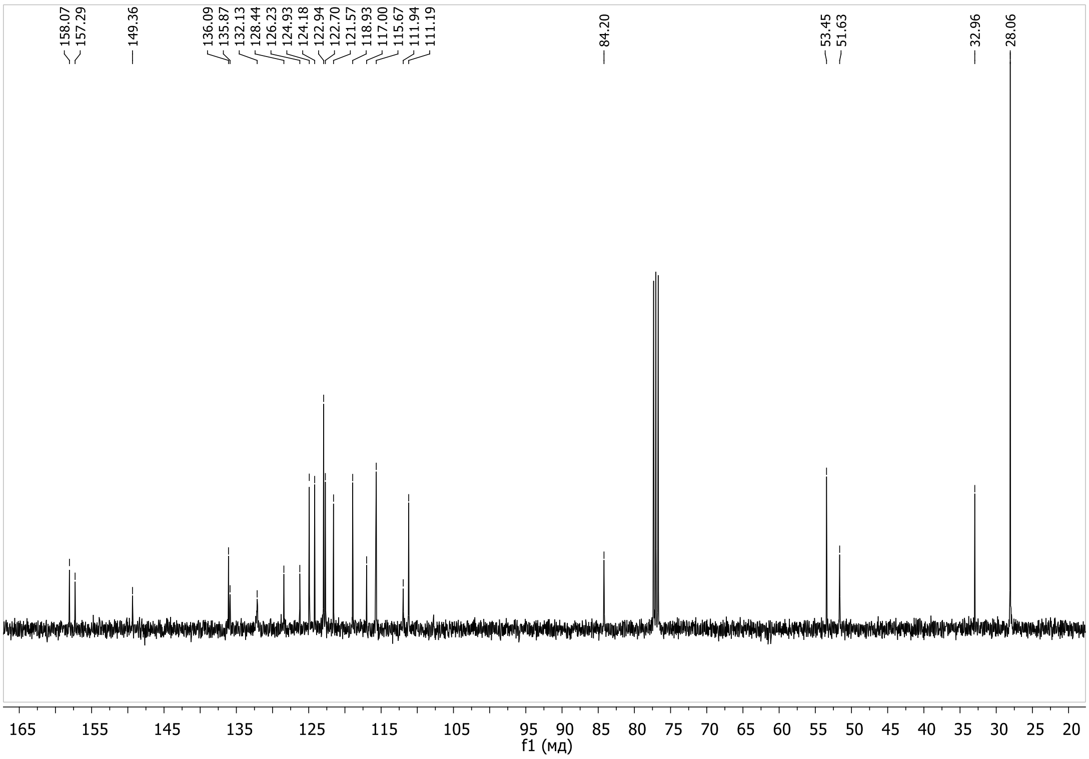
158.07
157.29
149.36
136.09
135.87
132.13
128.44
126.23
124.93
124.18
122.94
122.70
121.57
118.93
117.00
115.67
111.94
111.19
84.20
53.45
51.63
32.96
28.06
165 155 145 135 125 115 105 95 90 85 80 75 70 65 60 55 50 45 40 35 30 25 20
f1 (мд)

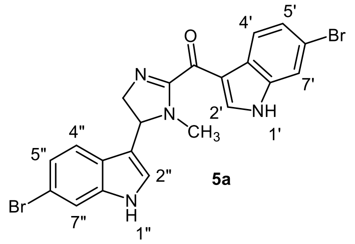
O
4'
5'
Br
7'
N
2'
NH
1'
N
CH3
4"
5"
2"
Br
7"
N
H
1"

5a

DMSO-d$_6$

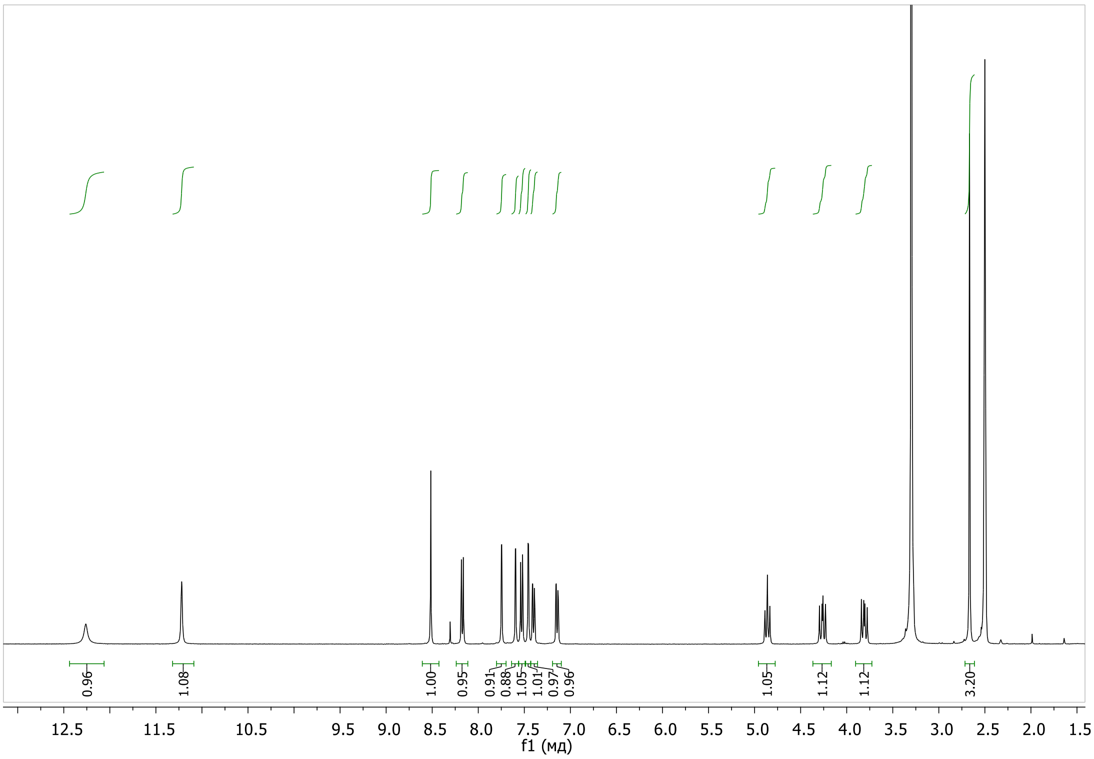
0.96
1.08
1.00
0.95
0.91
0.88
1.05
1.01
0.97
0.96
1.05
1.12
1.12
3.20
12.5 11.5 10.5 9.5 9.0 8.5 8.0 7.5 7.0 6.5 6.0 5.5 5.0 4.5 4.0 3.5 3.0 2.5 2.0 1.5
f1 (мд)

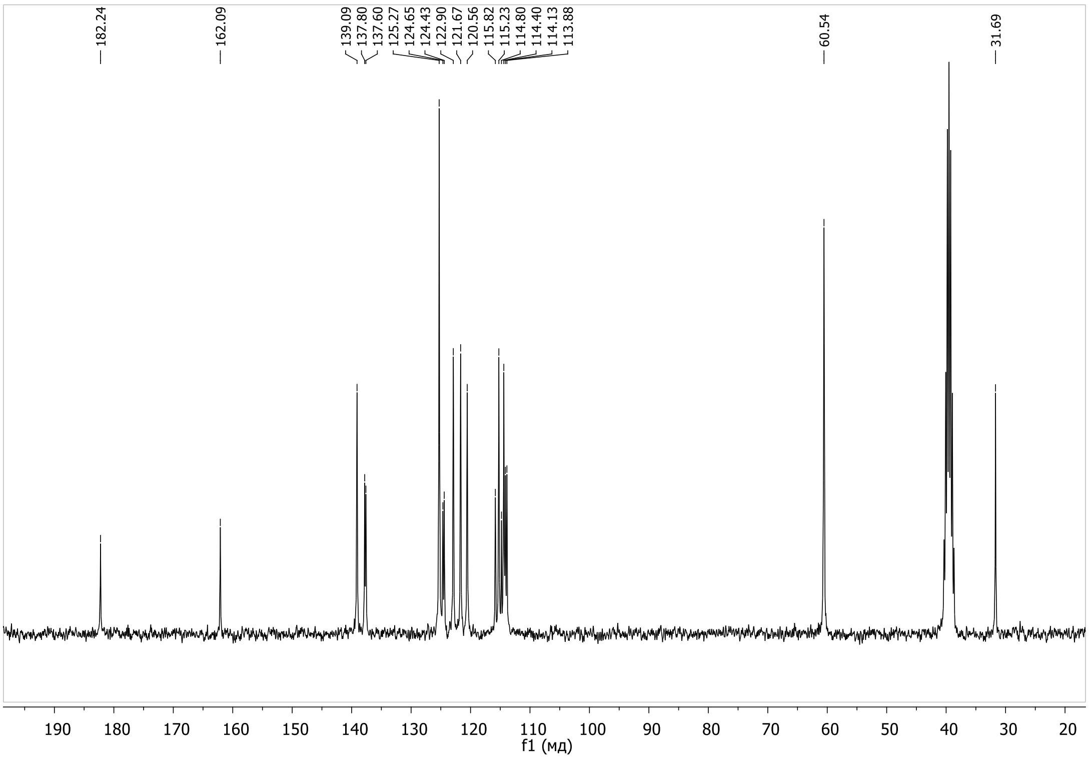
182.24
162.09
139.09
137.80
137.60
125.27
124.65
124.43
122.90
121.67
120.56
115.82
115.23
114.80
114.40
114.13
113.88
60.54
31.69
190 180 170 160 150 140 130 120 110 100 90 80 70 60 50 40 30 20
f1 (мд)

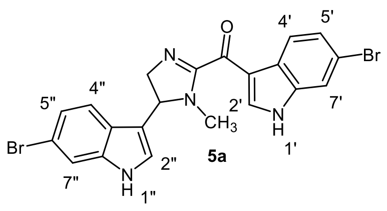
5a
Br
N
O
H
CH3
4'
5'
7'
2'
1'
4"
5"
7"
2"
1"

acetone-d_6

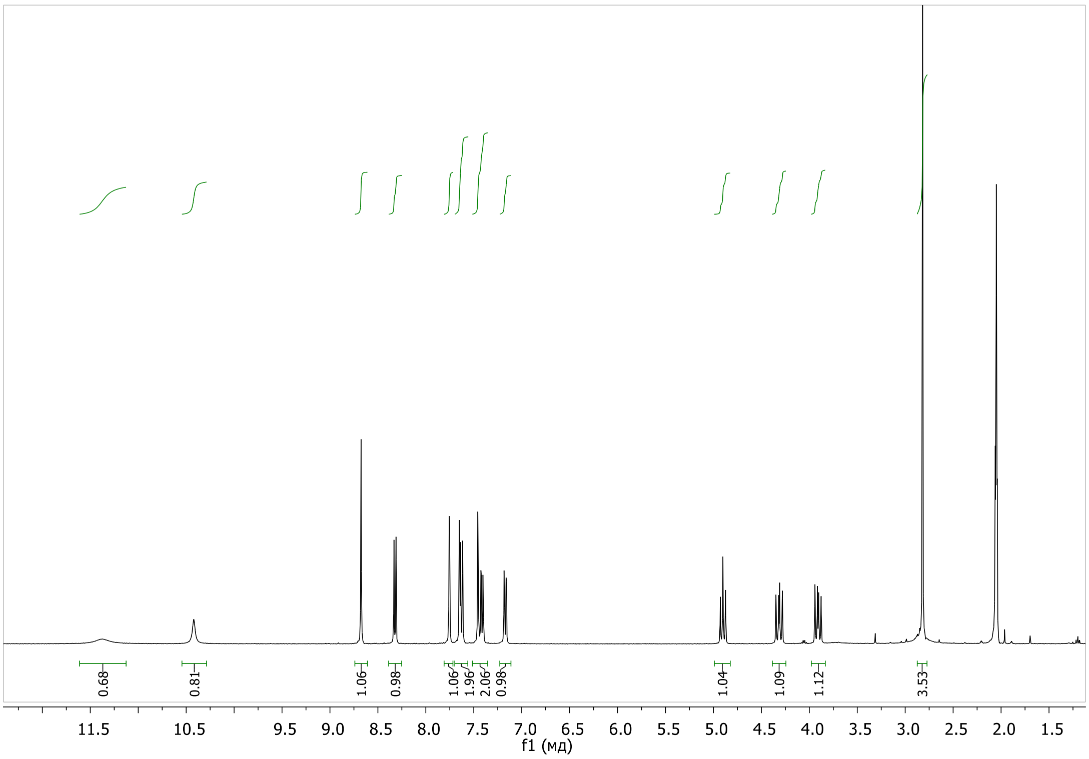
0.68
0.81
1.06
0.98
1.06
1.96
2.06
0.98
1.04
1.09
1.12
3.53
11.5
10.5
9.5
9.0
8.5
8.0
7.5
7.0
6.5
6.0
5.5
5.0
4.5
4.0
3.5
3.0
2.5
2.0
1.5
f1 (мд)

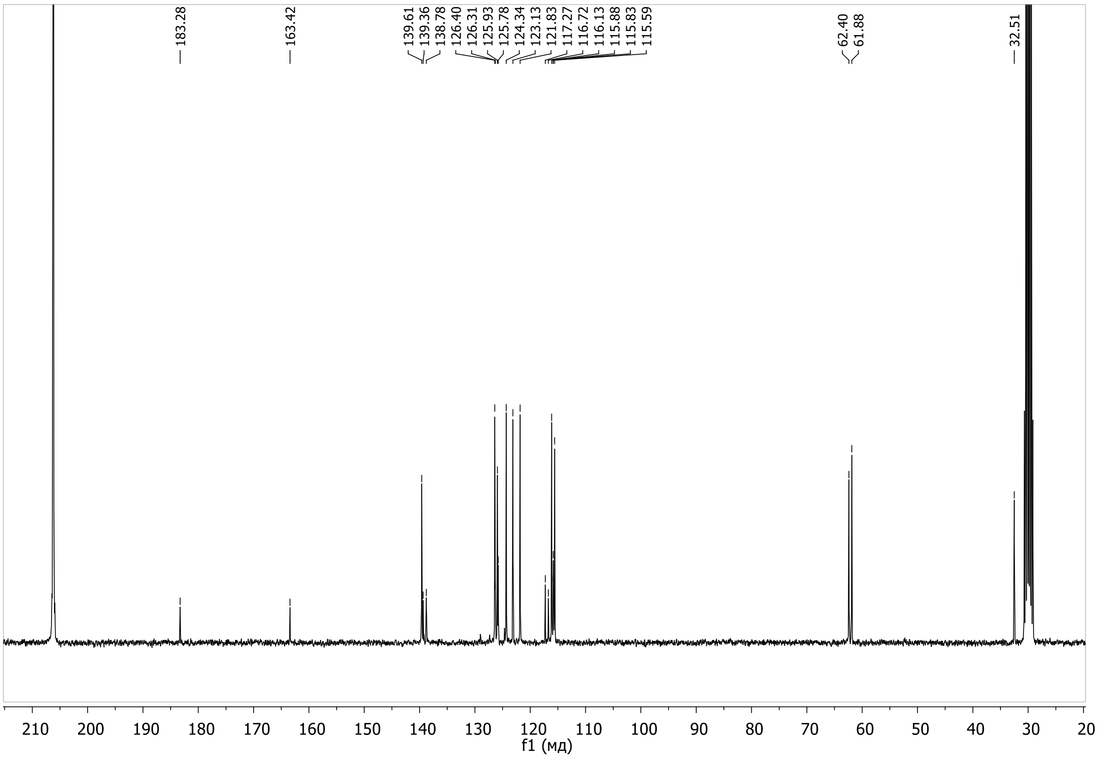
183.28
163.42
139.61
139.36
138.78
126.40
126.31
125.93
125.78
124.34
123.13
121.83
117.27
116.72
116.13
115.88
115.83
115.59
62.40
61.88
32.51
210
200
190
180
170
160
150
140
130
120
110
100
90
80
70
60
50
40
30
20
f1 (мд)

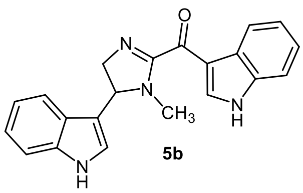

O
N
N
CH3
N
H
N
H

5b

DMSO-d6

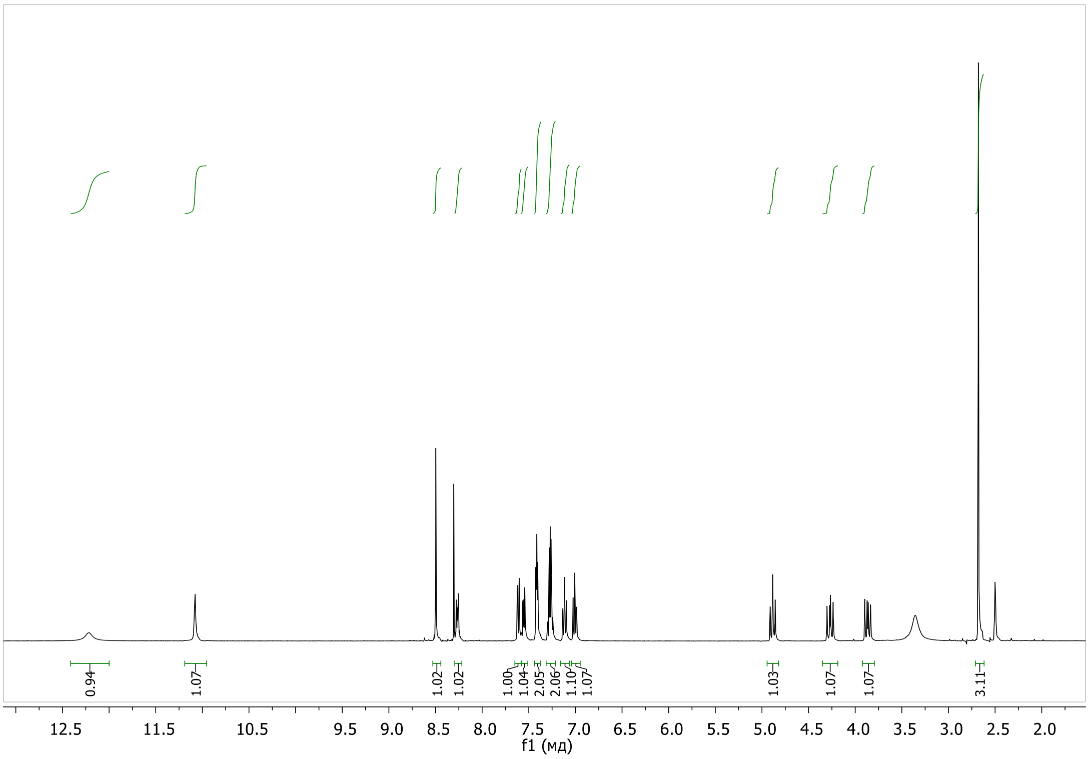

0.94
1.07
1.02
1.02
1.00
1.04
2.05
2.06
1.10
1.07
1.03
1.07
1.07
3.11
12.5 11.5 10.5 9.5 9.0 8.5 8.0 7.5 7.0 6.5 6.0 5.5 5.0 4.5 4.0 3.5 3.0 2.5 2.0
f1 (мд)

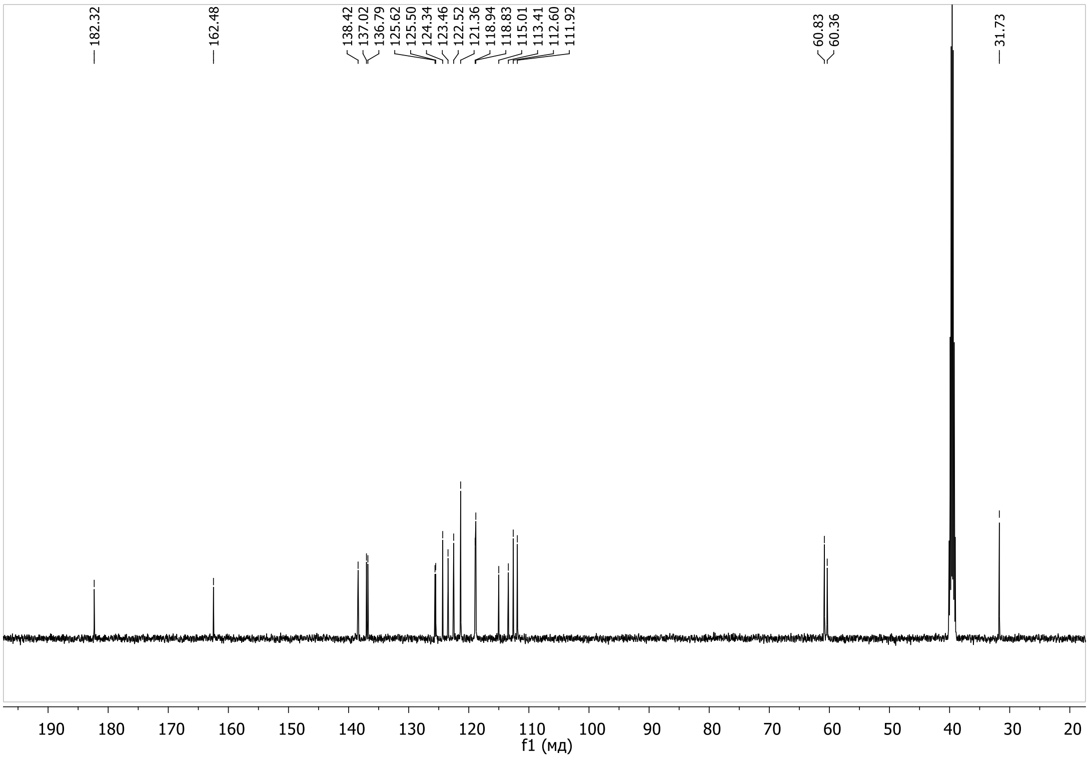

182.32
162.48
138.42
137.02
136.79
125.62
125.50
124.34
123.46
122.52
121.36
118.94
118.83
115.01
113.41
112.60
111.92
60.83
60.36
31.73
190 180 170 160 150 140 130 120 110 100 90 80 70 60 50 40 30 20
f1 (мд)

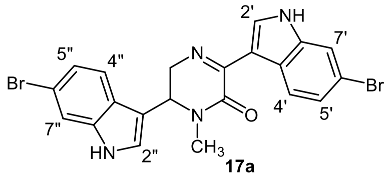

2'
NH
7'
5"
4"
N
Br
Br
4'
O
5'
7"
N
HN
2"
CH3

17a

DMSO-d_6

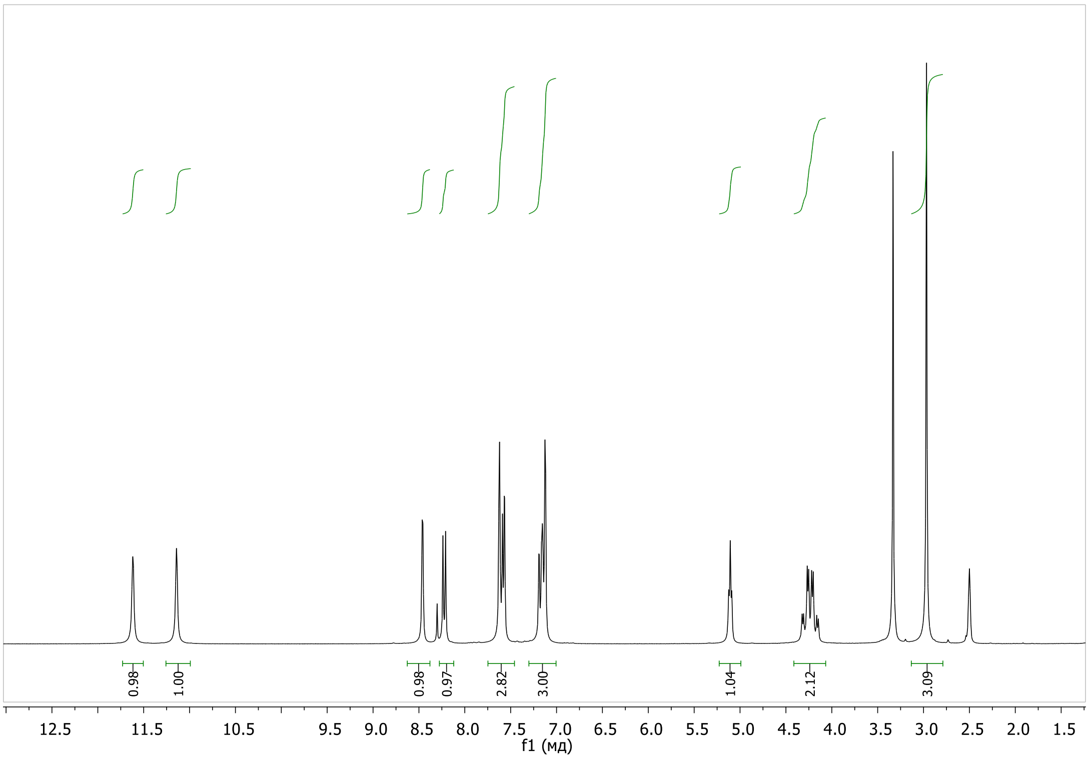

0.98
1.00
0.98
0.97
2.82
3.00
1.04
2.12
3.09
12.5
11.5
10.5
9.5
9.0
8.5
8.0
7.5
7.0
6.5
6.0
5.5
5.0
4.5
4.0
3.5
3.0
2.5
2.0
1.5
f1 (мд)

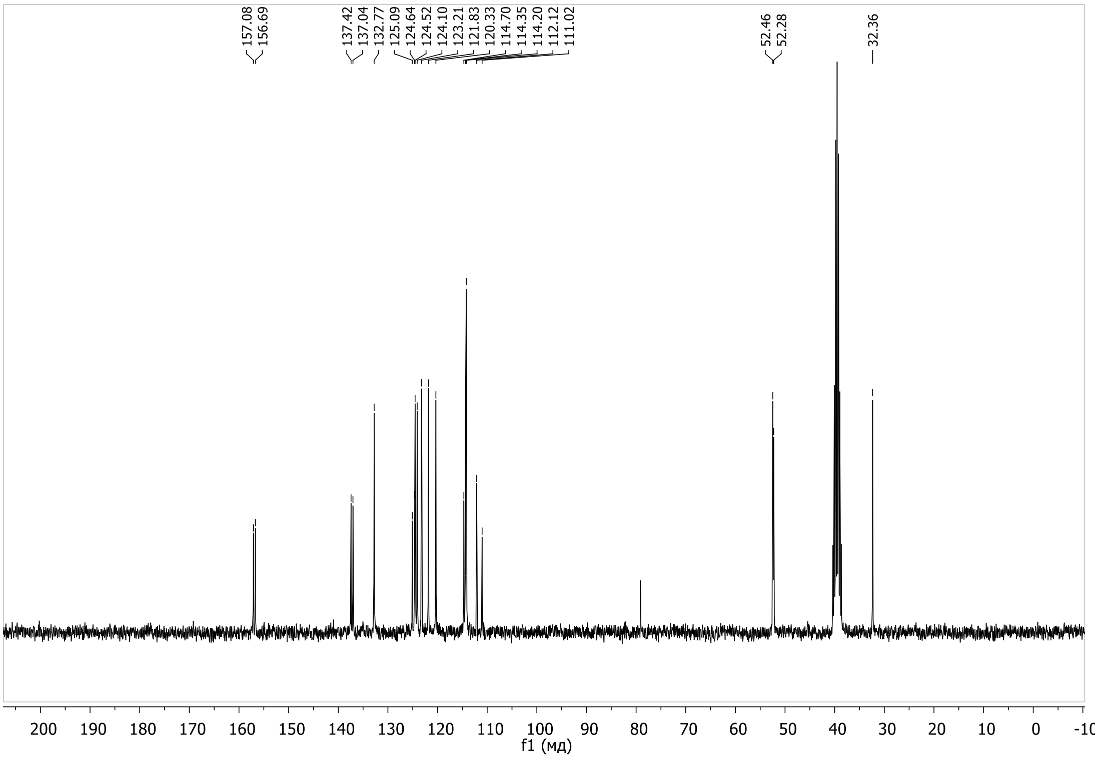

157.08
156.69
137.42
137.04
132.77
125.09
124.64
124.52
124.10
123.21
121.83
120.33
114.70
114.35
114.20
112.12
111.02
52.46
52.28
32.36
200
190
180
170
160
150
140
130
120
110
100
90
80
70
60
50
40
30
20
10
0
f1 (мд)

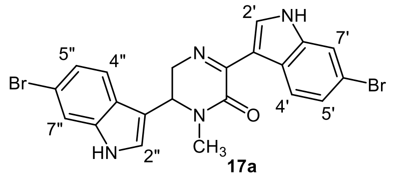

17a

acetone-d_6

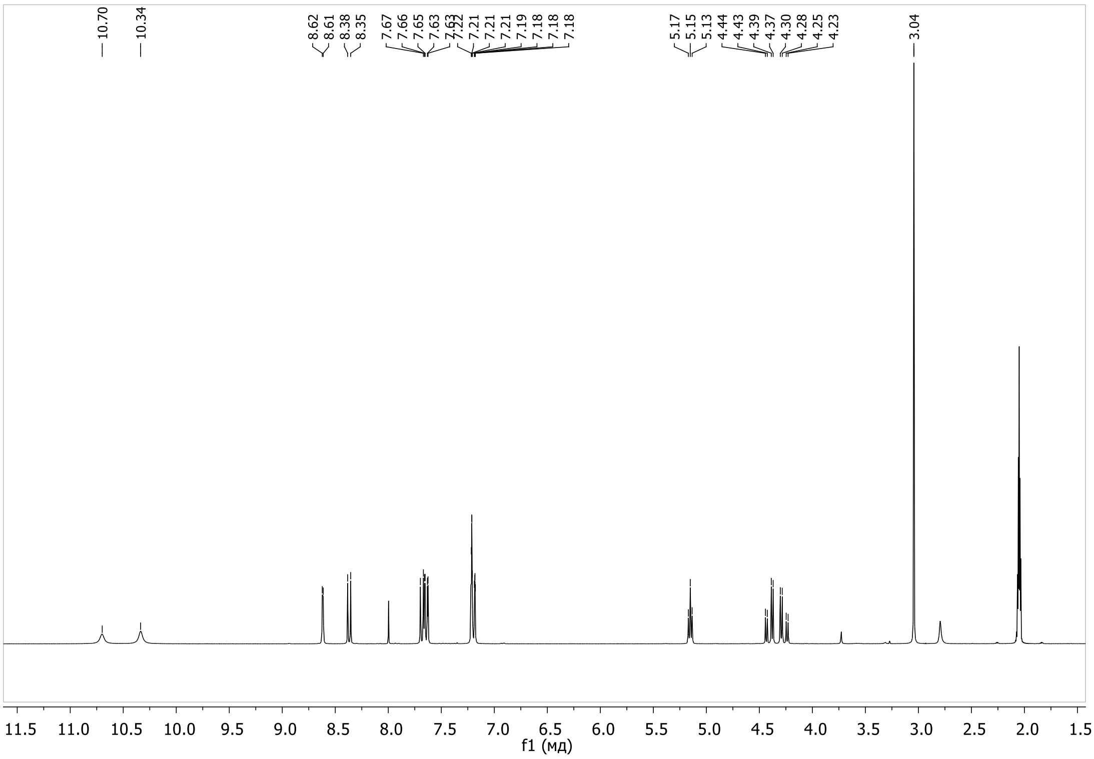

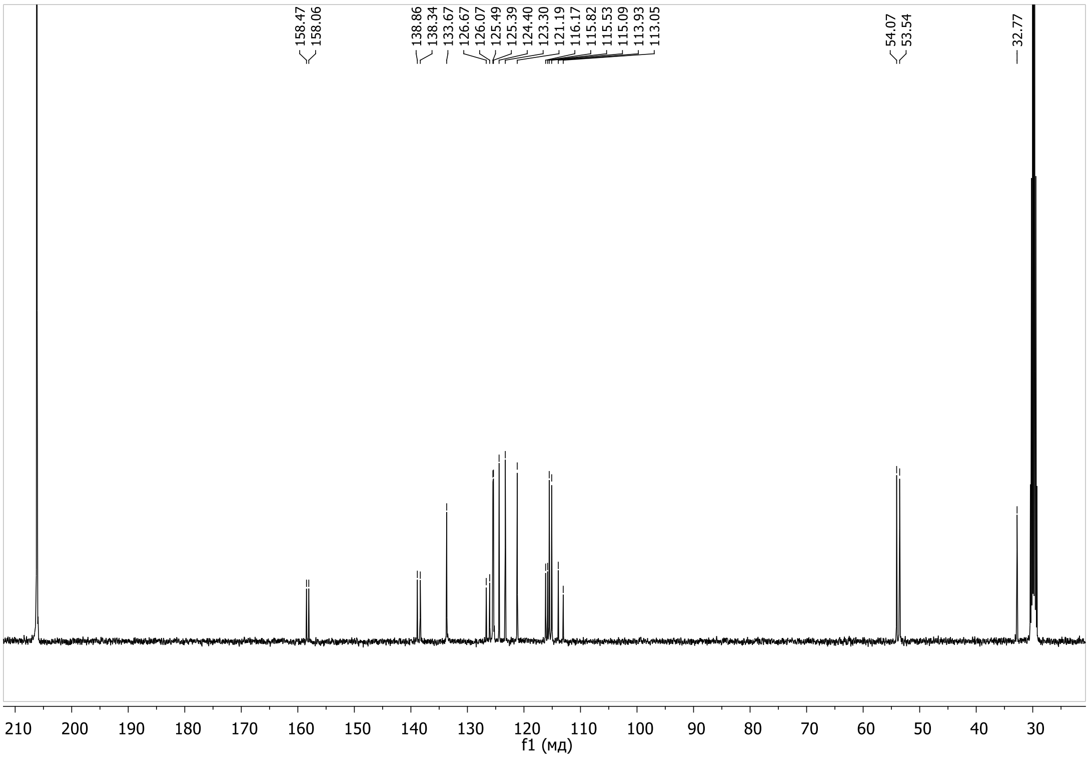

Double resonance and COSY experiments for topsentin C (**17a**)

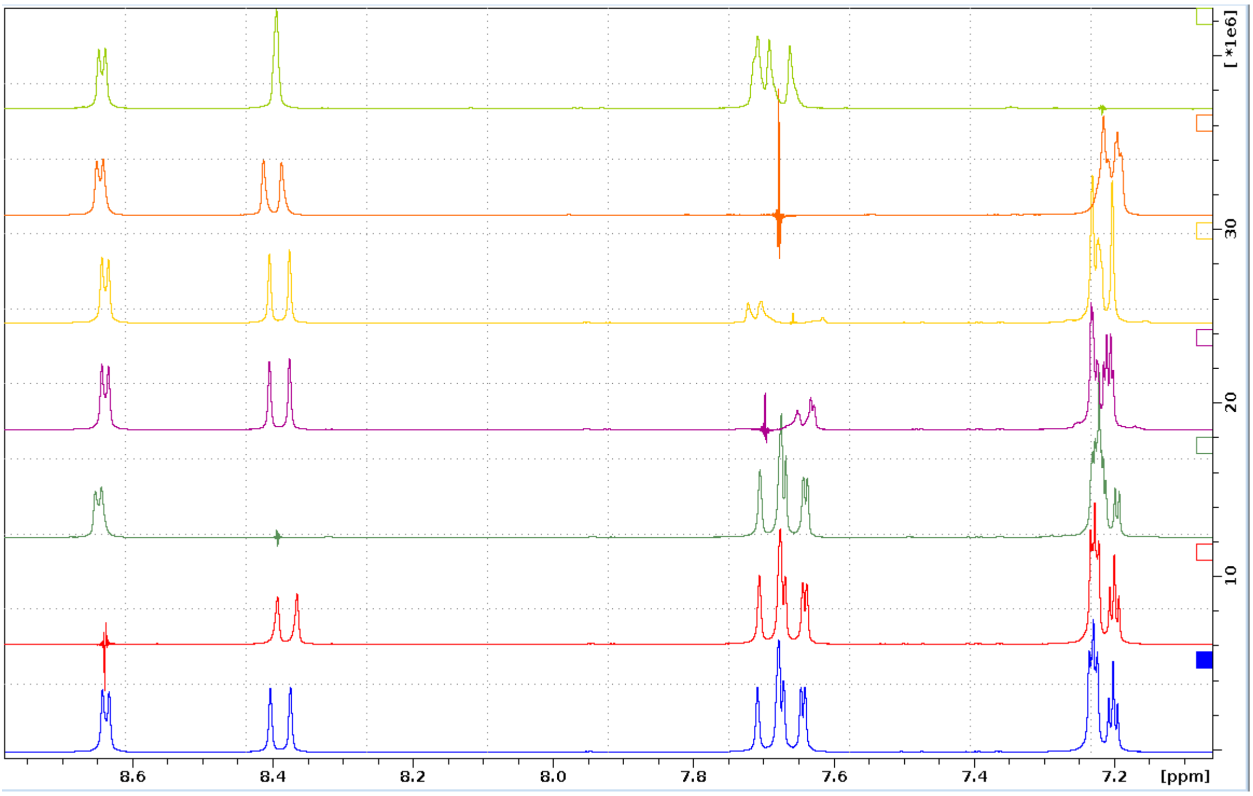

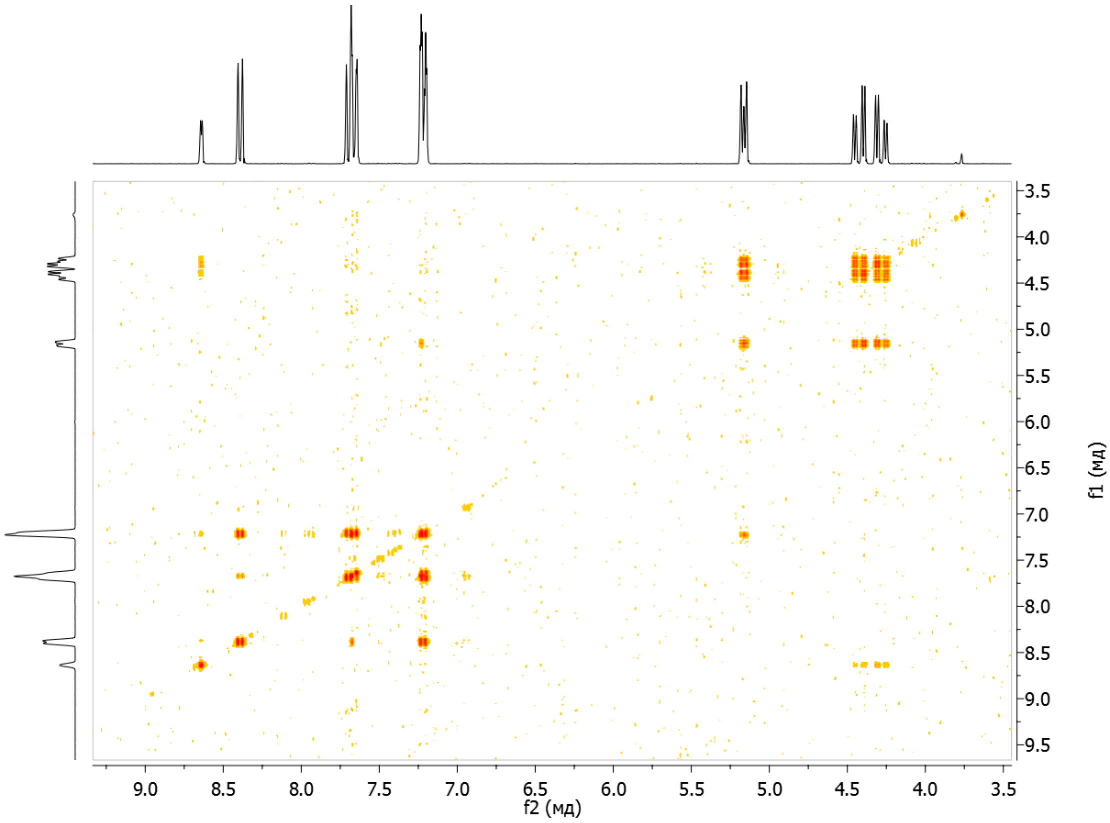

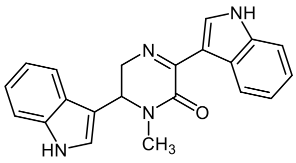
NH
N
N
O
HN
CH3

17b

DMSO-d[6]

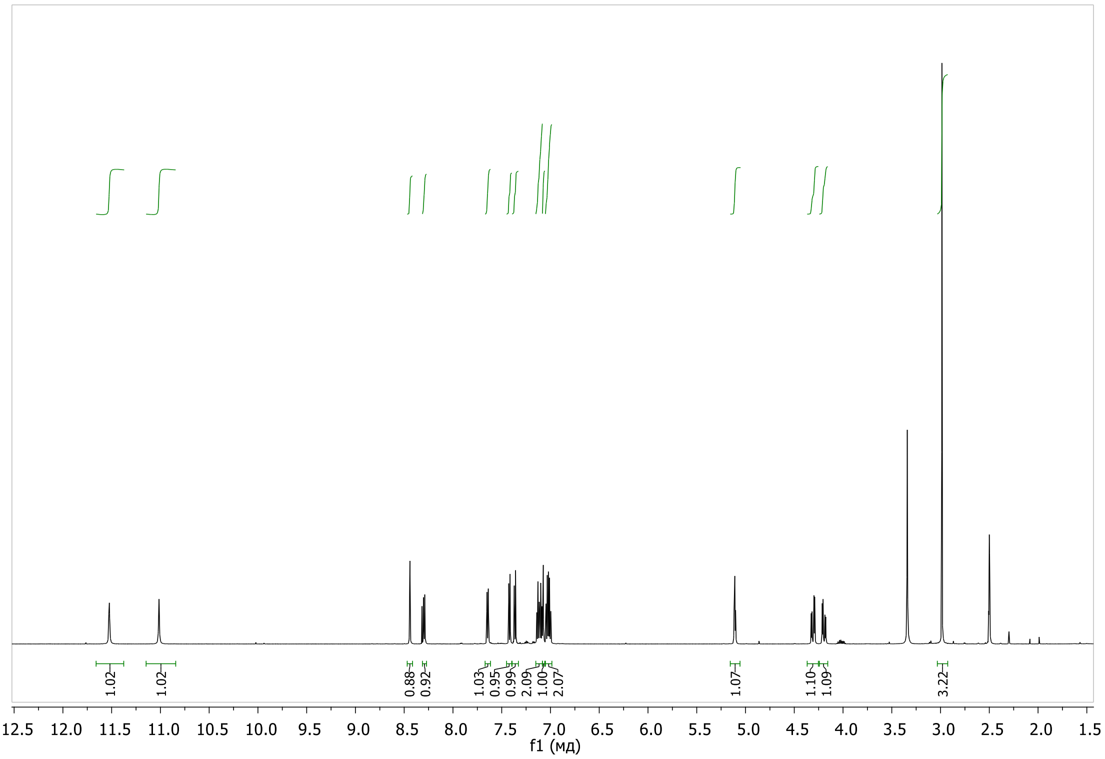
1.02
1.02
0.88
0.92
1.03
0.95
0.99
2.09
1.00
2.07
1.07
1.10
1.09
3.22
12.5 12.0 11.5 11.0 10.5 10.0 9.5 9.0 8.5 8.0 7.5 7.0 6.5 6.0 5.5 5.0 4.5 4.0 3.5 3.0 2.5 2.0 1.5
f1 (мд)

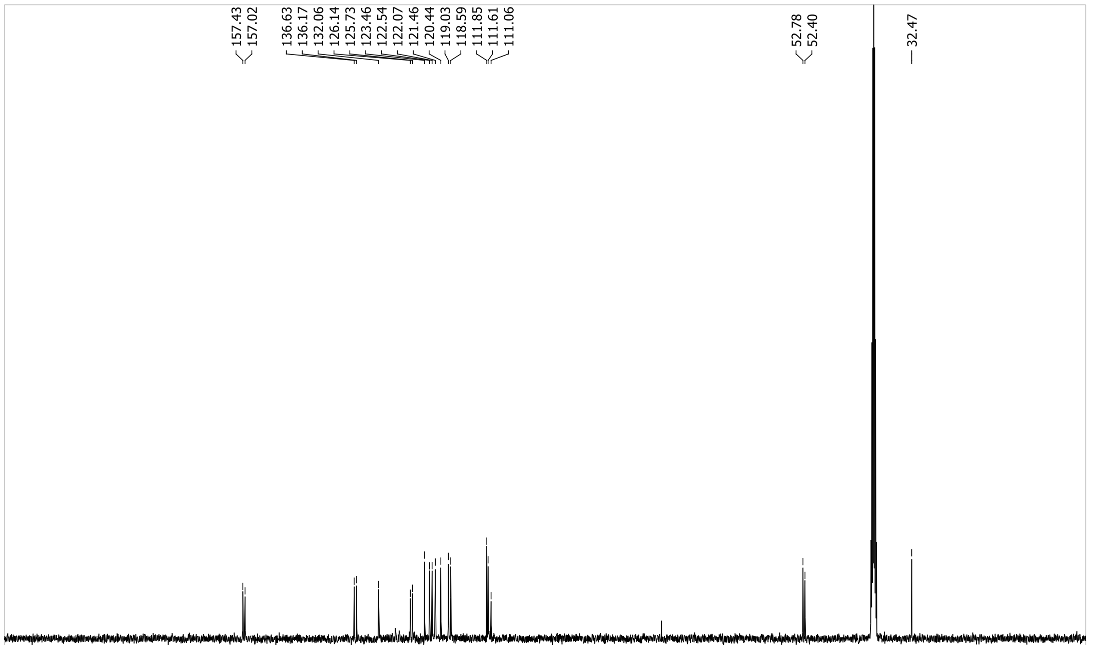
157.43
157.02
136.63
136.17
132.06
126.14
125.73
123.46
122.54
122.07
121.46
120.44
119.03
118.59
111.85
111.61
111.06
52.78
52.40
32.47
200 190 180 170 160 150 140 130 120 110 100 90 80 70 60 50 40 30 20 10 0
f1 (мд)

3. X-Ray Data for Topsentin C (**17a**)

Single crystals of $C_{23.25}H_{22}Br_2N_4O_{1.75}$ were colorless prisms. A suitable crystal was selected and the measurements were carried out on a Bruker APEX-II CCD diffractometer. The crystal was kept at 120 K during data collection. Using Olex2 [1], the structure was solved with the ShelXT [2] structure solution program using Intrinsic Phasing and refined with the ShelXL [3] refinement package using Least Squares minimisation. Molecular structure of **17a** is presented at Fig. 1.

1. Dolomanov, O.V., Bourhis, L.J., Gildea, R.J, Howard, J.A.K. & Puschmann, H. (2009), J. Appl. Cryst. 42, 339-341.
2. Sheldrick, G.M. (2015). Acta Cryst. A71, 3-8.
3. Sheldrick, G.M. (2015). Acta Cryst. C71, 3-8.

Table 1 Crystal data and structure refinement for **17a**.

Identification code	tops5
Empirical formula	$C_{23.25}H_{22}Br_2N_4O_{1.75}$
Formula weight	545.27
Temperature/K	120
Crystal system	triclinic
Space group	P-1
a/Å	11.835(3)
b/Å	13.374(3)
c/Å	15.627(4)
α/°	71.158(4)
β/°	79.735(5)
γ/°	82.497(4)
Volume/Å^3	2296.5(10)
Z	4
ρ_{calc}g/cm^3	1.577
μ/mm^{-1}	3.556
F(000)	1094.0
Crystal size/mm^3	0.1 × 0.07 × 0.07
Radiation	MoKα (λ = 0.71073)
2Θ range for data collection/°	3.556 to 52.042
Index ranges	$-14 \leq h \leq 14$, $-16 \leq k \leq 16$, $-19 \leq l \leq 19$
Reflections collected	14973
Independent reflections	9024 [R_{int} = 0.0910, R_{sigma} = 0.1864]
Data/restraints/parameters	9024/5/557
Goodness-of-fit on F^2	0.981
Final R indexes [I>=2σ (I)]	R_1 = 0.0800, wR_2 = 0.1667
Final R indexes [all data]	R_1 = 0.2038, wR_2 = 0.2183
Largest diff. peak/hole / e Å^{-3}	1.18/-0.61

Table 2 Fractional Atomic Coordinates (×10^4) and Equivalent Isotropic Displacement Parameters (Å^2×10^3) for **17a**. U_{eq} is defined as 1/3 of of the trace of the orthogonalised U_{IJ}

tensor.

Atom	*x*	*y*	*z*	U(eq)
Br1'	-1609.6(10)	7736.3(8)	2862.7(8)	48.2(3)
Br1	7961.4(10)	5065.3(9)	8065.5(9)	61.1(4)
Br2	-2224.0(12)	7792.5(10)	9610.1(8)	66.7(4)
Br2'	562.3(14)	8543.3(10)	10513.4(9)	75.3(5)
O1'	4003(6)	4466(5)	5996(5)	46.7(19)
O1	3931(5)	9493(5)	3925(5)	42.3(17)
N2	3834(6)	9315(5)	6259(5)	27.5(18)
N4	610(6)	7601(6)	6614(5)	35(2)
N2'	1180(7)	5299(5)	6828(5)	36(2)
N1	5840(6)	6718(6)	5163(6)	42(2)
N3'	3285(7)	4411(6)	7436(6)	42(2)
N3	2490(7)	10140(6)	4820(5)	36(2)
N4'	3099(7)	7457(6)	7741(5)	37(2)
C16	469(7)	9071(6)	7033(6)	30(2)
N1'	2601(8)	6706(6)	3842(6)	48(2)
C17	76(7)	8043(7)	7262(6)	28(2)
C9	4082(8)	9041(6)	5519(6)	30(2)
C14'	2558(7)	5820(6)	8121(6)	28(2)
C17'	2356(9)	7319(7)	8540(6)	35(2)
C16'	1997(7)	6275(6)	8808(6)	27(2)
C10'	3221(9)	4672(7)	6556(8)	38(3)
C7	4924(7)	8160(6)	5494(7)	33(2)
C6	5638(8)	7610(7)	6184(7)	35.0(17)
C18	-720(8)	7645(6)	8036(6)	34(2)
C14	1264(7)	9197(6)	6212(6)	29(2)
C1	6158(8)	6705(7)	5968(7)	36(2)
C2	6877(8)	5955(7)	6500(7)	35.0(17)
C15'	3236(7)	6541(6)	7497(6)	30(2)
C21'	1249(8)	5926(7)	9601(6)	33(2)
C3'	-486(9)	7142(7)	3669(7)	43(3)
C9'	2083(8)	5266(7)	6266(7)	37(2)
C11'	1244(8)	4734(7)	7786(7)	37(3)
C5'	-161(9)	6197(7)	5187(7)	38(3)
C15	1338(7)	8292(7)	5991(7)	34(2)
C6'	1056(8)	6241(7)	4887(7)	36(2)
C5	5871(8)	7737(7)	6988(7)	35(2)
C7'	2059(8)	5817(7)	5313(7)	33(2)
C21	8(8)	9696(7)	7585(7)	40(3)
C20'	830(9)	6610(7)	10095(7)	42(3)
C18'	1945(9)	8000(7)	9036(7)	41(3)

C11	2930(8)	10169(7)	6256(7)	35(2)
C12	1978(8)	10133(7)	5743(7)	37(2)
C20	-800(9)	9316(7)	8342(7)	43(3)
C4'	-894(10)	6651(7)	4575(7)	45(3)
C10	3495(8)	9580(7)	4683(7)	35(2)
C8'	2976(9)	6152(7)	4629(7)	41(3)
C3	7040(8)	6111(7)	7263(7)	42(3)
C2'	662(9)	7233(8)	3343(8)	46(3)
C12'	2385(8)	4741(7)	8070(7)	37(2)
C8	5119(8)	7593(7)	4879(7)	37(2)
C1'	1401(10)	6766(7)	3966(8)	45(3)
C19	-1137(9)	8289(8)	8545(6)	41(3)
C4	6574(8)	6996(7)	7521(7)	42(3)
C13	1799(9)	10576(9)	4084(8)	56(3)
C19'	1184(10)	7635(8)	9809(7)	47(3)
C13'	4381(10)	3921(8)	7731(8)	61(3)
O2'	6095(9)	1549(7)	7332(6)	92(3)
C23'	5836(19)	724(16)	8157(11)	159(9)
C22'	4650(20)	340(30)	8272(16)	232(16)
C24'	6160(20)	1200(30)	8854(17)	253(17)
O2	3780(17)	4370(17)	9931(19)	121(6)
C23	4960(30)	4560(30)	9710(30)	121(6)
C22	5010(30)	5640(30)	9750(30)	121(6)
C24	5740(20)	3530(20)	9880(30)	121(6)

Table 3 Anisotropic Displacement Parameters (Å^2×10^3) for **17a**. The Anisotropic displacement factor exponent takes the form: $-2\pi^2[h^2a^{*2}U_{11}+2hka^*b^*U_{12}+\ldots]$.

**Atom**	**U_{11}**	**U_{22}**	**U_{33}**	**U_{23}**	**U_{13}**	**U_{12}**
Br1'	60.5(8)	30.4(5)	59.0(7)	-21.3(5)	-8.1(6)	-3.2(5)
Br1	53.4(8)	39.0(6)	88.2(10)	-7.7(6)	-32.6(7)	5.8(5)
Br2	78.9(10)	60.6(8)	50.3(8)	-13.7(6)	18.8(7)	-16.8(7)
Br2'	118.6(12)	54.5(8)	53.9(8)	-31.5(6)	2.1(8)	7.8(7)
O1'	35(4)	43(4)	66(5)	-30(4)	8(4)	-4(3)
O1	34(4)	46(4)	47(5)	-12(4)	-9(4)	-4(3)
N2	22(4)	19(4)	44(5)	-13(4)	-2(4)	-10(3)
N4	33(5)	27(4)	49(5)	-19(4)	-3(4)	-2(4)
N2'	46(5)	24(4)	40(5)	-21(4)	8(4)	-7(4)
N1	32(5)	30(5)	58(6)	-16(4)	2(4)	7(4)
N3'	42(6)	30(5)	55(6)	-21(4)	7(5)	-4(4)
N3	31(5)	28(4)	46(5)	-4(4)	-16(4)	4(4)
N4'	49(5)	24(4)	41(5)	-8(4)	-8(4)	-10(4)

C16	28(5)	15(4)	47(6)	-12(4)	-8(5)	2(4)
N1'	48(6)	31(5)	55(6)	-11(4)	11(5)	-5(4)
C17	23(5)	25(5)	32(5)	-5(4)	-2(4)	-1(4)
C9	34(6)	18(4)	33(6)	6(4)	-10(5)	-15(4)
C14'	26(5)	16(4)	38(6)	-6(4)	1(4)	-1(4)
C17'	51(7)	26(5)	29(6)	-7(4)	-7(5)	-2(5)
C16'	24(5)	22(5)	33(6)	-4(4)	-8(4)	-1(4)
C10'	38(6)	18(5)	62(8)	-20(5)	4(6)	-8(4)
C7	20(5)	19(5)	52(6)	-5(5)	6(5)	-7(4)
C6	20(4)	22(3)	54(5)	-6(3)	9(3)	-3(3)
C18	46(6)	15(4)	38(6)	-4(4)	-2(5)	-7(4)
C14	23(5)	22(5)	38(6)	-7(4)	3(4)	0(4)
C1	32(6)	34(5)	40(6)	-8(5)	1(5)	-7(4)
C2	20(4)	22(3)	54(5)	-6(3)	9(3)	-3(3)
C15'	31(6)	21(5)	37(6)	-11(4)	6(5)	-7(4)
C21'	28(6)	38(6)	30(6)	-8(5)	-2(5)	-2(4)
C3'	58(8)	21(5)	51(7)	-17(5)	-8(6)	6(5)
C9'	39(6)	24(5)	53(7)	-24(5)	2(6)	-4(4)
C11'	34(6)	26(5)	55(7)	-20(5)	10(5)	-11(4)
C5'	54(7)	26(5)	36(6)	-11(5)	5(6)	-19(5)
C15	21(5)	30(5)	47(6)	-8(5)	7(5)	-13(4)
C6'	37(6)	28(5)	41(6)	-17(5)	16(5)	-13(5)
C5	30(6)	32(5)	47(6)	-14(5)	-10(5)	-4(4)
C7'	34(6)	25(5)	39(6)	-17(5)	6(5)	1(4)
C21	44(6)	27(5)	50(7)	-11(5)	-12(6)	1(5)
C20'	51(7)	38(6)	34(6)	-13(5)	3(5)	1(5)
C18'	67(8)	29(5)	34(6)	-11(5)	-21(6)	-8(5)
C11	28(6)	23(5)	57(7)	-13(5)	-11(5)	0(4)
C12	37(6)	20(5)	49(7)	-10(5)	1(5)	-3(4)
C20	51(7)	35(6)	52(7)	-29(5)	8(6)	-9(5)
C4'	53(7)	35(6)	49(7)	-22(5)	-2(6)	2(5)
C10	34(6)	28(5)	41(7)	-4(5)	0(5)	-20(5)
C8'	49(7)	28(5)	45(7)	-20(5)	2(6)	7(5)
C3	36(6)	34(6)	52(7)	-7(5)	-7(5)	2(5)
C2'	42(7)	30(6)	67(8)	-26(6)	16(6)	-10(5)
C12'	50(7)	18(5)	39(6)	-5(4)	8(5)	-11(4)
C8	39(6)	30(5)	38(6)	-9(5)	0(5)	-1(4)
C1'	47(7)	23(5)	63(8)	-18(5)	9(6)	-4(5)
C19	45(7)	39(6)	34(6)	-7(5)	5(5)	-11(5)
C4	43(7)	38(6)	47(7)	-13(5)	-9(5)	-8(5)
C13	40(7)	59(7)	61(8)	-6(6)	-17(6)	9(6)
C19'	63(8)	44(6)	31(6)	-14(5)	0(6)	7(6)
C13'	60(8)	41(6)	69(8)	-11(6)	-4(7)	15(6)

O2'	125(9)	79(6)	59(6)	-10(5)	-13(6)	11(6)
C23'	230(30)	170(20)	75(14)	-57(14)	52(15)	-63(18)
C22'	190(20)	420(40)	150(20)	-150(30)	56(18)	-200(30)
C24'	210(30)	390(50)	140(20)	-130(30)	30(20)	80(30)
O2	70(9)	114(12)	165(17)	-14(12)	-29(12)	-18(9)
C23	70(9)	114(12)	165(17)	-14(12)	-29(12)	-18(9)
C22	70(9)	114(12)	165(17)	-14(12)	-29(12)	-18(9)
C24	70(9)	114(12)	165(17)	-14(12)	-29(12)	-18(9)

Table 4 Bond Lengths for **17a**.

**Atom**	**Atom**	**Length/Å**	**Atom**	**Atom**	**Length/Å**
Br1'	C3'	1.914(10)	C16'	C21'	1.371(12)
Br1	C3	1.917(10)	C10'	C9'	1.535(14)
Br2	C19	1.901(9)	C7	C6	1.438(13)
Br2'	C19'	1.890(10)	C7	C8	1.377(12)
O1'	C10'	1.225(11)	C6	C1	1.398(12)
O1	C10	1.240(11)	C6	C5	1.398(13)
N2	C9	1.297(11)	C18	C19	1.345(12)
N2	C11	1.459(10)	C14	C15	1.351(12)
N4	C17	1.355(11)	C14	C12	1.511(12)
N4	C15	1.368(11)	C1	C2	1.383(13)
N2'	C9'	1.263(11)	C2	C3	1.325(13)
N2'	C11'	1.454(12)	C21'	C20'	1.372(12)
N1	C1	1.370(12)	C3'	C4'	1.381(14)
N1	C8	1.354(11)	C3'	C2'	1.370(14)
N3'	C10'	1.318(13)	C9'	C7'	1.437(13)
N3'	C12'	1.447(12)	C11'	C12'	1.497(13)
N3'	C13'	1.456(13)	C5'	C6'	1.436(13)
N3	C12	1.459(12)	C5'	C4'	1.349(13)
N3	C10	1.343(12)	C6'	C7'	1.426(13)
N3	C13	1.452(12)	C6'	C1'	1.394(13)
N4'	C17'	1.367(11)	C5	C4	1.366(13)
N4'	C15'	1.378(10)	C7'	C8'	1.387(13)
C16	C17	1.423(11)	C21	C20	1.381(13)
C16	C14	1.425(12)	C20'	C19'	1.393(14)
C16	C21	1.385(12)	C18'	C19'	1.362(13)
N1'	C8'	1.334(12)	C11	C12	1.509(13)
N1'	C1'	1.395(13)	C20	C19	1.401(13)
C17	C18	1.394(12)	C3	C4	1.387(13)
C9	C7	1.446(12)	C2'	C1'	1.368(14)
C9	C10	1.515(13)	O2'	C23'	1.415(14)

C14'	C16'	1.424(12)	C23'	C22'	1.516(16)
C14'	C15'	1.357(11)	C23'	C24'	1.547(18)
C14'	C12'	1.512(11)	O2	C23	1.42(4)
C17'	C16'	1.421(12)	C23	C22	1.467(18)
C17'	C18'	1.369(12)	C23	C24	1.528(19)

Table 5 Bond Angles for **17a**.

**Atom**	**Atom**	**Atom**	**Angle/°**	**Atom**	**Atom**	**Atom**	**Angle/°**
C9	N2	C11	116.3(8)	C2'	C3'	C4'	123.0(11)
C17	N4	C15	109.3(7)	N2'	C9'	C10'	122.9(10)
C9'	N2'	C11'	117.2(9)	N2'	C9'	C7'	119.0(9)
C8	N1	C1	108.3(8)	C7'	C9'	C10'	118.1(9)
C10'	N3'	C12'	122.2(9)	N2'	C11'	C12'	114.1(8)
C10'	N3'	C13'	117.2(9)	C4'	C5'	C6'	119.1(10)
C12'	N3'	C13'	119.9(9)	C14	C15	N4	109.7(8)
C10	N3	C12	120.2(8)	C7'	C6'	C5'	134.6(9)
C10	N3	C13	119.7(9)	C1'	C6'	C5'	116.8(10)
C13	N3	C12	118.9(8)	C1'	C6'	C7'	108.6(9)
C17'	N4'	C15'	109.5(7)	C4	C5	C6	120.2(9)
C17	C16	C14	105.8(7)	C6'	C7'	C9'	126.3(9)
C21	C16	C17	118.2(8)	C8'	C7'	C9'	128.5(10)
C21	C16	C14	135.9(8)	C8'	C7'	C6'	104.9(8)
C8'	N1'	C1'	109.4(9)	C20	C21	C16	120.3(9)
N4	C17	C16	107.6(8)	C21'	C20'	C19'	120.5(9)
N4	C17	C18	130.5(8)	C19'	C18'	C17'	117.3(9)
C18	C17	C16	121.8(8)	N2	C11	C12	112.7(7)
N2	C9	C7	119.2(8)	N3	C12	C14	111.2(7)
N2	C9	C10	122.8(8)	N3	C12	C11	108.3(8)
C7	C9	C10	118.0(9)	C11	C12	C14	113.3(8)
C16'	C14'	C12'	126.0(8)	C21	C20	C19	119.0(9)
C15'	C14'	C16'	108.1(7)	C5'	C4'	C3'	120.8(10)
C15'	C14'	C12'	125.9(8)	O1	C10	N3	123.2(9)
N4'	C17'	C16'	107.3(8)	O1	C10	C9	120.9(9)
N4'	C17'	C18'	130.9(9)	N3	C10	C9	115.9(9)
C18'	C17'	C16'	121.8(9)	N1'	C8'	C7'	110.8(10)
C17'	C16'	C14'	106.2(8)	C2	C3	Br1	118.9(7)
C21'	C16'	C14'	134.5(8)	C2	C3	C4	123.5(9)
C21'	C16'	C17'	119.3(8)	C4	C3	Br1	117.6(8)
O1'	C10'	N3'	123.5(10)	C1'	C2'	C3'	115.9(11)
O1'	C10'	C9'	121.2(10)	N3'	C12'	C14'	111.5(7)
N3'	C10'	C9'	115.4(9)	N3'	C12'	C11'	108.8(8)

C6	C7	C9	126.1(9)	C11'	C12'	C14'	111.3(7)
C8	C7	C9	128.1(9)	N1	C8	C7	110.9(9)
C8	C7	C6	105.4(8)	C6'	C1'	N1'	106.3(10)
C1	C6	C7	106.8(9)	C2'	C1'	N1'	129.3(10)
C1	C6	C5	116.7(9)	C2'	C1'	C6'	124.4(10)
C5	C6	C7	136.5(8)	C18	C19	Br2	119.2(7)
C19	C18	C17	117.2(8)	C18	C19	C20	123.5(9)
C16	C14	C12	125.3(8)	C20	C19	Br2	117.3(7)
C15	C14	C16	107.6(8)	C5	C4	C3	119.3(10)
C15	C14	C12	126.9(8)	C20'	C19'	Br2'	118.4(8)
N1	C1	C6	108.5(8)	C18'	C19'	Br2'	119.5(8)
N1	C1	C2	128.1(9)	C18'	C19'	C20'	122.1(9)
C2	C1	C6	123.4(10)	O2'	C23'	C22'	113(2)
C3	C2	C1	116.7(9)	O2'	C23'	C24'	100.8(17)
C14'	C15'	N4'	108.9(8)	C22'	C23'	C24'	121.3(18)
C16'	C21'	C20'	119.0(9)	O2	C23	C22	105(2)
C4'	C3'	Br1'	116.9(8)	O2	C23	C24	112(3)
C2'	C3'	Br1'	120.1(9)	C22	C23	C24	137(3)

Table 6 Torsion Angles for **17a**.

**A**	**B**	**C**	**D**	**Angle/°**	**A**	**B**	**C**	**D**	**Angle/°**
Br1'	C3'	C4'	C5'	179.0(7)	C15'	C14'	C16'	C17'	1.6(10)
Br1'	C3'	C2'	C1'	-178.8(7)	C15'	C14'	C16'	C21'	-178.3(10)
Br1	C3	C4	C5	-177.2(7)	C15'	C14'	C12'	N3'	18.0(14)
O1'	C10'	C9'	N2'	164.7(9)	C15'	C14'	C12'	C11'	-103.7(11)
O1'	C10'	C9'	C7'	-16.3(12)	C21'	C20'	C19'	Br2'	179.3(8)
N2	C9	C7	C6	-7.9(13)	C21'	C20'	C19'	C18'	0.3(16)
N2	C9	C7	C8	163.7(9)	C3'	C2'	C1'	N1'	178.7(9)
N2	C9	C10	O1	161.8(8)	C3'	C2'	C1'	C6'	-1.4(14)
N2	C9	C10	N3	-18.4(12)	C9'	N2'	C11'	C12'	34.5(11)
N2	C11	C12	N3	-55.4(10)	C9'	C7'	C8'	N1'	-175.4(8)
N2	C11	C12	C14	68.5(10)	C11'	N2'	C9'	C10'	-0.7(12)
N4	C17	C18	C19	-177.8(10)	C11'	N2'	C9'	C7'	-179.8(7)
N2'	C9'	C7'	C6'	-13.4(14)	C5'	C6'	C7'	C9'	-6.7(16)
N2'	C9'	C7'	C8'	159.2(9)	C5'	C6'	C7'	C8'	179.3(9)
N2'	C11'	C12'	N3'	-50.5(10)	C5'	C6'	C1'	N1'	179.9(7)
N2'	C11'	C12'	C14'	72.7(10)	C5'	C6'	C1'	C2'	0.1(13)
N1	C1	C2	C3	-179.0(9)	C15	N4	C17	C16	0.9(10)
N3'	C10'	C9'	N2'	-15.4(12)	C15	N4	C17	C18	-179.0(9)
N3'	C10'	C9'	C7'	163.7(8)	C15	C14	C12	N3	20.5(14)
N4'	C17'	C16'	C14'	-0.6(10)	C15	C14	C12	C11	-101.8(11)

N4'	C17'	C16'	C21'	179.4(8)	C6'	C5'	C4'	C3'	0.8(13)
N4'	C17'	C18'	C19'	178.7(10)	C6'	C7'	C8'	N1'	-1.7(10)
C16	C17	C18	C19	2.3(14)	C5	C6	C1	N1	-179.6(8)
C16	C14	C15	N4	1.5(11)	C5	C6	C1	C2	-0.3(13)
C16	C14	C12	N3	-166.5(8)	C7'	C6'	C1'	N1'	-2.8(10)
C16	C14	C12	C11	71.1(11)	C7'	C6'	C1'	C2'	177.3(9)
C16	C21	C20	C19	0.5(15)	C21	C16	C17	N4	177.8(8)
C17	N4	C15	C14	-1.5(11)	C21	C16	C17	C18	-2.3(13)
C17	C16	C14	C15	-0.9(10)	C21	C16	C14	C15	-178.1(11)
C17	C16	C14	C12	-175.0(9)	C21	C16	C14	C12	7.8(17)
C17	C16	C21	C20	0.8(14)	C21	C20	C19	Br2	178.5(7)
C17	C18	C19	Br2	-179.9(7)	C21	C20	C19	C18	-0.5(16)
C17	C18	C19	C20	-0.9(15)	C18'	C17'	C16'	C14'	177.8(9)
C9	N2	C11	C12	36.3(11)	C18'	C17'	C16'	C21'	-2.2(14)
C9	C7	C6	C1	169.3(8)	C11	N2	C9	C7	-176.5(7)
C9	C7	C6	C5	-7.1(16)	C11	N2	C9	C10	1.1(11)
C9	C7	C8	N1	-169.7(8)	C12	N3	C10	O1	174.7(8)
C14'	C16'	C21'	C20'	-177.4(10)	C12	N3	C10	C9	-5.0(11)
C17'	N4'	C15'	C14'	1.7(11)	C12	C14	C15	N4	175.5(8)
C17'	C16'	C21'	C20'	2.6(13)	C4'	C3'	C2'	C1'	2.5(13)
C17'	C18'	C19'	Br2'	-178.9(8)	C4'	C5'	C6'	C7'	-176.1(9)
C17'	C18'	C19'	C20'	0.2(15)	C4'	C5'	C6'	C1'	0.3(12)
C16'	C14'	C15'	N4'	-2.1(10)	C10	N3	C12	C14	-85.5(10)
C16'	C14'	C12'	N3'	-165.2(9)	C10	N3	C12	C11	39.7(10)
C16'	C14'	C12'	C11'	73.0(12)	C10	C9	C7	C6	174.4(8)
C16'	C17'	C18'	C19'	0.8(15)	C10	C9	C7	C8	-14.0(13)
C16'	C21'	C20'	C19'	-1.7(15)	C8'	N1'	C1'	C6'	1.8(10)
C10'	N3'	C12'	C14'	-87.1(11)	C8'	N1'	C1'	C2'	-178.3(9)
C10'	N3'	C12'	C11'	36.1(11)	C2'	C3'	C4'	C5'	-2.3(14)
C10'	C9'	C7'	C6'	167.5(8)	C12'	N3'	C10'	O1'	174.8(8)
C10'	C9'	C7'	C8'	-19.9(13)	C12'	N3'	C10'	C9'	-5.1(12)
C7	C9	C10	O1	-20.5(12)	C12'	C14'	C16'	C17'	-175.6(9)
C7	C9	C10	N3	159.2(8)	C12'	C14'	C16'	C21'	4.5(17)
C7	C6	C1	N1	3.2(10)	C12'	C14'	C15'	N4'	175.2(8)
C7	C6	C1	C2	-177.5(8)	C8	N1	C1	C6	-1.2(10)
C7	C6	C5	C4	175.9(10)	C8	N1	C1	C2	179.5(9)
C6	C7	C8	N1	3.3(10)	C8	C7	C6	C1	-3.9(10)
C6	C1	C2	C3	1.8(14)	C8	C7	C6	C5	179.7(10)
C6	C5	C4	C3	-0.8(14)	C1'	N1'	C8'	C7'	-0.1(10)
C14	C16	C17	N4	0.0(10)	C1'	C6'	C7'	C9'	176.7(8)
C14	C16	C17	C18	179.9(8)	C1'	C6'	C7'	C8'	2.7(10)
C14	C16	C21	C20	177.8(10)	C13	N3	C12	C14	82.2(10)
C1	N1	C8	C7	-1.4(11)	C13	N3	C12	C11	-152.6(8)

C1	C6	C5	C4	-0.2(13)	C13	N3	C10	O1	7.1(13)
C1	C2	C3	Br1	176.8(7)	C13	N3	C10	C9	-172.6(8)
C1	C2	C3	C4	-2.9(15)	C13'	N3'	C10'	O1'	4.6(13)
C2	C3	C4	C5	2.5(16)	C13'	N3'	C10'	C9'	-175.4(8)
C15'	N4'	C17'	C16'	-0.6(10)	C13'	N3'	C12'	C14'	82.9(10)
C15'	N4'	C17'	C18'	-178.8(10)	C13'	N3'	C12'	C11'	-153.9(8)

Table 7 Hydrogen Atom Coordinates (Å×10^4) and Isotropic Displacement Parameters (Å^2×10^3) for **17a**.

**Atom**	***x***	***y***	***z***	**U(eq)**
H4	506	6969	6597	42
H1	6066	6240	4879	50
H4'	3439	8041	7431	45
H1'	3043	6990	3324	57
H18	-958	6948	8197	41
H2	7236	5357	6328	42
H15'	3724	6433	6977	36
H21'	1024	5221	9804	40
H11A	1077	3989	7912	45
H11B	639	5055	8165	45
H5'	-446	5852	5808	46
H15	1822	8156	5480	41
H5	5540	8341	7163	42
H21	249	10389	7442	48
H20'	295	6384	10636	50
H18'	2182	8700	8849	49
H11C	3274	10858	5974	42
H11D	2599	10122	6895	42
H12	1456	10794	5695	44
H20	-1122	9744	8719	52
H4'A	-1701	6633	4770	54
H8'	3764	6006	4711	49
H2'	928	7597	2723	56
H12'	2407	4215	8690	45
H8	4791	7789	4330	44
H4A	6742	7086	8063	51
H13D	1011	10362	4303	84
H13E	2134	10309	3572	84
H13F	1785	11351	3882	84
H13A	4629	3319	7496	91
H13B	4290	3672	8400	91

H13C	4961	4441	7496	91
H2'A	6299	1291	6899	138
H23'	6409	111	8132	191
H22A	4394	530	7674	347
H22B	4108	669	8671	347
H22C	4691	-434	8546	347
H24A	5461	1397	9220	380
H24B	6587	1825	8527	380
H24C	6647	667	9256	380
H2A	3712	3722	10040	182
H23	5026	4739	9033	145
H22D	5084	6134	9126	182
H22E	5672	5660	10035	182
H22F	4299	5839	10106	182
H24D	5460	3036	10474	182
H24E	6531	3686	9888	182
H24F	5744	3220	9397	182

Table 8 Atomic Occupancy for **17a**.

**Atom**	***Occupancy***	**Atom**	***Occupancy***	**Atom**	***Occupancy***
O2	0.5	H2A	0.5	C23	0.5
H23	0.5	C22	0.5	H22D	0.5
H22E	0.5	H22F	0.5	C24	0.5
H24D	0.5	H24E	0.5	H24F	0.5

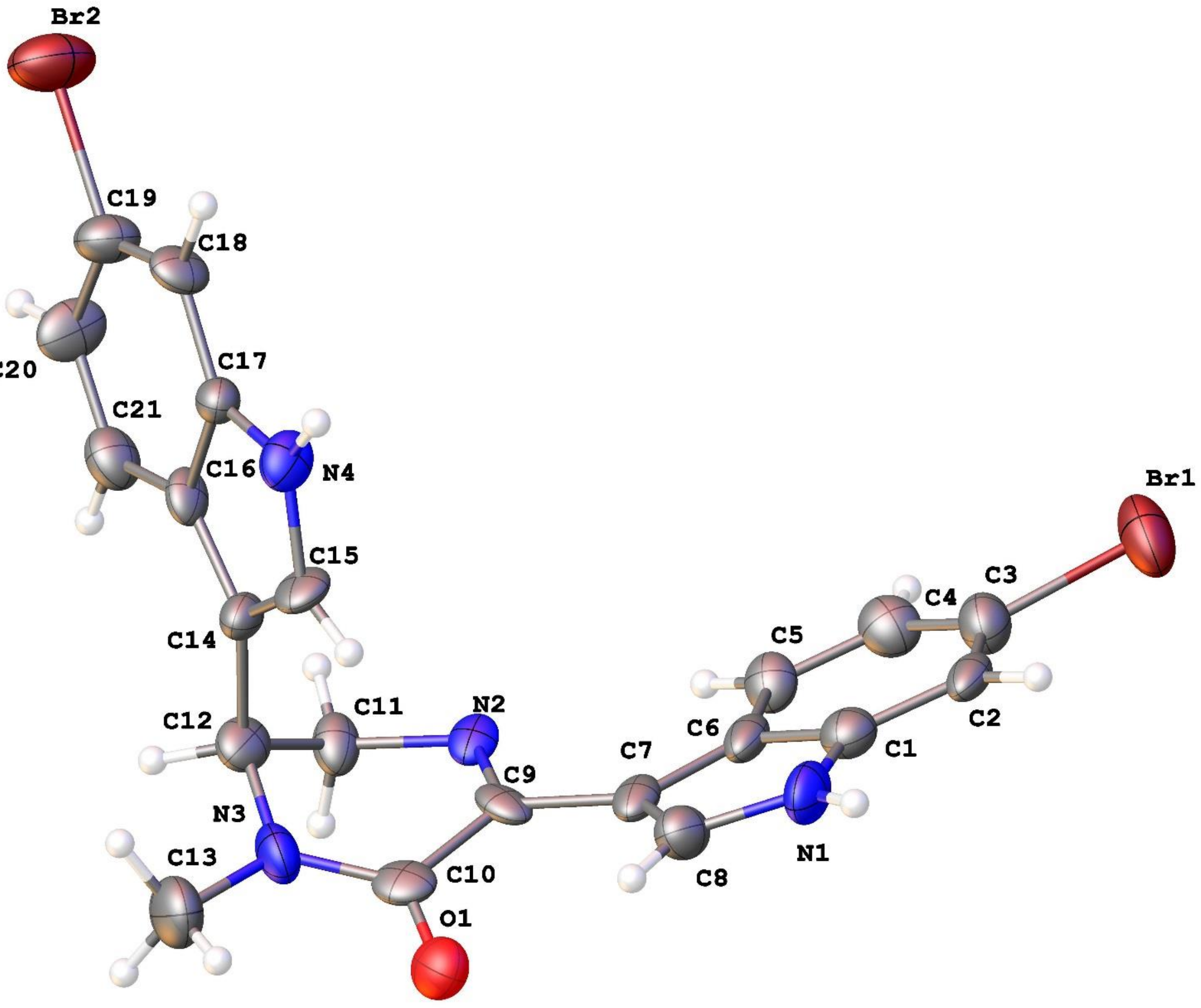

Figure 1. Molecular structure of **17a** presented as ADP ellipsoids at probability is equal to 50%.

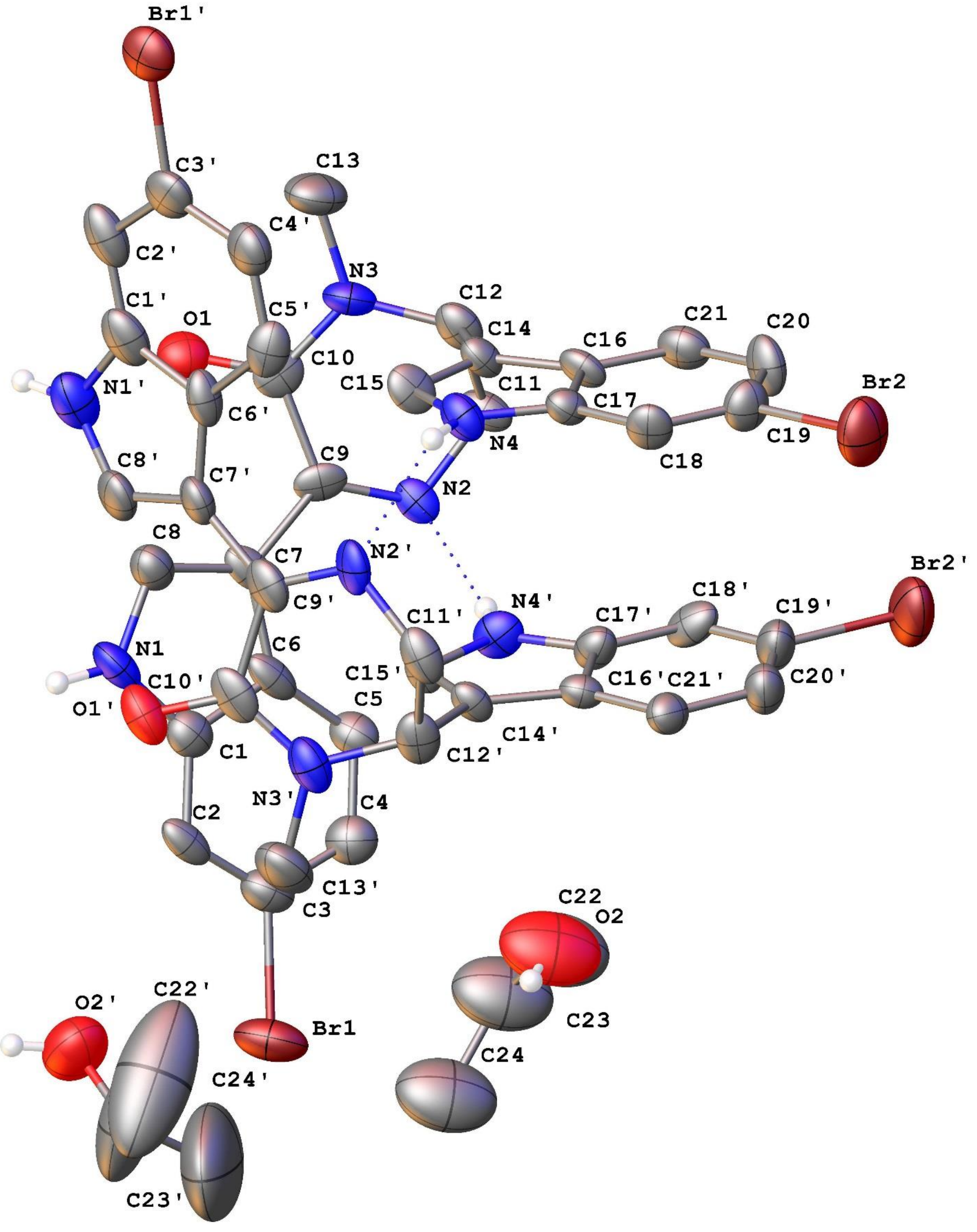

Figure 2. General view of independent part of unit cell in ADP ellipsoids is equal to 50%.

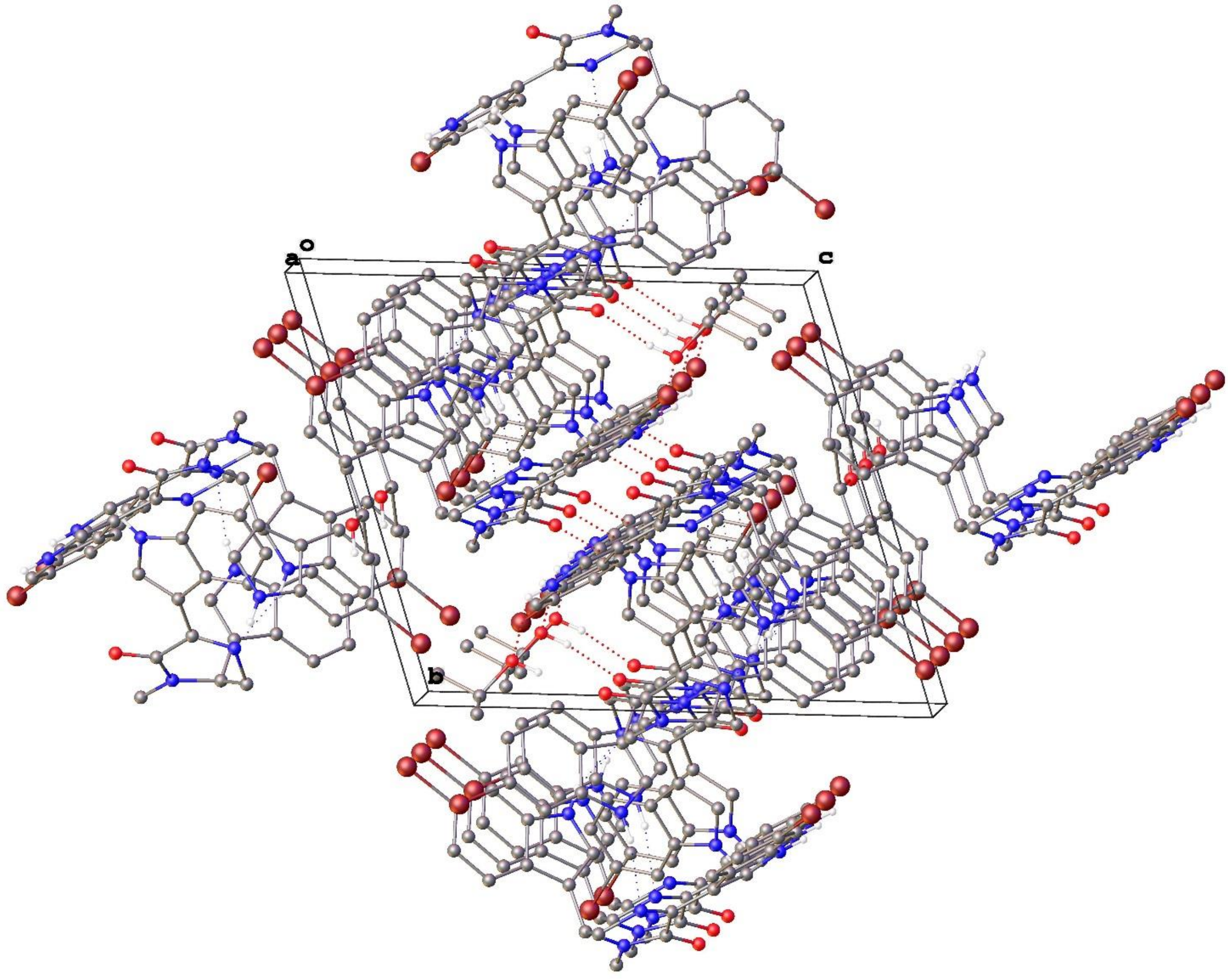

Figure 3. Crystal packing of **17a**.
